# Opioid‐free vs. opioid‐inclusive anaesthesia with or without regional anaesthesia for postoperative pain: a systematic review with network meta‐analysis of randomised controlled trials

**DOI:** 10.1111/anae.70121

**Published:** 2026-01-05

**Authors:** Clístenes C. de Carvalho, Kariem El‐Boghdadly, Idrys H. L. Guedes, Maria Vitória M. Dantas, Danusa P. B. Tomé, Isabella B. Ramos, Clarissa S. H. Gomes, Guilherme K. P. A. Alves, Arthur P. Bezerra, Jayme M. Santos Neto, Jaideep J. Pandit, Leandro G. Braz

**Affiliations:** ^1^ Department of Anaesthesia and Peri‐operative Medicine Guy's and St Thomas' NHS Foundation Trust London UK; ^2^ Academic Unit of Medicine Federal University of Campina Grande Campina Grande Brazil; ^3^ Department of Surgical Specialties and Anaesthesiology São Paulo State University, Medical School Botucatu Brazil; ^4^ Centre for Human and Applied Physiological Sciences King's College London London UK; ^5^ Centre for Biological and Health Sciences Federal University of Campina Grande Campina Grande Brazil; ^6^ Hospital das Clínicas, Universidade Federal de Pernambuco Recife PE Brazil; ^7^ Nuffield Department of Anaesthetics Oxford University Hospitals NHS Foundation Trust Oxford UK; ^8^ Nuffield Department of Clinical Neurosciences University of Oxford Oxford UK

**Keywords:** analgesia, opioid‐free anaesthesia, postoperative pain, regional anaesthesia, systematic review

## Abstract

**Introduction:**

Concerns about opioid‐related adverse effects have increased interest in opioid‐free anaesthesia, but the benefits compared with opioid‐inclusive techniques, especially in the presence of regional anaesthesia, remain uncertain.

**Methods:**

We undertook a systematic review with a network meta‐analysis of randomised controlled trials comparing six strategies in adults: opioid‐free anaesthesia and opioid‐inclusive anaesthesia using remifentanil alone or other opioids, each with or without regional anaesthesia. Primary outcome was postoperative pain. Secondary outcomes were: postoperative opioid use; post‐anaesthesia care unit discharge time; hospital duration of stay; and incidence of complications.

**Results:**

We included 885 trials from 59 countries. Techniques incorporating regional anaesthesia consistently ranked highest for postoperative pain. Regional anaesthesia combined with an opioid‐free intra‐operative strategy achieved some of the highest surface under the cumulative ranking curve values for pain at 2 h, 12 h and 48 h (93%, 85% and 75%, all low certainty). When regional anaesthesia was used, differences between opioid‐free and opioid‐inclusive techniques were minimal (moderate certainty). For opioid consumption, regional anaesthesia with an opioid‐free strategy ranked best at 2 h (moderate certainty), 12 h (low certainty) and 48 h (low certainty), with surface under the cumulative ranking curve values > 98%. Techniques without regional anaesthesia were associated with higher pain scores and greater opioid requirements. Opioid‐free approaches, especially when combined with regional techniques, were associated with lower rates of postoperative nausea and vomiting.

**Discussion:**

Regional anaesthesia was the key determinant of improved postoperative pain control, and intra‐operative opioids added little additional benefit when regional techniques provided adequate coverage. Without regional anaesthesia, neither opioid‐free nor opioid‐inclusive strategies showed consistent analgesic superiority. However, opioid‐free techniques reduced postoperative nausea and vomiting. These findings support preferential use of regional anaesthesia where feasible and suggest that avoiding intra‐operative opioids may facilitate recovery, particularly when regional techniques are employed effectively.

## Plain Language Summary

**What we did:**

We looked at many studies from around the world that compared different ways of giving anaesthesia (the medicine that keeps you asleep during surgery). We compared six types of anaesthesia, including some that used opioids (strong pain medicines) and some that did not. We also looked at whether a special kind of numbing, called regional anaesthesia, was used. We checked how well each method worked for pain after surgery and other outcomes like sickness and time in hospital.

**Why did we do it:**

Doctors are worried about the side effects of opioids, so they want to know if people can stay comfortable after surgery without using them. We wanted to find out which combinations of anaesthesia give the best pain control and the fewest problems after surgery.

**What we found:**

The best pain control happened when regional anaesthesia was used, no matter what other medicines were given. When regional anaesthesia was already helping, using or avoiding opioids during the operation didn't make much difference to pain afterwards. People who did not get regional anaesthesia had more pain and needed more opioids later. Opioid‐free techniques (especially when combined with regional anaesthesia) led to less sickness and vomiting after surgery. Overall, the results show that regional anaesthesia is the most important part for good pain control. Avoiding opioids during the operation may also help people feel better afterwards, especially when regional anaesthesia is used.

## Introduction

Opioids have long been the mainstay of peri‐operative pain management [1, 2]. However, their use is associated with a range of adverse effects including postoperative nausea and vomiting (PONV); sedation; dizziness; pruritus; urinary retention; respiratory depression; impaired recovery; and the risk of persistent opioid use [2, 3, 4]. Moreover, a recent study has questioned the assumption that opioids reliably enhance wellbeing in the peri‐operative setting, particularly among opioid‐naïve patients [5]. These concerns have prompted increasing interest in strategies that minimise or eliminate intra‐operative opioid administration [2, 3, 6].

Opioid‐free anaesthesia and opioid‐sparing techniques, often delivered as part of a multimodal analgesic approach, have been proposed as alternatives to conventional opioid‐inclusive anaesthesia [2, 3, 6]. These strategies frequently incorporate regional or local anaesthesia alongside systemic drugs [3, 6]. While several randomised controlled trials and meta‐analyses have suggested that opioid‐free anaesthesia may reduce opioid‐related complications without impairing postoperative analgesia, many of these analyses have grouped diverse anaesthetic approaches into broad categories [4, 7, 8, 9]. This limits their ability to determine whether observed outcomes are influenced by other factors, such as the application of regional anaesthesia or the specific choice of opioid. For example, techniques relying solely on remifentanil intra‐operatively may worsen postoperative pain outcomes and increase opioid consumption compared with those using relatively longer‐acting opioids such as sufentanil or alfentanil [10, 11]. This effect reflects the pharmacology of remifentanil: its rapid offset can result in moderate‐to‐severe pain after discontinuation and at high doses it has been implicated in opioid‐induced hyperalgesia and acute opioid tolerance, both of which manifest as increased opioid requirements [12]. These considerations justify evaluating remifentanil separately from other intra‐operative opioids. Similarly, the addition of regional anaesthesia may significantly improve postoperative recovery as an independent contributor [13]. These differences are unlikely to be captured when all opioid‐free or opioid‐inclusive strategies are analysed as single, homogeneous categories.

Network meta‐analysis enables the simultaneous comparison of multiple interventions, offering a broader understanding of anaesthetic strategies than pairwise analyses. It is particularly suited to current gaps, as it can disentangle the role of regional anaesthesia; assess the impact of specific opioids; and evaluate combinations rather than treating techniques as homogeneous groups. Here, we aimed to characterise the effectiveness and safety of opioid‐free and opioid‐inclusive techniques, with or without regional anaesthesia, in adult patients undergoing surgery, clarifying how their individual components and combinations influence postoperative outcomes.

## Methods

This systematic review was designed in accordance with established methodological standards and reported following PRISMA‐NMA guidelines [14, 15, 16].

Eligible studies were randomised controlled trials involving patients aged ≥ 16 y, reporting on at least one of the following outcomes: postoperative pain scores; postoperative opioid consumption; time to discharge from the post‐anaesthesia care unit (PACU); time to hospital discharge; and incidence of postoperative complications. Included studies compared different anaesthetic techniques, specifically: opioid‐free anaesthesia with or without local/regional anaesthesia; opioid‐inclusive anaesthesia using remifentanil only with or without local/regional anaesthesia; and opioid‐inclusive anaesthesia using opioids other than remifentanil with or without local/regional anaesthesia. Studies were not included if published in languages other than English, Spanish, French or Portuguese; if relevant outcome data could not be extracted; or if systematic differences existed between study arms beyond the anaesthetic technique under investigation.

We conducted searches in PubMed, Embase, Web of Science, Cochrane Central Register of Controlled Trials (CENTRAL), LILACS, and SciELO on 20 March 2022, without date or language restrictions. The same strategies were applied for an updated search on 15 January 2025. Full search strategies for each database are in online Supporting Information Appendix S1.

References were imported into EPPI Reviewer Web (beta) (UCL Social Research Institute, London, UK) for title/abstract and full‐text screening. Eligibility criteria were applied to determine study inclusion. Independent reviewers conducted all steps in duplicate, from screening and risk of bias assessment to data extraction. Disagreements were resolved through discussion and consensus; a third reviewer adjudicated unresolved cases. Authors were contacted when data were missing or incongruent. If no response was received after three attempts over one month, the relevant outcome or study was omitted. Data were recorded in Microsoft Excel (Microsoft Corporation, Redmond, WA, USA) using a standardised extraction form, which was piloted in five studies and refined accordingly. Data from each study group (i.e. each anaesthetic technique) were processed separately for network analyses.

We collected data on: author(s) names; year of publication; study design; population characteristics; mean age; mean BMI; mean weight; mean height; sex distribution; ASA physical status; study setting; and country where the study was conducted. We also extracted information on the risk of postoperative pain; number of patients allocated randomly and analysed in each arm; intervention applied and classified according to the categories described in the ‘interventions’ section; drugs used for hypnosis and their doses; and use of dexmedetomidine for anaesthesia maintenance. For continuous outcomes, we recorded the mean and standard deviation in each arm, as well as the mean difference and standard error between groups. For categorical outcomes, we extracted the number of events and number of patients per arm, along with the relative risk and standard error between groups.

The primary outcome was postoperative pain. Secondary outcomes included postoperative opioid consumption (converted to oral morphine equivalent doses); time to discharge from PACU; duration of hospital stay; and postoperative complications, including PONV, dizziness, pruritus and urinary retention. Most studies assessed postoperative pain using numerical or visual analogue scales on a 0–10 range. When data were presented on a 0–100 scale, values were standardised by dividing by 10. A minority of studies employed other validated instruments, such as the Wong‐Baker FACES Pain Rating Scale, which are also anchored on a 0–10 scale. Given the variability in assessment timing, we selected the time‐points most consistently reported: 2 h; 12 h; 24 h; and 48 h postoperatively.

We generated a network graph to illustrate the structure of the evidence base. Nodes represent individual interventions, while connecting lines indicate direct comparisons between techniques. The size of each node is proportional to the number of patients allocated to that intervention and the thickness of each line reflects the number of randomised controlled trials informing the corresponding comparison. We assessed the risk of bias for each outcome using the Cochrane Risk of Bias 2 (RoB 2) tool [17].

We performed Bayesian random‐effects network meta‐analyses using the ‘gemtc’ [18] package in R (R Foundation for Statistical Computing, Vienna, Austria). Per‐protocol data were extracted or derived from the included studies. Effect sizes, standard errors (SE) and 95% credible intervals (CrIs) were estimated for each comparison, integrating both direct and indirect evidence. Multi‐arm trials were handled by dividing them into multiple pairwise comparisons using a single reference group. To rank interventions, we calculated surface under the cumulative ranking curve (SUCRA) values, which express the probability that each intervention is among the most effective. We constructed league tables, network forest plots and rankograms to display comparative effect estimates and associated uncertainty.

Heterogeneity was assessed qualitatively and quantitatively. For each comparison, we estimated the I^2^ and τ^2^ statistics, and Cochran's Q test was used to explore global heterogeneity. Consistency between direct and indirect evidence was evaluated using local and global approaches, including the node‐splitting method, analysis of heterogeneity and Q statistics. Sensitivity and subgroup analyses were conducted based on pre‐specified hypotheses, including the use of dexmedetomidine; expected postoperative pain risk (according to patient and procedural characteristics); and the risk of bias in individual studies. Given growing concerns about data reliability and reproducibility in clinical trials, country of origin was also considered in subgroup and sensitivity analyses.

We assessed selective publication using comparison‐adjusted funnel plots, under the assumption that studies evaluating novel techniques and reporting positive results were more likely to be published. Egger's tests were applied to detect funnel plot asymmetry, with a significance threshold set at p < 0.1 due to the limited statistical power. Quality of the evidence was assessed using the Confidence in Network Meta‐Analysis (CINeMA) framework, based on the GRADE approach [19, 20, 21, 22]. This method considers factors related to: study design; within‐study risk of bias; inconsistency; indirectness; imprecision; publication bias; and incoherence between direct and indirect evidence.

## Results

**Figure 1:**
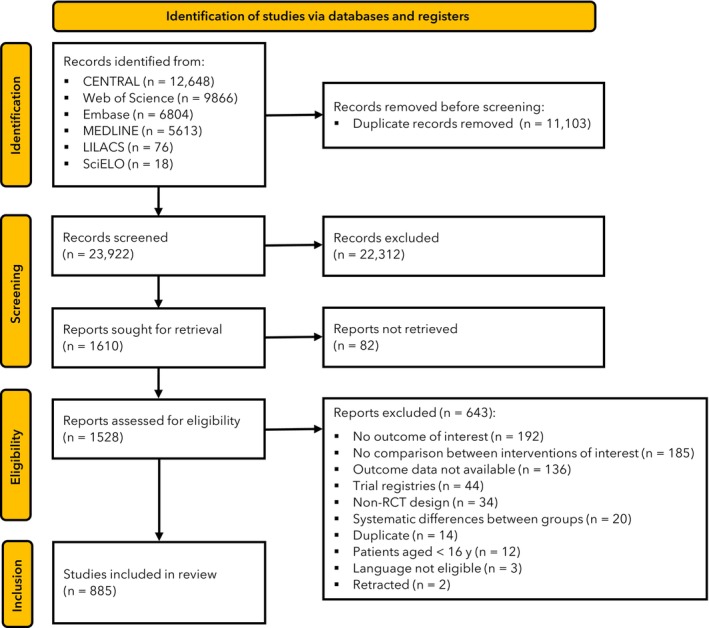
Study flow diagram.

Our electronic search identified 35,025 articles, of which 885 randomised controlled trials were included (Fig. 1); the full list is in online Supporting Information Appendix S2. The included studies were from 59 countries and encompassed diverse populations, surgical settings and anaesthetic strategies. Most trials involved elective procedures and general, non‐specified patient populations, though some targeted specific groups such as women, older adults or those living with obesity. Anaesthetic protocols varied widely in terms of induction drugs, maintenance strategies and adjuncts such as dexmedetomidine. Summary details of study characteristics are in Table 1.

**Table 1:** Summary of study characteristics across all included studies. Values are number, median (IQR [range]) or number (proportion).

Studies	885
Total patients; n	74,880
Patients per study; n	60 (48–91 [18–2438])
Age; y	49 (42–59 [18–88 s])
BMI; kg m^‐2^	25.6 (23.6–27.6 [20.0–60.0])
Study population	
General/non‐specific (n = 627)	52,812 (70.5%)
Non‐pregnant women (n = 174)	14,832 (19.8%)
Older people (n = 29)	3287 (4.4%)
Men (n = 29)	2137 (2.9%)
Pregnant women (n = 13)	1045 (1.4%)
Patients living with obesity (n = 10)	787 (1.1%)
Operative urgency	
Elective (n = 868)	73,954 (98.7%)
Mixed/unclear (n = 9)	512 (0.7%)
Urgent (n = 6)	414 (0.6%)
Regional anaesthesia (n = 710)	31,719 (42.4%)
Opioid‐free techniques (n = 199)	9343 (12.5%)
Predicted risk of postoperative pain*	
Low (n = 338)	25,406 (33.9%)
Variable (n = 297)	26,846 (35.8%)
High (n = 246)	22,628 (30.2%)
Anaesthesia maintenance	
Inhalational anaesthesia (n = 554)	48,938 (65.3%)
Mixed/unclear (n = 179)	13,858 (18.5%)
TIVA (n = 150)	12,148 (16.2%)
Region	
Asia (n = 410)	32,619 (43.6%)
Europe (n = 296)	22,942 (30.6%)
North America (n = 80)	10,317 (13.8%)
Africa (n = 73)	5348 (7.1%)
South America (n = 10)	641 (0.9%)
Oceania (n = 8)	598 (0.8%)
Intercontinental (n = 2)	2148 (2.9%)
Unclear (n = 4)	267 (0.4)

TIVA, total intravenous anaesthesia.

* Predicted risk of postoperative pain was based on patient characteristics and the nature of surgery.

**Figure 2:**
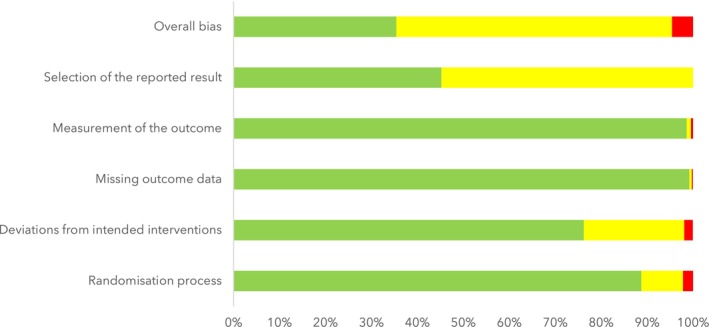
Risk of bias for multiple outcomes according to Risk of Bias 2 tool: summary of review authors' judgement about each domain, presented as percentages across included studies.

**Figure 3:**
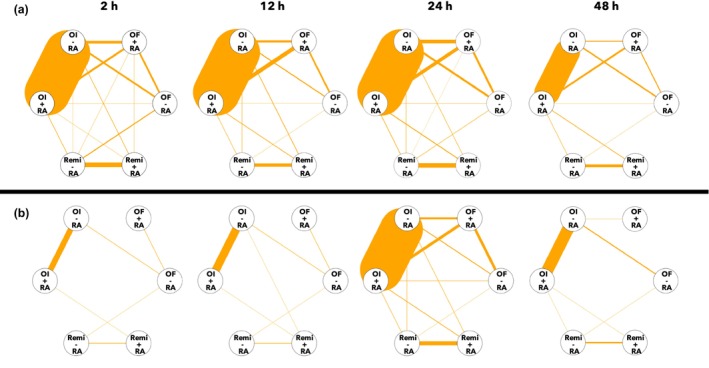
Network graphs from the meta‐analysis comparing anaesthetic techniques for (a) pain and (b) opioid consumption across different time‐points. Nodes represent the individual interventions analysed, with node size proportional to the number of patients allocated. Connecting lines indicate available direct comparisons, with line thickness reflecting the number of included trials for each comparison. OI, opioid‐inclusive; RA, regional anaesthesia; OF, opioid‐free; Remi, remifentanil; ‐, without; +, with.

Risk of bias was assessed at the outcome level using the RoB 2 tool. A total of 3272 outcome‐level judgements were made: 1159 (35.4%) were rated as low risk; 1953 (60.0%) had some concerns; and 150 (4.6%) had a high risk of overall bias (Fig. 2). The most frequent concern related to the selection of the reported result, largely due to missing prospective trial registration or accessible study protocols. A summary of the network geometry for our main outcomes is shown in Fig. 3. Full results for pairwise comparisons between interventions are in online Supporting Information Appendix S3.

**Figure 4:**
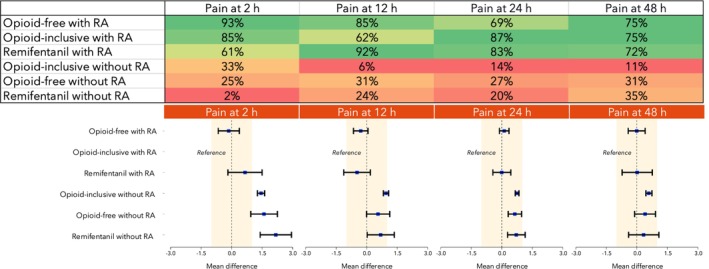
Results for postoperative pain across four time‐points. Top: heat map of the surface under the cumulative ranking curve (SUCRA) values for the six anaesthesia techniques – these values range from 0% to 100%, with higher values indicating a greater probability that the technique ranks among the most effective (i.e. lower likelihood of negative outcomes). Green, highest SUCRA values; red, lowest SUCRA values. Interventions are ordered by decreasing SUCRA values for pain at 2 h. Bottom: network meta‐analysis forest plots showing the estimated effect sizes of each technique. Blue squares, estimated mean differences; black bars, 95% credible intervals. Techniques are sorted by descending SUCRA values for pain at 2 h. RA, regional anaesthesia.

Pain outcomes at 2 h, 12 h, 24 h and 48 h were reported in 236 (15,343 patients), 301 (21,669 patients), 470 (35,700 patients) and 170 (12,954 patients) studies, respectively. Across all time‐points, techniques incorporating regional anaesthesia ranked highest, whereas those lacking regional anaesthesia were consistently associated with poorer pain control (Table 2 and Fig. 4). When regional anaesthesia was used, opioid‐inclusive and opioid‐free techniques yielded similar pain scores at all time‐points.

**Table 2:** Summary of pain outcomes at 2 h, 12 h, 24 h and 48 h across interventions.

Intervention	Pain at 2 h	Pain at 12 h	Pain at 24 h	Pain at 48 h
Opioid‐free with regional anaesthesia	‐0.14 (‐0.69–0.38) GRADE: Low Rank: 1 (1–3)	‐0.29 (‐0.65–0.06) GRADE: Low Rank: 2 (1–3)	0.12 (‐0.11–0.36) GRADE: Low Rank: 3 (1–3)	‐0.01 (‐0.43–0.41) GRADE: Low Rank: 2 (1–5)
Opioid‐inclusive with regional anaesthesia	Reference Rank: 2 (1–3)	Reference Rank: 3 (2–4)	Reference Rank: 2 (1–3)	Reference Rank: 2 (1–4)
Remifentanil as the sole opioid with regional anaesthesia	0.66 (‐0.18–1.50) GRADE: Low Rank: 3 (2–4)	‐0.47 (‐1.13–0.18) GRADE: Low Rank: 1 (1–3)	0.00 (‐0.44–0.45) GRADE: Low Rank: 2 (1–3)	0.02 (‐0.73–0.76) GRADE: Low Rank: 2 (1–5)
Opioid‐inclusive without regional anaesthesia	1.45 (1.27–1.62) GRADE: Moderate Rank: 4 (3–5)	0.95 (0.82–1.08) GRADE: Low Rank: 6 (5–6)	0.75 (0.66–0.85) GRADE: Moderate Rank: 6 (5–6)	0.59 (0.45–0.74) GRADE: Moderate Rank: 6 (6–6)
Opioid‐free without regional anaesthesia	1.59 (0.94–2.25) GRADE: Moderate Rank: 5 (4–6)	0.56 (0.00–1.13) GRADE: Low Rank: 4 (3–6)	0.64 (0.31–0.97) GRADE: Moderate Rank: 4 (4–6)	0.41 (‐0.11–0.91) GRADE: Moderate Rank: 5 (2–6)
Remifentanil as the sole opioid without regional anaesthesia	2.18 (1.40–2.95) GRADE: Low Rank: 6 (5–6)	0.69 (0.02–1.35) GRADE: Low Rank: 5 (4–6)	0.71 (0.28–1.15) GRADE: Moderate Rank: 5 (4–6)	0.33 (‐0.41–1.08) GRADE: Moderate Rank: 4 (2–6)

For each intervention and postoperative time‐point, the estimated mean difference (MD) in pain intensity (measured on a 0–10 scale) compared with the reference group (opioid‐inclusive with regional anaesthesia) is presented, along with the associated 95% credible interval (CrI), GRADE certainty of evidence and median surface under the cumulative ranking curve (SUCRA)‐based ranking (95%CrI). Interventions are listed in descending order according to their SUCRA value for pain at 2 h.

**Figure 5:**
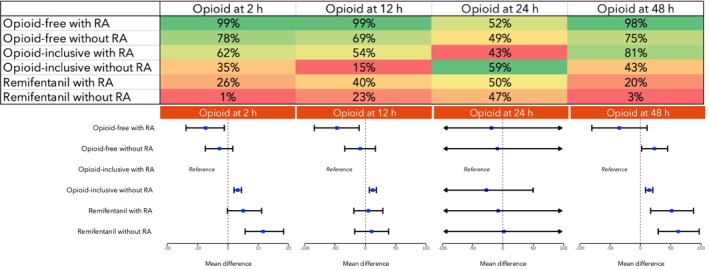
Results for opioid consumption across four time‐points. Top: heat map of the surface under the cumulative ranking curve (SUCRA) values for the six anaesthesia techniques – these values range from 0% to 100%, with higher values indicating a greater probability that the technique ranks among the most effective (i.e. lower likelihood of negative outcomes). Green, highest SUCRA values; red, lowest SUCRA values. Interventions are ordered by decreasing SUCRA values for opioid consumption within 2 h. Bottom: network meta‐analysis forest plots showing the estimated effect sizes of each technique. Blue squares, estimated mean differences; black bars, 95% credible intervals. Techniques are sorted by descending SUCRA values for opioid consumption within 2 h. RA, regional anaesthesia.

Opioid consumption was reported within 2 h, 12 h, 24 h and 48 h in 37 (2237 patients), 50 (3439 patients), 278 (18,338 patients) and 75 (5247 patients) studies, respectively. Opioid‐free anaesthesia with regional techniques consistently ranked highest for minimising opioid use, while remifentanil‐only without regional anaesthesia regimens was most likely to increase postoperative opioid requirements. At 24 h, no significant differences were observed between techniques; however, this should not be interpreted as evidence of equivalence (Fig. 5).

Duration of hospital stay was reported in 201 studies comprising 19,748 patients. Opioid‐inclusive anaesthesia without regional anaesthesia was associated with a longer duration of hospital stay compared with opioid‐inclusive anaesthesia with regional anaesthesia (mean difference (95%CrI) 15.67 (5.04–26.48) h). Time to discharge from PACU was assessed in 137 studies including 11,704 patients. All three techniques that did not include regional anaesthesia were associated with a prolonged time to PACU discharge compared with opioid‐inclusive anaesthesia combined with regional anaesthesia (online Supporting Information Appendix S3).

Postoperative nausea and vomiting were evaluated in 503 studies including 44,326 patients. Opioid‐free anaesthesia combined with regional techniques (SUCRA 99%) was most likely to be associated with the lowest odds of PONV, followed by opioid‐free anaesthesia without regional techniques (SUCRA 71%). Compared with opioid‐inclusive anaesthesia combined with regional techniques, opioid‐free anaesthesia with regional techniques reduced the odds of PONV (odds ratio (OR) (95%CrI) 0.54 (0.41–0.71)). In contrast, opioid‐inclusive anaesthesia without regional techniques (OR (95%CrI) 2.25 (1.98–2.56)) and remifentanil‐only anaesthesia without regional techniques (OR (95%CrI) 2.10 (1.35–3.12)) were associated with higher odds of PONV.

Opioid‐inclusive anaesthesia without regional techniques (OR (95%CrI) 2.57 (1.70–4.17)), as well as remifentanil‐only anaesthesia both with (OR (95%CrI) 14.80 (1.27 to ‐154.15)) and without (OR (95%CrI) 38.43 (4.59–344.66)) regional techniques, was associated with higher odds of postoperative dizziness compared with opioid‐inclusive anaesthesia with regional techniques. Both opioid‐free techniques, without (SUCRA 87%) and with regional anaesthesia (SUCRA 82%), ranked highest for minimising pruritus and reduced the odds of this complication when compared with opioid‐inclusive anaesthesia with regional techniques.

For urinary retention at 24 h, opioid‐free anaesthesia without regional anaesthesia (OR (95%CrI) 0.21 × 10^‐18^ (0.02 × 10^‐40^ to 0.56 × 10^‐6^)), as well as remifentanil‐based techniques with (OR (95%CrI) 0.57 × 10^‐11^ (0.07 × 10^‐31^ to 0.03)) and without (OR (95%CrI) 0.16 × 10^‐10^ (0.02 × 10^‐30^ to 0.06)) regional anaesthesia, significantly reduced the odds compared with opioid‐inclusive anaesthesia with regional techniques. Other complications were reported inconsistently and not summarised due to the limited number of studies or substantial heterogeneity in outcome definitions.

The network was largely consistent across outcomes and time‐points, with most node‐splitting p values > 0.05 and no evidence of global inconsistency. However, some isolated comparisons – particularly for early postoperative opioid consumption, pruritus and PONV – showed potential inconsistencies. Full results are in online Supporting Information Appendix S4. Egger's test identified evidence of funnel plot asymmetry for several outcomes, suggesting the presence of small‐study effects or publication bias. For postoperative pain, asymmetry was observed at 12 h (p = 0.036), but not at 2 h (p = 0.140), 24 h (p = 0.537) or 48 h (p = 0.820). For opioid consumption, asymmetry was evident at 2 h (p < 0.001) and 48 h (p < 0.001), but not at 12 h (p = 0.141) or 24 h (p = 0.449). Regarding recovery outcomes, asymmetry was detected for time to PACU discharge (p = 0.073) and hospital duration of stay (p < 0.001). Among postoperative complications, asymmetry was identified for PONV (p < 0.001), whereas no significant asymmetry was found for dizziness (p = 0.177), pruritus (p = 0.496) or urinary retention (p = 0.571).

An expected, increased pre‐operative risk of postoperative pain was not associated with differences in pain scores or opioid consumption at any time‐point. Similarly, the country in which the study was conducted had no significant impact on these outcomes. Dexmedetomidine (used in 85/885 studies) also did not significantly affect pain or opioid consumption at any time‐point. Subgroup analyses were additionally performed based on the risk of bias in individual studies. Results from studies judged to have low risk of bias were used to support the certainty of evidence assessments. The certainty of evidence was considered low for most interventions across most outcomes, primarily due to concerns regarding heterogeneity, imprecision and publication bias. A minority of comparisons were rated as moderate certainty.

The results for postoperative pain are summarised in Table 2. Certainty of evidence was predominantly low for opioid consumption; PACU duration of stay; hospital duration of stay; PONV; dizziness; and pruritus (online Supporting Information Appendix S5). Moderate certainty was observed consistently across all comparisons for urinary retention.

## Discussion

Our findings provide robust comparative data on the performance of opioid‐free and opioid‐inclusive anaesthetic techniques in adult patients undergoing surgery, with and without the use of regional anaesthesia. Across most outcomes, the presence of regional anaesthesia emerged as the strongest determinant of improved postoperative recovery.

For postoperative pain, techniques that incorporated regional anaesthesia consistently ranked highest at all evaluated time‐points. The influence of using opioids or not appeared minimal when regional anaesthesia was applied. These results support the well‐established role of regional anaesthesia in acute pain management and suggest that, when regional anaesthesia provides effective coverage of the expected pain trajectory with appropriate duration, intra‐operative opioid use may offer limited additional benefits for pain control [13, 23, 24, 25].

With regard to opioid consumption, opioid‐free anaesthesia combined with regional techniques ranked highest at 2 h, 12 h and 48 h postoperatively, supporting its value in minimising postoperative opioid requirements [13, 23, 24]. Notably, within 24 h, we did not find significant differences across groups, which may reflect heterogeneity in clinical scenarios or underpowered analyses rather than a true equivalence in analgesic efficacy. This is endorsed by the results at 48 h, where techniques combining regional anaesthesia with opioid‐free strategies again performed best, suggesting sustained benefits beyond the immediate postoperative period. However, while some statistically significant differences were observed, their clinical significance remains uncertain and should be interpreted in the context of all outcomes to provide a broader perspective on recovery.

Time to PACU discharge and hospital duration of stay were also shortened by regional anaesthesia, in keeping with findings from previous meta‐analyses [26, 27, 28]. All techniques without a regional component were associated with prolonged PACU stay, and opioid‐inclusive techniques without regional anaesthesia prolonged hospitalisation compared with those with regional anaesthesia. These findings align with the current emphasis on enhanced recovery after surgery and support the integration of regional techniques within peri‐operative pathways whenever feasible [29, 30, 31].

For postoperative complications, our findings suggest potential benefits from minimising intra‐operative opioid use and incorporating regional anaesthesia [29, 30, 31]. The lowest odds of PONV were observed with opioid‐free anaesthesia combined with regional techniques, followed by opioid‐free techniques without a regional component. Opioid‐inclusive techniques, particularly those using remifentanil alone or lacking regional anaesthesia, were associated with increased odds of PONV. These patterns were echoed in the analyses of pruritus and dizziness, which were more frequent in opioid‐inclusive groups. Urinary retention was the only outcome for which both opioids and regional anaesthesia appeared to contribute to increased risk. The highest odds were observed for opioid‐inclusive anaesthesia with regional techniques, while opioid‐free and remifentanil‐based strategies showed reduced odds. Nonetheless, the overall benefits of regional techniques across other outcomes likely outweigh this drawback for most cases.

These findings bring into focus the concept of opioid‐sparing anaesthesia, defined as the reduction, but not elimination, of intra‐operative opioids [7]. Although not assessed specifically in our analysis, this strategy is often considered a pragmatic compromise between opioid‐free and opioid‐inclusive approaches. Our results, however, suggest that the assumption that intra‐operative opioids are always necessary for optimal recovery may warrant reconsideration. When regional anaesthesia is feasible and effective, provides adequate coverage and is expected to last through the pain trajectory, intra‐operative opioids appear to offer little additional benefit for postoperative pain, while contributing to higher overall use and a possible increase in complications. Accordingly, recent evidence indicates that in certain patients, such as those undergoing laparoscopic bariatric surgery, opioid‐free anaesthesia may enhance recovery compared with opioid‐sparing approaches [32]. Nevertheless, clinicians should use their discretion, as certain scenarios – such as patients with chronic opioid use, opioid tolerance or anticipated high‐intensity postoperative pain – may still warrant a tailored use of intra‐operative opioids.

This study has several limitations. Although the overall direction of the evidence was consistent, the certainty of evidence was rated as low for many comparisons across outcomes, primarily due to clinical and methodological heterogeneity, imprecision and suspected publication bias. These limitations call for cautious interpretation and underscore the need for context‐sensitive clinical judgement. The heterogeneity observed may reflect differential performance of anaesthetic techniques across diverse surgical settings and patient populations, suggesting that even strategies with overall inferior performance may remain appropriate in selected scenarios. We did not analyse opioid‐inclusive techniques using opioid‐sparing approaches separately; as such, we are unable to draw specific conclusions regarding this increasingly adopted strategy. Opioid‐free anaesthesia comprises diverse techniques (e.g. dexmedetomidine, ketamine or lidocaine infusions), which may have contributed to the observed heterogeneity. As such, our findings may not apply uniformly to every specific regimen, and anaesthetists should bear this in mind when making clinical decisions. Additionally, there were insufficient studies reporting on other important recovery outcomes that are relevant to guiding anaesthetic decision‐making, such as quality of recovery scores or pain interference.

Future research might aim to clarify the role of opioid‐sparing strategies in relation to both opioid‐free and opioid‐inclusive techniques. Well‐designed trials comparing opioid‐sparing with opioid‐free anaesthesia, particularly when used alongside regional techniques, could help identify the minimal effective opioid dose needed for optimal recovery. In addition, studies tailored to specific clinical scenarios, as well as those examining patient‐centred and long‐term outcomes such as chronic pain and opioid dependence, are essential to inform best practices in peri‐operative care.

In summary, this review underscores the pivotal role of regional anaesthesia in improving postoperative outcomes and suggests that the assumed necessity of intra‐operative opioids may warrant reconsideration in many clinical contexts. When effective regional anaesthesia with adequate coverage and duration is feasible, opioid‐free strategies appear to provide a favourable balance of analgesia, reduced opioid use and fewer complications, and may represent a useful strategy in contemporary peri‐operative care.

## Supporting information


**Appendix S1.** Search strategies.

**Appendix S2.** Full list of the 885 included studies.

**Appendix S3.** League tables for all reported outcomes comparing different anaesthetic strategies.

**Appendix S4.** Assessment of inconsistency between direct and indirect evidence in the network meta‐analysis.

**Appendix S5.** Summary of network meta‐analysis findings and certainty of evidence assessments for all 14 postoperative outcomes analysed.
