# Supplementary material for: Opioid‐free vs. opioid‐inclusive anaesthesia with or without regional anaesthesia for postoperative pain: a systematic review with network meta‐analysis of randomised controlled trials
Source: Anaesthesia. 2026 Jan 5;81(5):702–12. doi: 10.1111/anae.70121 (PMC13065899; doi:10.1111/anae.70121)
Supplement: Supplementary file 1 — Appendix S1. Search strategies. [file ANAE-81-702-s003.docx]

**Appendix S1.** Search strategies.

We conducted searches on PubMed, Embase, Web of Science, Cochrane Central Register of Controlled Trials (CENTRAL), LILACS, and SciELO on 20 March 2022, with no date or language restrictions. The same search strategies were used for an updated search conducted on 15 January 2025. The complete search strategies for each database are provided below.

**PubMed**

("anesthesia, general"[MeSH] OR (general AND (anesthesia OR anaesthesia))) AND
("analgesics, opioid"[MeSH] OR opioid OR dexmedetomidine[MeSH] OR dexmedetomidine OR precedex OR MPV 1440 OR ketamine[MeSH] OR ketamin* OR 2-(2-Chlorophenyl)-2-(methylamino)cyclohexanone OR cl581 OR ketalar OR ketaset OR ketanest OR calipsol OR kalipsol OR calypsol OR lidocaine[MeSH] OR lidocaine OR 2-(Diethylamino)-N-(2,6-Dimethylphenyl)Acetamide OR 2-2EtN-2MePhAcN OR lignocaine OR xyloneural OR octocaine OR xylocaine OR dalcaine OR esmolol OR "anesthesia, epidural"[MeSH] OR peridural OR epidural OR extradural OR "anesthesia, spinal"[MeSH] OR spinal OR raqui* OR subaracnoide* OR "anesthesia, local"[MeSH] OR local OR infiltration OR "huneke neural therapy" OR "nerve block"[MeSH] OR nerve block* OR regional) AND
(bradycardia[MeSH] OR bradycardia OR bradyarrhythmia OR tachycardia[MeSH] OR tachycardia OR tachyarrhythmia OR hypertension[MeSH] OR hypertension OR hypotension[MeSH] OR hypotension OR "blood pressure" OR oxygen OR discharge OR "anesthesia recovery period"[MeSH] OR "recovery period" OR "postanesthesia care" OR PACU OR "pain, postoperative"[MeSH] OR ((postoperative OR postsurgical OR post-operative OR post-surgical) AND (pain OR analgesia OR hyperalgesia OR nociception OR adverse OR complications OR desaturation OR sedation OR depression OR consumption OR nausea OR vomiting OR PONV)))

**Embase**

('general anesthesia'/exp OR (general AND (anesthesia:ti,ab OR anaesthesia:ti,ab))) AND
('opioid analgesics' OR opioid:ti,ab OR 'dexmedetomidine'/exp OR dexmedetomidine:ti,ab OR precedex:ti,ab OR 'mpv 1440':ti,ab OR 'ketamine'/exp OR ketamin*:ti,ab OR '2-(2-chlorophenyl)-2-(methylamino)cyclohexanone':ti,ab OR cl581:ti,ab OR ketalar:ti,ab OR ketaset:ti,ab OR ketanest:ti,ab OR calipsol:ti,ab OR kalipsol:ti,ab OR calypsol:ti,ab OR 'lidocaine'/exp OR lidocaine:ti,ab OR '2-(diethylamino)-n-(2,6-dimethylphenyl)acetamide':ti,ab OR '2-2etn-2mephacn':ti,ab OR lignocaine:ti,ab OR xyloneural:ti,ab OR octocaine:ti,ab OR xylocaine:ti,ab OR dalcaine:ti,ab OR esmolol:ti,ab OR 'epidural anesthesia'/exp OR peridural:ti,ab OR epidural:ti,ab OR extradural:ti,ab OR 'spinal anesthesia'/exp OR spinal*:ti,ab OR raqui*:ti,ab OR subaracnoide*:ti,ab OR 'local anesthesia'/exp OR local:ti,ab OR infiltration:ti,ab OR 'huneke neural therapy':ti,ab OR 'nerve block'/exp OR 'nerve block*':ti,ab OR regional:ti,ab) AND
('bradycardia'/exp OR bradycardia:ti,ab OR bradyarrhythmia:ti,ab OR 'tachycardia'/exp OR tachycardia:ti,ab OR tachyarrhythmia:ti,ab OR 'hypertension'/exp OR hypertension:ti,ab OR 'hypotension'/exp OR hypotension:ti,ab OR 'blood pressure':ti,ab OR oxygen:ti,ab OR discharge:ti,ab OR 'anesthesia recovery period'/exp OR 'recovery period':ti,ab OR 'postanesthesia care':ti,ab OR pacu:ti,ab OR 'postoperative pain'/exp OR ((postoperative:ti,ab OR postsurgical:ti,ab OR 'post operative':ti,ab OR 'post surgical':ti,ab) AND (pain:ti,ab OR analgesia:ti,ab OR hyperalgesia:ti,ab OR nociception:ti,ab OR adverse:ti,ab OR complications:ti,ab OR desaturation:ti,ab OR sedation:ti,ab OR depression:ti,ab OR consumption:ti,ab OR nausea:ti,ab OR vomiting:ti,ab OR ponv:ti,ab)))

**Web of Science**

("general anesthesia" OR (general AND (anesthesia OR anaesthesia))) AND
("opioid analgesics" OR opioid* OR dexmedetomidine OR precedex OR "MPV 1440" OR ketamine OR "2-(2-Chlorophenyl)-2-(methylamino)cyclohexanone" OR cl581 OR ketalar OR ketaset OR ketanest OR calipsol OR kalipsol OR calypsol OR lidocaine OR "2-(Diethylamino)-N-(2,6-Dimethylphenyl)Acetamide" OR "2-2EtN-2MePhAcN" OR lignocaine OR xyloneural OR octocaine OR xylocaine OR dalcaine OR esmolol OR "epidural anesthesia" OR peridural OR epidural OR extradural OR "spinal anesthesia" OR spinal OR raqui* OR subaracnoide* OR "local anesthesia" OR local OR infiltration OR "huneke neural therapy" OR "nerve block" OR "nerve block*" OR regional) AND
(bradycardia OR bradyarrhythmia OR tachycardia OR tachyarrhythmia OR hypertension OR hypotension OR "blood pressure" OR oxygen OR discharge OR "anesthesia recovery period" OR "recovery period" OR "postanesthesia care" OR PACU OR "postoperative pain" OR ((postoperative OR postsurgical OR post-operative OR post-surgical) AND (pain OR analgesia OR hyperalgesia OR nociception OR adverse OR complications OR desaturation OR sedation OR depression OR consumption OR nausea OR vomiting OR PONV)))

**CENTRAL (Cochrane Central Register of Controlled Trials)**

("general anesthesia" OR (general AND (anesthesia OR anaesthesia))) AND
("opioid analgesics" OR opioid* OR dexmedetomidine OR precedex OR "MPV 1440" OR ketamine OR "2-(2-Chlorophenyl)-2-(methylamino)cyclohexanone" OR cl581 OR ketalar OR ketaset OR ketanest OR calipsol OR kalipsol OR calypsol OR lidocaine OR "2-(Diethylamino)-N-(2,6-Dimethylphenyl)Acetamide" OR "2-2EtN-2MePhAcN" OR lignocaine OR xyloneural OR octocaine OR xylocaine OR dalcaine OR esmolol OR "epidural anesthesia" OR peridural OR epidural OR extradural OR "spinal anesthesia" OR spinal OR raqui* OR subaracnoide* OR "local anesthesia" OR local OR infiltration OR "huneke neural therapy" OR "nerve block" OR "nerve block*" OR regional) AND
(bradycardia OR bradyarrhythmia OR tachycardia OR tachyarrhythmia OR hypertension OR hypotension OR "blood pressure" OR oxygen OR discharge OR "anesthesia recovery period" OR "recovery period" OR "postanesthesia care" OR PACU OR "postoperative pain" OR ((postoperative OR postsurgical OR post-operative OR post-surgical) AND (pain OR analgesia OR hyperalgesia OR nociception OR adverse OR complications OR desaturation OR sedation OR depression OR consumption OR nausea OR vomiting OR PONV)))

**LILACS**

((ti:(general anesthesia) OR ab:(general anesthesia)) OR
(ti:(anestesia geral) OR ab:(anestesia geral)) OR
(ti:(anestesia general) OR ab:(anestesia general))) AND
((ti:(opioid* OR dexmedetomidine OR ketamine OR lidocaine OR esmolol OR epidural OR spinal OR "local anesthesia" OR "nerve block") OR ab:(opioid* OR dexmedetomidine OR ketamine OR lidocaine OR esmolol OR epidural OR spinal OR "local anesthesia" OR "nerve block")) OR
(ti:(opioide* OR dexmedetomidina OR cetamina OR lidocaína OR esmolol OR peridural OR raquianestesia OR "anestesia local" OR "bloqueio nervoso") OR ab:(opioide* OR dexmedetomidina OR cetamina OR lidocaína OR esmolol OR peridural OR raquianestesia OR "anestesia local" OR "bloqueio nervoso")) OR
(ti:(opioide* OR dexmedetomidina OR ketamina OR lidocaína OR esmolol OR peridural OR "anestesia raquídea" OR "anestesia local" OR "bloqueo nervioso") OR ab:(opioide* OR dexmedetomidina OR ketamina OR lidocaína OR esmolol OR peridural OR "anestesia raquídea" OR "anestesia local" OR "bloqueo nervioso"))) AND
((ti:(bradycardia OR tachycardia OR hypertension OR hypotension OR "blood pressure" OR "postoperative pain" OR complications) OR ab:(bradycardia OR tachycardia OR hypertension OR hypotension OR "blood pressure" OR "postoperative pain" OR complications)) OR
(ti:(bradicardia OR taquicardia OR hipertensão OR hipotensão OR "pressão arterial" OR "dor pós-operatória" OR complicações) OR ab:(bradicardia OR taquicardia OR hipertensão OR hipotensão OR "pressão arterial" OR "dor pós-operatória" OR complicações)) OR
(ti:(bradicardia OR taquicardia OR hipertensión OR hipotensión OR "presión arterial" OR "dolor postoperatorio" OR complicaciones) OR ab:(bradicardia OR taquicardia OR hipertensión OR hipotensión OR "presión arterial" OR "dolor postoperatorio" OR complicaciones)))

**SciELO**

(Same search string as used in LILACS above)
