# Supplementary material for: Opioid‐free vs. opioid‐inclusive anaesthesia with or without regional anaesthesia for postoperative pain: a systematic review with network meta‐analysis of randomised controlled trials
Source: Anaesthesia. 2026 Jan 5;81(5):702–12. doi: 10.1111/anae.70121 (PMC13065899; doi:10.1111/anae.70121)
Supplement: Supplementary file 2 — Appendix S2. Full list of the 885 included studies. [file ANAE-81-702-s002.docx]

**Appendix S2.** Full list of the 885 included studies.

1. Aarab Y, Ramin S, Odonnat T et al. Pectoral Nerve Blocks for Breast Augmentation Surgery: A Randomized, Double-blind, Dual-centered Controlled Trial. *Anesthesiology* 2021; **135**: 442–53. https://doi.org/10.1097/ALN.0000000000003855.
2. Aasbø V, Ræeder JC, Grøgaard B, Røise O. No additional analgesic effect of intra‐articular morphine or bupivacaine compared with placebo after elective knee arthroscopy. *Acta Anaesthesiologica Scandinavica* 1996; **40**: 585–8. https://doi.org/10.1111/j.1399-6576.1996.tb04492.x.
3. Abdallah FW, Patel V, Madjdpour C, Cil T, Brull R. Quality of recovery scores in deep serratus anterior plane block vs. sham block in ambulatory breast cancer surgery: a randomised controlled trial. *Anaesthesia* 2021; **76**: 1190–7. https://doi.org/10.1111/anae.15373.
4. Abdallah RA, Shalaby GS, Eldin MS, Ejaimi G, Saab A. Septoplasty and Bilateral Inferior Turbinate Surgery under Local Anesthesia with Deep Sedation versus General Anesthesia, A Retrospective Randomized Comparative Control Study. *Open Access Macedonian Journal of Medical Sciences* 2023; **11**: 699–704. https://doi.org/10.3889/oamjms.2023.11738.
5. Abdelghafar EM, Abbas DN, Othman A, Zayed SBE, Shawki AH. A Prospective, Randomized Clinical Trial to Evaluate Analgesic Efficacy of Bilateral Pterygopalatine Fossa Injection in Patients Undergoing Maxillofacial Cancer Surgeries Under General Anesthesia. *Egyptian Journal of Anaesthesia* 2021; **37**: 159–66. https://doi.org/10.1080/11101849.2021.1903667.
6. Abdelghany MS, Eid GM, Belal AM, Mourad MBE. Ultrasound-guided distal glossopharyngeal nerve block for post-tonsillectomy pain relief in adults: a prospective randomized study. *Minerva anestesiologica* 2024; **90**: 500–8. https://doi.org/10.23736/S0375-9393.24.17925-4.
7. Abdelhaleem NF, Youssef EM, Hegab AS. Analgesic efficacy of inter-semispinal fascial plane block in Patients undergoing Cervical Spine Surgery through Posterior Approach: a randomized controlled trial. *Anaesthesia, critical care & pain medicine* 2023; **42**: 101213. https://doi.org/10.1016/j.accpm.2023.101213.
8. Abdelhamid BM, Belita MI, Gomaa HM, Ali MS, Mohamed AA, Hassan MM. Effect of ultrasound-guided L1/L2 paravertebral block in decreasing drug requirements during general anesthesia in patients undergoing hip surgeries; randomized controlled trial. *Egyptian Journal of Anaesthesia* 2020; **36**: 44–9. https://doi.org/10.1080/11101849.2020.1757362.
9. Abdelrady MM, Fathy GM, Abdallah MAM, Ali WN. Comparison of the effect of adding midazolam versus fentanyl to intrathecal levobupivacaine in patients undergoing cesarean section: double-blind, randomized clinical trial. *Brazilian Journal of Anesthesiology (English Edition)* 2024; **74**: 744385. https://doi.org/10.1016/j.bjane.2022.06.001.
10. Abdelrahman TN, Algharabawy WS. Opioid-free general anaesthesia for transthoracic oesophagectomy: does it improve postoperative analgesia and other recovery criteria? A prospective randomised study. *Ain-Shams Journal of Anesthesiology* 2021; **13**: 49. https://doi.org/10.1186/s42077-021-00170-6.
11. Abdullah S, Ghazali KA bin, Soh EZF, Sapuan J, Gill PS, Lim CH. A Prospective Study Comparing Wide-Awake Local Anesthesia No Tourniquet Versus General Anesthesia in Hand Fracture Fixation. *Journal of Hand Surgery Global Online* 2024; **6**: 814–7. https://doi.org/10.1016/j.jhsg.2024.06.008.
12. Aboseif A, Bedewy A, Nafei M, Hammad R, Amin S. Effect of Intraoperative Lung Recruitment and Transversus Abdominis Plane Block in Laparoscopic Bariatric Surgery on Postoperative Lung Functions: A Randomized Controlled Study. *Anesthesiology and Pain Medicine* 2023; **13**. https://doi.org/10.5812/aapm-128440.
13. Abouammoh MA, Abdelhalim AA, Mohamed EA, Elzoughari I, Mustafa M, Al-Zahrani TA. Subtenon block combined with general anesthesia for vitreoretinal surgery improves postoperative analgesia in adult: a randomized controlled trial. *Journal of Clinical Anesthesia* 2016; **30**: 78–86. https://doi.org/10.1016/j.jclinane.2015.11.013.
14. Abougabal A, Hamed E, Mannaa A, Nabil N, Badry M. Efficacy of ultrasound guided rectus sheath block on the postoperative quality of recovery in laparotomy surgeries; a randomized control trial. *Anaesthesia, Pain & Intensive Care* 2024; **28**: 489–94. https://doi.org/10.35975/apic.v28i3.2456.
15. Abramov Y, Sand PK, Gandhi S et al. The Effect of Preemptive Pudendal Nerve Blockade on Pain After Transvaginal Pelvic Reconstructive Surgery. *Obstetrics & Gynecology* 2005; **106**: 782–8. https://doi.org/10.1097/01.AOG.0000165275.39905.0d.
16. Abubaker AK, Al-Qudah MA. The Role of Endoscopic Sphenopalatine Ganglion Block on Nausea and Vomiting After Sinus Surgery. *American Journal of Rhinology & Allergy* 2018; **32**: 369–73. https://doi.org/10.1177/1945892418782235.
17. Acharya R, Sriramka B, Koushik P. Comparison of dexmedetomidine alone with dexmedetomidine and fentanyl during awake fiberoptic intubation in patients with difficult airway: a randomized clinical trial. *Journal of Dental Anesthesia and Pain Medicine* 2022; **22**: 349-56. https://doi.org/10.17245/jdapm.2022.22.5.349.
18. Afandy ME, Elghafar MSA, Shoukr TG, Mourad MBE. Efficacy of ultrasound-guided suprazygomatic maxillary nerve block on emergence agitation and postoperative analgesia after septorhinoplasty: A prospective randomized trial. *Journal of Anaesthesiology Clinical Pharmacology* 2024; **40**: 679–85. https://doi.org/10.4103/joacp.joacp_256_23.
19. Agarwal A, Batra R, Chhabra A, Subramaniam R, Misra M. The evaluation of efficacy and safety of paravertebral block for perioperative analgesia in patients undergoing laparoscopic cholecystectomy. *Saudi Journal of Anaesthesia* 2012; **6**: 344-49 https://doi.org/10.4103/1658-354X.105860.
20. Agarwal A, Pandey R, Dhiraaj S et al. The Effect of Epidural Bupivacaine on Induction and Maintenance Doses of Propofol (Evaluated by Bispectral Index) and Maintenance Doses of Fentanyl and Vecuronium. *Anesthesia & Analgesia* 2004; **99**: 1684–8. https://doi.org/10.1213/01.ANE.0000136422.70531.5A.
21. Aghamelu O, Buggy P, Smith G, Inzitari R, Wall T, Buggy DJ. Serum NETosis expression and recurrence risk after regional or volatile anaesthesia during breast cancer surgery: A pilot, prospective, randomised single‐blind clinical trial. *Acta Anaesthesiologica Scandinavica* 2021; **65**: 313–9. https://doi.org/10.1111/aas.13745.
22. Aglio LS, Abd-El-Barr MM, Orhurhu V et al. Preemptive analgesia for postoperative pain relief in thoracolumbosacral spine operations: a double-blind, placebo-controlled randomized trial. *Journal of Neurosurgery: Spine* 2018; **29**: 647–53. https://doi.org/10.3171/2018.5.SPINE171380.
23. Agnew NM, Tan NH, Scawn NDA, Pennefather SH, Russell GN. Choice of opioid supplementation for day-case rigid bronchoscopy:a randomized placebo-controlled comparison of a bolus of remifentanil and alfentanil. *Journal of Cardiothoracic and Vascular Anesthesia* 2003; **17**: 336–40. https://doi.org/10.1016/S1053-0770(03)00049-1.
24. Agren K, Engquist S, Danneman A, Feychting B. Local versus general anaesthesia in tonsillectomy. *Clinical otolaryngology and allied sciences* 1989; **14**: 97–100. https://doi.org/10.1111/j.1365-2273.1989.tb00343.x.
25. Aguilar JL, Rincón R, Domingo V, Espachs P, Preciado MJ, Vidal F. Absence of an early pre-emptive effect after thoracic extradural bupivacaine in thoracic surgery. *British Journal of Anaesthesia* 1996; **76**: 72–6. https://doi.org/10.1093/bja/76.1.72.
26. Ahiskalioglu A, Yayik AM, Ahiskalioglu EO et al. Ultrasound-guided bilateral superficial cervical block and preemptive single-dose oral tizanidine for post-thyroidectomy pain: a randomized-controlled double-blind study. *Journal of Anesthesia* 2018; **32**: 219–26. https://doi.org/10.1007/s00540-018-2468-x.
27. Ahiskalioglu A, Yayik AM, Demir U et al. Preemptive Analgesic Efficacy of the Ultrasound-Guided Bilateral Superficial Serratus Plane Block on Postoperative Pain in Breast Reduction Surgery: A Prospective Randomized Controlled Study. *Aesthetic Plastic Surgery* 2020; **44**: 37–44. https://doi.org/10.1007/s00266-019-01542-y.
28. Ahlberg H, Wallgren D, Hultin M, Myrberg T, Johansson J. Less use of rescue morphine when a combined PSP/IPP-block is used for postoperative analgesia in breast cancer surgery. *European Journal of Anaesthesiology* 2023; **40**: 636–42. https://doi.org/10.1097/EJA.0000000000001795.
29. Ahmed A, Saad D, Youness AR. Superior laryngeal nerve block as an adjuvant to General Anesthesia during endoscopic laryngeal surgeries. *Egyptian Journal of Anaesthesia* 2015; **31**: 167–74. https://doi.org/10.1016/j.egja.2015.01.006.
30. Ahmed MHM, Abu-Zaid MEH. Role of Intraoperative Endoscopic Sphenopalatine Ganglion Block in Sinonasal Surgery. *Journal of Medical Sciences* 2007; **7**: 1297–303. https://doi.org/10.3923/jms.2007.1297.1303.
31. Ahmed Z, Samad K, Ullah H. Role of intercostal nerve block in reducing postoperative pain following video-assisted thoracoscopy: A randomized controlled trial. *Saudi Journal of Anaesthesia* 2017; **11**: 54-7. https://doi.org/10.4103/1658-354X.197342.
32. Ak K, Gursoy S, Duger C et al. Thoracic Paravertebral Block for Postoperative Pain Management in Percutaneous Nephrolithotomy Patients: A Randomized Controlled Clinical Trial. *Medical Principles and Practice* 2013; **22**: 229–33. https://doi.org/10.1159/000345381.
33. Akcaboy EY, Akcaboy ZN, Gogus N. Comparison of paravertebral block versus fast-track general anesthesia via laryngeal mask airway in outpatient inguinal herniorrhaphy. *Journal of Anesthesia* 2010; **24**: 687–93. https://doi.org/10.1007/s00540-010-0966-6.
34. Akkaya T, Ersan O, Ozkan D et al. Saphenous nerve block is an effective regional technique for post‐menisectomy pain. *Knee Surgery, Sports Traumatology, Arthroscopy* 2008; **16**: 855–8. <https://doi.org/10.1007/s00167-008-0572-4>.
35. Aksu G, Gurkan Y, Kus A, Toker K, Solak Z. Evaluation of analgesic effect of pectoral block in patients undergoing breast cancer surgery. *Regional Anesthesia & Pain Medicine* 2014; **39**: E203-4. https://doi.org/10.1097/AAP.0000000000000142.
36. Aksu R, Patmano G, Biçer C, Emek E, Çoruh AE. Eficácia de bupivacaína e associação com dexmedetomidina em bloqueio do plano transverso abdominal guiado por ultrassom na dor após cirurgia abdominal. *Brazilian Journal of Anesthesiology* 2018; **68**: 49–56. https://doi.org/10.1016/j.bjan.2017.04.021.
37. Al-Kaisy M, McGuire G, Chan V. Analgesic effect of interscalene block using low-dose bupivacaine for outpatient arthroscopic shoulder surgery. *Reg Anesth Pain Med* 1998; **5**: 469–73.
38. Al‐Qudah M. Endoscopic sphenopalatine ganglion blockade efficacy in pain control after endoscopic sinus surgery. *International Forum of Allergy & Rhinology* 2016; **6**: 334–8. https://doi.org/10.1002/alr.21644.
39. Alagol A, Calpur OU, Usar PS, Turan N, Pamukcu Z. Intraarticular analgesia after arthroscopic knee surgery: comparison of neostigmine, clonidine, tenoxicam, morphine and bupivacaine. *Knee Surgery, Sports Traumatology, Arthroscopy* 2005; **13**: 658–63. https://doi.org/10.1007/s00167-004-0612-7.
40. Albrecht E, Kirkham KR, Endersby RVW et al. Ultrasound-Guided Transversus Abdominis Plane (TAP) Block for Laparoscopic Gastric-Bypass Surgery:a Prospective Randomized Controlled Double-Blinded Trial. *Obesity Surgery* 2013; **23**: 1309–14. https://doi.org/10.1007/s11695-013-0958-3.
41. Alessandri F, Lijoi D, Mistrangelo E, Nicoletti A, Ragni N. Effect of presurgical local infiltration of levobupivacaine in the surgical field on postsurgical wound pain in laparoscopic gynecological surgery. *Acta Obstetricia et Gynecologica Scandinavica* 2006; **85**: 844–9. https://doi.org/10.1080/00016340500494846.
42. Ali AR, Sakr SA, Rahman ASMA. Bilateral sphenopalatine ganglion block as adjuvant to general anaesthesia during endoscopic trans-nasal resection of pituitary adenoma. *Egyptian Journal of Anaesthesia* 2010; **26**: 273–80. https://doi.org/10.1016/j.egja.2010.05.002.
43. Allam S, Lakshmi DA. Comparison of the Effects of Midazolam (1mg) and Fentanyl (25 Mcg) as Additives to Intrathecal 3ml of 0.5% Bupivacaine (15mg) For Spinal Anaesthesia. *Eur. J. Mol. Clin. Med.* 2022; **9**: 9921–36.
44. Altiparmak B, Toker MK, Uysal Aİ, Kuşçu Y, Demirbilek SG. [Efficacy of ultrasound-guided erector spinae plane block for analgesia after laparoscopic cholecystectomy: a randomized controlled trial]. *Brazilian journal of anesthesiology (Elsevier)* 2019; **69**: 561–8. https://doi.org/10.1016/j.bjan.2019.09.001.
45. Altıparmak B, Toker MK, Uysal AI, Dere Ö, Uğur B. Evaluation of ultrasound-guided rhomboid intercostal nerve block for postoperative analgesia in breast cancer surgery: a prospective, randomized controlled trial. *Regional Anesthesia & Pain Medicine* 2020; **45**: 277–82. https://doi.org/10.1136/rapm-2019-101114.
46. Amato PE, Coleman JR, Dobrzanski TP et al. Pericapsular nerve group (PENG) block for hip arthroscopy: a randomized, double-blinded, placebo-controlled trial. *Regional Anesthesia & Pain Medicine* 2022; **47**: 728–32. https://doi.org/10.1136/rapm-2022-103907.
47. Amin OAI, Salah HE. The effect of general or spinal anaesthesia on pro- and anti-inflammatory intracellular cytokines in patients undergoing appendicectomy using flowcytometric method. *Egyptian Journal of Anaesthesia* 2011; **27**: 121–5. https://doi.org/10.1016/j.egja.2011.04.005.
48. Amin SM, Hasanin A, ElSayed OS et al. Comparison of the hemodynamic effects of opioid-based versus lidocaine-based induction of anesthesia with propofol in older adults: a randomized controlled trial. *Anaesthesia Critical Care & Pain Medicine* 2023; **42**: 101225. https://doi.org/10.1016/j.accpm.2023.101225.
49. Ammar MA, Taeimah M. Evaluation of thoracolumbar interfascial plane block for postoperative analgesia after herniated lumbar disc surgery: A randomized clinical trial. *Saudi journal of anaesthesia* 2018; **12**: 559–64. https://doi.org/10.4103/sja.SJA_177_18.
50. Amr SA, Othman AH, Ahmed EH, Naeem RG, Kamal SM. Comparison between ultrasound guided erector spinae plane block and paravertebral block on acute and chronic post mastectomy pain after modified radical mastectomy: randomized controlled trial. *BMC Anesthesiology* 2024; **24**: 420. https://doi.org/10.1186/s12871-024-02810-4.
51. An G, Wang G, Zhao B et al. Opioid-free anesthesia compared to opioid anesthesia for laparoscopic radical colectomy with pain threshold index monitoring: a randomized controlled study. *BMC Anesthesiology* 2022; **22**: 241. https://doi.org/10.1186/s12871-022-01747-w.
52. An G, Zhang Y, Chen N, Fu J, Zhao B, Zhao X. Opioid-free anesthesia compared to opioid anesthesia for lung cancer patients undergoing video-assisted thoracoscopic surgery: A randomized controlled study. *PLOS ONE* 2021; **16**: e0257279. https://doi.org/10.1371/journal.pone.0257279.
53. Anand S, Nirala DK, Prasad S. Spinal versus General Anesthesia for Transabdominal Preperitoneal (TAPP) Repair of Inguinal Hernia: Interim Analysis of a Controlled Randomized Trial. *International Journal of Pharmaceutical and Clinical Research 2024;* **16**: 2507-10.
54. Andrabi WI, Khawaja MA, Fatima K et al. The Impact of Preemptive Perianal Anaesthetic Block in Open Haemorrhoidectomy – a randomised controlled trial. *Pakistan Journal of Medical and Health Sciences* 2021; **15**: 2165–7. https://doi.org/10.53350/pjmhs211592165.
55. Andrieu G, Amrouni H, Robin E et al. Analgesic efficacy of bilateral superficial cervical plexus block administered before thyroid surgery under general anaesthesia. *British Journal of Anaesthesia* 2007; **99**: 561–6. https://doi.org/10.1093/bja/aem230.
56. Angral R, Lachala S, Gupta S. Post operative analgesic technique in laparoscopic cholecystectomy: Comparison of local instillation with bupivacaine vs intravenous butorphanol vs intercostal nerve block with bupivacaine. *Sri Lankan Journal of Anaesthesiology* 2013; **21**: 9-13. https://doi.org/10.4038/slja.v21i1.4082.
57. Anis S, Moaty NAE, Youssef A, Ramzy R, Hassan R. Lumbar plexus block as a method of postoperative analgesia after hip surgery. *Egyptian Journal of Anaesthesia* 2011; **27**: 127–33. https://doi.org/10.1016/j.egja.2011.02.001.
58. Apan A, Apan ÖC, Köse EA. Segmental epidural anesthesia for percutaneous kyphoplasty:comparison with general anesthesia. *Turkish journal of medical sciences* 2016; **46**: 1801–7. https://doi.org/10.3906/sag-1507-90.
59. Araco F, Gravante G, Sorge R et al. Sedation with local versus general anesthesia for the tension‐free vaginal tape Secur hammock procedure. *International Journal of Gynecology & Obstetrics* 2011; **113**: 108–11. https://doi.org/10.1016/j.ijgo.2010.11.012.
60. Arun TC, Karim HMR, Singha SK, Biswal DK. A Comparison of Analgesic and Recovery Profiles of Ketamine, Lignocaine, and Dexmedetomidine (KeLiDex) Versus Fentanyl-Based Anesthesia in Laparoscopic Nephrectomies: A Randomized, Single-Blind, Pilot Study. *Cureus* 2024; **16**: e63380. https://doi.org/10.7759/cureus.63380.
61. Arunakul P. General anesthesia with thoracic paravertebral block for modified radical mastectomy. *J Med Assoc Thai.* 2020; **93**: 149–53.
62. Ashour MR, AbdelLatif SA, Altaher WAM, ElSayed HH, Koraitim AF, Alhadidy MA. A comparative study between Thoracic Epidural Anesthesia in non-intubated video-assisted thoracoscopes and the conventional general anesthesia with one lung ventilation. *Egyptian Journal of Anaesthesia* 2022; **38**: 284–90. https://doi.org/10.1080/11101849.2022.2077050.
63. Atalay C, Dağli C, Bayram E et al. Effects of three different methods of anesthesia on the release of brain natriuretic peptid in patients with cardiac risk undergoing lower extremity surgery. *Turkish Journal of Medical Sciences* 2010; **40**. https://doi.org/10.3906/sag-0907-91.
64. Atanassoff PG, Alon E, Weiss BM. Intercostal nerve block for lumpectomy: Superior postoperative pain relief with bupivacaine. *Journal of Clinical Anesthesia* 1994; **6**: 47–51. https://doi.org/10.1016/0952-8180(94)90118-X.
65. Athar M, Parveen S, Yadav M et al. A Randomized Double-Blind Controlled Trial to Assess the Efficacy of Ultrasound-Guided Erector Spinae Plane Block in Cardiac Surgery. *Journal of Cardiothoracic and Vascular Anesthesia* 2021; **35**: 3574–80. https://doi.org/10.1053/j.jvca.2021.03.009.
66. Attari MA, Mirhosseini SA, Honarmand A, Safavi MR. Spinal anesthesia versus general anesthesia for elective lumbar spine surgery: A randomized clinical trial. *Journal of research in medical sciences : the official journal of Isfahan University of Medical Sciences* 2011; **16**: 524–9.
67. Avci O, Gundogdu O, Balci F, Tekcan MN. Effects of Modified Thoracoabdominal Nerve Block Through Perichondrial Approach on Postoperative Pain and Analgaesic Consumption in Patients Undergoing Laparoscopic Cholecystectomy. *Journal of the College of Physicians and Surgeons–Pakistan*2024; **34**: 5–10. https://doi.org/10.29271/jcpsp.2024.01.05.
68. Avci O, Kol IO, Gundogdu O, Oztemur Z, Ersan I. Effect of adding dexmedetomidine to intra-articular levobupivacaine on postoperative pain following arthroscopic meniscus surgery: A prospective, double-blind, randomized, placebo- controlled, clinical trial. *Acta Orthopaedica et Traumatologica Turcica* 2021; **55**: 316–20. https://doi.org/10.5152/j.aott.2021.20212.
69. Avis G, Gricourt Y, Vialatte PB et al. Analgesic efficacy of erector spinae plane blocks for lumbar spine surgery: a randomized double-blind controlled clinical trial. *Regional Anesthesia & Pain Medicine* 2022; **47**: 610–6. https://doi.org/10.1136/rapm-2022-103737.
70. Aydin G, Aydin O. The Efficacy of Ultrasound-Guided Paravertebral Block in Laparoscopic Cholecystectomy. *Medicina* 2018; **54**: 75. https://doi.org/10.3390/medicina54050075.
71. Bäcklund M, Toivonen L, Tuominen M, Pere P, Lindgren L. Changes in heart rate variability in elderly patients undergoing major noncardiac surgery under spinal or general anesthesia. *Regional anesthesia and pain medicine* 1999; **24**: 386–92. https://doi.org/10.1016/s1098-7339(99)90002-0.
72. Badiea MAMA, Moustafa ZAA, Alem AAAE, Mohamed WAM, Elbeialy MAK. General anesthesia versus ultrasound-guided axillary block for ambulatory hand surgery: randomized prospective study. *Ain-Shams Journal of Anesthesiology* 2022; **14**: 47. https://doi.org/10.1186/s42077-022-00243-0.
73. Baenziger B, Nadi N, Doerig R et al. Regional Versus General Anesthesia: Effect of Anesthetic Techniques on Clinical Outcome in Lumbar Spine Surgery: A Prospective Randomized Controlled Trial. *Journal of Neurosurgical Anesthesiology* 2020; **32**: 29–35. https://doi.org/10.1097/ANA.0000000000000555.
74. Bagul A, Taha R, Metcalfe MS, Brook NR, Nicholson ML. Pre-Incision Infiltration of Local Anesthetic Reduces Postoperative Pain with No Effects on Bruising and Wound Cosmesis After Thyroid Surgery. *Thyroid* 2005; **15**: 1245–8. https://doi.org/10.1089/thy.2005.15.1245.
75. Bain GI, Rudkin G, Comley AS, Heptinstall RJ, Chittleborough M. Digitally assisted acromioplasty. *Arthroscopy: The Journal of Arthroscopic & Related Surgery* 2001; **17**: 44–9. https://doi.org/10.1053/jars.2001.19665.
76. Bajpai S, Kumar KS, Patibandla S, Giridhar CM. Ultrasound-guided continuous erector spinae plane block for perioperative opioid sparing analgesia in breast cancer surgery: A randomized controlled trial. *Saudi Journal of Anaesthesia* 2023; **17**: 327–33. https://doi.org/10.4103/sja.sja_760_22.
77. Bajwa SJS, Kaur J, Singh A. A comparative evaluation of epidural and general anaesthetic technique for renal surgeries: A randomised prospective study. *Indian journal of anaesthesia* 2014; **58**: 410–5. https://doi.org/10.4103/0019-5049.138975.
78. Bakan M, Umutoglu T, Topuz U et al. Opioid-free total intravenous anesthesia with propofol, dexmedetomidine and lidocaine infusions for laparoscopic cholecystectomy: a prospective, randomized, double-blinded study. *Brazilian Journal of Anesthesiology (English Edition)* 2015; **65**: 191–9. https://doi.org/10.1016/j.bjane.2014.05.001.
79. Bakeer AH, Kamel KM, Galil ASA, Ghoneim AA, Soud AHA, Hassan ME. Modified Pectoral Nerve Block versus Serratus Block for Analgesia Following Modified Radical Mastectomy: A Randomized Controlled Trial</p>. *Journal of Pain Research* 2020; **13**: 1769–75. https://doi.org/10.2147/JPR.S252539.
80. Bakhtiary F, Therapidis P, Dzemali O et al. Impact of high thoracic epidural anesthesia on incidence of perioperative atrial fibrillation in off-pump coronary bypass grafting: A prospective randomized study. *The Journal of Thoracic and Cardiovascular Surgery* 2007; **134**: 460–4. https://doi.org/10.1016/j.jtcvs.2007.03.043.
81. Bakri MH, Ismail EA, Ghanem G, Shokry M. Spinal versus general anesthesia for Cesarean section in patients with sickle cell anemia. *Korean Journal of Anesthesiology* 2015; **68**: 469. https://doi.org/10.4097/kjae.2015.68.5.469.
82. Bakshi SG, Mapari A, Shylasree TS. REctus Sheath block for postoperative analgesia in gynecological ONcology Surgery (RESONS): a randomized-controlled trial. *Canadian Journal of Anesthesia/Journal canadien d’anesthésie* 2016; **63**: 1335–44. https://doi.org/10.1007/s12630-016-0732-9.
83. Bala R, Gehlaut P, Mittal K, Singh I. Ropivacaine instillation through subgaleal drain: A novel approach for acute post-craniotomy pain. *Indian Journal of Anaesthesia* 2022; **66**: 498–504. https://doi.org/10.4103/ija.ija_646_21.
84. Balan C, Tomescu DR, Valeanu L et al. Nociception Level Index-Directed Erector Spinae Plane Block in Open Heart Surgery: A Randomized Controlled Clinical Trial. *Medicina* 2022; **58**: 1462. https://doi.org/10.3390/medicina58101462.
85. Balaramareddy P. Ketamine And Propofol Vs Fentanyl And Propofol In Total Intravenous Anaesthesia For Short Surgical Procedures: a Comparative Study In Tertiary Care Centre In South India. *Journal of Cardiovascular Disease Research* 2023; **14**: 931-9.
86. Balasubramanian K, Botta BV, Chandhinie, Soundarya G. Comparison of Dexmedetomidine and Fentanyl Added to Levobupivacaine in USG-guided Axillary Block for Upper Limb Surgeries: A Randomised Double-blinded Controlled Study. *Journal of Clinical and Diagnostic Research* 2024; **18**: 1-5. https://doi.org/10.7860/JCDR/2024/65358.19218.
87. Bameshki A, Jahanbakhsh S. Abdominal Pain after Cataract Surgery with Remifentanil Based Anesthesia. *Iranian Journal of Pharmaceutical Research* 2009; **8**: 47–51.
88. Bamigboye AA, Justus HG. Ropivacaine abdominal wound infiltration and peritoneal spraying at cesarean delivery for preemptive analgesia. *International Journal of Gynecology & Obstetrics* 2008; **102**: 160–4. https://doi.org/10.1016/j.ijgo.2008.03.019.
89. Ban M, Choi YS, Koo B-N. Analgesic Effect of Intrathecal Morphine Combined with Low-Dose Bupivacaine on Postoperative Analgesia after Liver Resection: A Randomized Controlled Study. *Journal of Personalized Medicine* 2022; **12**: 211. https://doi.org/10.3390/jpm12020211.
90. Bang YJ, Lee EK, Kim CS et al. The Effect of Intrathecal Morphine on Postoperative Opioid Consumption in Patients Undergoing Abdominal Surgery for Gynecologic Malignancy: A Randomized Sham-Controlled Trial. *Anesthesia & Analgesia* 2023; **137**: 525–33. https://doi.org/10.1213/ANE.0000000000006358.
91. Bao R, Zhang W, Zha Y et al. Effects of opioid-free anaesthesia compared with balanced general anaesthesia on nausea and vomiting after video-assisted thoracoscopic surgery: a single-centre randomised controlled trial. *BMJ Open* 2024; **14**: e079544. https://doi.org/10.1136/bmjopen-2023-079544.
92. Bao Y, Xiong J, Wang H, Zhang Y, Zhong Q, Wang G. Ultrasound-Guided Block of the Internal Branch of the Superior Laryngeal Nerve Reduces Postoperative Sore Throat Caused by Suspension Laryngoscopic Surgery: A Prospective Randomized Trial. *Frontiers in Surgery* 2022; **9**. https://doi.org/10.3389/fsurg.2022.829811.
93. Barakat H, Gholmieh L, Nader JA et al. Opioid-free versus opioid-based anesthesia in laparoscopic sleeve gastrectomy: a single-center, randomized, controlled trial. *Perioperative Medicine* 2025; **14**: 16. https://doi.org/10.1186/s13741-024-00486-5.
94. Barba M, Cola A, Vicari DD, Melocchi T, Gili MA, Frigerio M. Enhanced recovery after surgery (ERAS) in prolapse repair: A prospective study on pre‐emptive uterosacral/cervical block. *International Journal of Gynecology & Obstetrics* 2024; **166**: 1240–6. https://doi.org/10.1002/ijgo.15483.
95. Barbosa RAG, Silva CD da, Torniziello MYT, Cerri LM de O, Carmona MJC, Malbouisson LMS. A Comparative Study among Three Techniques of General Anesthesia for Ultrasound-Guided Transrectal Prostate Biopsy. *Brazilian Journal of Anesthesiology* 2010; **60**: 457–65. https://doi.org/10.1016/S0034-7094(10)70057-X.
96. Barker JP, Vafids GC, Hall GM. Postoperative morbidity following cataract surgery. *Anaesthesia* 1996; **51**: 435–7. https://doi.org/10.1111/j.1365-2044.1996.tb07786.x.
97. Barrington MJ, Kluger R, Watson R, Scott DA, Harris KJ. Epidural Anesthesia for Coronary Artery Bypass Surgery Compared with General Anesthesia Alone Does Not Reduce Biochemical Markers of Myocardial Damage. *Anesthesia & Analgesia* 2005; **100**: 921–8. https://doi.org/10.1213/01.ANE.0000146437.88485.47.
98. Barsa M. EP049 Erector spine plane block with general anaesthesia compared with general anaesthesia without regional component for spine surgery: prospective randomized controlled trial. *ePoster Session 2 – Station 3*. BMJ Publishing Group Ltd, 2023; **48**: A67.1-A67. https://doi.org/ 10.1136/rapm-2023-ESRA.111.
99. Basaran B, Basaran A, Kozanhan B, Kasdogan E, Eryilmaz MA, Ozmen S. Analgesia and Respiratory Function after Laparoscopic Cholecystectomy in Patients receiving Ultrasound-Guided Bilateral Oblique Subcostal Transversus Abdominis Plane Block: A Randomized Double-Blind Study. *Medical Science Monitor* 2015; **21**: 1304–12. https://doi.org/10.12659/MSM.893593.
100. Bashandy GMN, Abbas DN. Pectoral Nerves I and II Blocks in Multimodal Analgesia for Breast Cancer Surgery. *Regional Anesthesia and Pain Medicine* 2015; **40**: 68–74. https://doi.org/10.1097/AAP.0000000000000163.
101. Baytar Ç, Aktaş B, Aydin BG, Pişkin Ö, Çakmak GK, Ayoğlu H. The effects of ultrasound-guided serratus anterior plane block on intraoperative opioid consumption and hemodynamic stability during breast surgery: A randomized controlled study. *Medicine* 2022; **101**: e30290. https://doi.org/10.1097/MD.0000000000030290.
102. Beers RA, Calimlim JR, Uddoh E, Esposito BF, Camporesi EM. A Comparison of the Cost-Effectiveness of Remifentanil Versus Fentanyl as an Adjuvant to General Anesthesia for Outpatient Gynecologic Surgery. *Anesthesia & Analgesia* 2000; **91**: 1420–5. https://doi.org/10.1097/00000539-200012000-00022.
103. Beleña JM, Núñez M, Vidal A, Anta D. Randomized double-blind comparison of remifentanil and alfentanil in patients undergoing laparoscopic cholecystectomy using total intravenous anesthesia. *Journal of anaesthesiology, clinical pharmacology* 2016; **32**: 487–91. https://doi.org/10.4103/0970-9185.173368.
104. Beloeil H, Garot M, Lebuffe G et al. Balanced Opioid-free Anesthesia with Dexmedetomidine versus Balanced Anesthesia with Remifentanil for Major or Intermediate Noncardiac Surgery. *Anesthesiology* 2021; **134**: 541–51. https://doi.org/10.1097/ALN.0000000000003725.
105. Bergman L, Bäckmark I, Ones H et al. Preoperative Sub–Tenon’s Capsule Injection of Ropivacaine in Conjunction with General Anesthesia in Retinal Detachment Surgery. *Ophthalmology* 2007; **114**: 2055–60. https://doi.org/10.1016/j.ophtha.2006.12.031.
106. Bessa SS, El-Sayes IA, El-Saiedi MK, Abdel-Baki NA, Abdel-Maksoud MM. Laparoscopic Cholecystectomy Under Spinal Versus General Anesthesia: A Prospective, Randomized Study. *Journal of Laparoendoscopic & Advanced Surgical Techniques* 2010; **20**: 515–20. https://doi.org/10.1089/lap.2010.0041.
107. Bessa SS, Katri KM, Abdel-Salam WN, El-Kayal E-SA, Tawfik TA. Spinal Versus General Anesthesia for Day-Case Laparoscopic Cholecystectomy: A Prospective Randomized Study. *Journal of Laparoendoscopic & Advanced Surgical Techniques* 2012; **22**: 550–5. https://doi.org/10.1089/lap.2012.0110.
108. Beyls F, Martins ME, Teunkens A, Velde MVD. Additional analgesia after ambulatory unilateral laparoscopic inguinal hernia repair with transversus abdominis plane (TAP) block: A randomized double-blind controlled trial. *European Journal of Pain Supplements* 2011; **5**: 507–8. https://doi.org/10.1016/j.eujps.2011.08.025.
109. Beyoğlu ÇA, Özdilek A, Erbabacan E et al. Evaluation of the effects of subcostal transversus abdominis plane block on acute and subacute pain development following inguinal herniography: Randomized clinical study. *Agri : Agri (Algoloji) Dernegi’nin Yayin organidir = The journal of the Turkish Society of Algology* 2018; **30**: 123–9. https://doi.org/10.5505/agri.2018.49344.
110. Bhan S, Mishra S, Gupta N et al. A Prospective Randomised Study to Assess the Analgesic Efficacy of Serratus Anterior Plane (SAP) Block for Modified Radical Mastectomy Under General Anaesthesia. *Turkish Journal of Anaesthesiology and Reanimation* 2021; **49**: 124–9. https://doi.org/10.5152/TJAR.2020.13.
111. Bhardwaj S, Garg K, Devgan S. Comparison of opioid-based and opioid-free TIVA for laparoscopic urological procedures in obese patients. *Journal of anaesthesiology, clinical pharmacology* 2019; **35**: 481–6. https://doi.org/10.4103/joacp.JOACP_382_18.
112. Bharti N, Kumar P, Bala I, Gupta V. The Efficacy of a Novel Approach to Transversus Abdominis Plane Block for Postoperative Analgesia After Colorectal Surgery. *Anesthesia & Analgesia* 2011; **112**: 1504–8. https://doi.org/10.1213/ANE.0b013e3182159bf8.
113. Bhatia N, Arora S, Jyotsna W, Kaur G. Comparison of posterior and subcostal approaches to ultrasound-guided transverse abdominis plane block for postoperative analgesia in laparoscopic cholecystectomy. *Journal of Clinical Anesthesia* 2014; **26**: 294–9. https://doi.org/10.1016/j.jclinane.2013.11.023.
114. Bhattacharjee S, Ray M, Ghose T, Maitra S, Layek A. Analgesic efficacy of transversus abdominis plane block in providing effective perioperative analgesia in patients undergoing total abdominal hysterectomy: A randomized controlled trial. *Journal of Anaesthesiology Clinical Pharmacology* 2014; **30**: 391-6. https://doi.org/10.4103/0970-9185.137274.
115. Bhuvaneswari V, Wig J, Mathew P, Singh G. Post-operative pain and analgesic requirements after paravertebral block for mastectomy: A randomized controlled trial of different concentrations of bupivacaine and fentanyl. *Indian Journal of Anaesthesia* 2012; **56**: 34. https://doi.org/10.4103/0019-5049.93341.
116. Biboulet P, Morau D, Aubas P, Bringuier-Branchereau S, Capdevila X. Postoperative analgesia after total-hip arthroplasty: Comparison of intravenous patient-controlled analgesia with morphine and single injection of femoral nerve or psoas compartment block. a prospective, randomized, double-blind study. *Regional anesthesia and pain medicine* 2004; **29**: 102–9. https://doi.org/10.1016/j.rapm.2003.11.006.
117. Bielka K, Kuchyn I, Tokar I, Artemenko V, Kashchii U. Psoas compartment block efficacy and safety for perioperative analgesia in the elderly with proximal femur fractures: a randomized controlled study. *BMC Anesthesiology* 2021; **21**: 252. https://doi.org/10.1186/s12871-021-01473-9.
118. Bigler D, Adelhøj B, Petring OU, Pederson NO, Busch P, Kalhke P. Mental function and morbidity after acute hip surgery during spinal and general anaesthesia. *Anaesthesia* 1985; **40**: 672–6. https://doi.org/10.1111/j.1365-2044.1985.tb10949.x.
119. Bilgin TE, Bozlu M, Atici S, Cayan S, Tasdelen B. Wound Infiltration with Bupivacaine and Intramuscular Diclofenac Reduces Postoperative Tramadol Consumption in Patients Undergoing Radical Retropubic Prostatectomy: A Prospective, Double-blind, Placebo-controlled, Randomized Study. *Urology* 2011; **78**: 1281–5. https://doi.org/10.1016/j.urology.2011.07.1428.
120. Billa P, Deverakonda J, Krishna R. A Comparative Clinical Study of Anaesthetic Techniques for Lower Abdominal Laparoscopy: General versus Spinal Anaesthesia. *International Journal of Tropical Medicine,* 2023; **18**: 71-74. https://doi.org/10.36478/10.59218/makijtm.2023.4.71.74.
121. Bindra S, Oberoi D, Agarwal RK, Sarpal R. Ultrasound-guided Erector Spinae Plane Block for Postoperative Analgesia in Patients undergoing Open Nephrectomy: A Randomised Controlled Study. *Journal of Clinical and Diagnostic Research* 2023; **17**: 7–11.
122. Bindra TK, Chawla D, Kumar A, Kaur G, Kaur A. Comparison of 1% chloroprocaine in sub-arachnoid block with or without fentanyl for enhanced recovery after perianal surgeries. *Asian Journal of Pharmaceutical and Clinical Research* 2022: 131–4. https://doi.org/10.22159/ajpcr.2022.v15i7.44841.
123. Bjelland TW, Yates TGR, Fagerland MW, Frøyen JK, Lysebråten KR, Spreng UJ. Quadratus lumborum block for postoperative analgesia after full abdominoplasty: a randomized controlled trial. *Scandinavian Journal of Pain* 2019; **19**: 671–8. https://doi.org/10.1515/sjpain-2019-0013.
124. Bode RH, Lewis KP, Zarich SW et al. Cardiac Outcome after Peripheral Vascular Surgery. *Anesthesiology* 1996; **84**: 3–13. https://doi.org/10.1097/00000542-199601000-00002.
125. Bokesch PM, Huffnagle FT, Macauley C. Local Versus General Anesthesia for Lumbar Percutaneous Discectomy. *Journal of Neurosurgical Anesthesiology* 1993; **5**: 81–5. https://doi.org/10.1097/00008506-199304000-00003.
126. Borghi B, Casati A, Iuorio S et al. Frequency of hypotension and bradycardia during general anesthesia, epidural anesthesia, or integrated epidural-general anesthesia for total hip replacement. *Journal of Clinical Anesthesia* 2002; **14**: 102–6. https://doi.org/10.1016/S0952-8180(01)00362-2.
127. Borle AP, Chhabra A, Subramaniam R et al. Analgesic Efficacy of Paravertebral Bupivacaine During Percutaneous Nephrolithotomy: An Observer Blinded, Randomized Controlled Trial. *Journal of Endourology* 2014; **28**: 1085–90. https://doi.org/10.1089/end.2014.0179.
128. Borys M, Hanych A, Czuczwar M. Paravertebral Block Versus Preemptive Ketamine Effect on Pain Intensity after Posterolateral Thoracotomies: A Randomized Controlled Trial. *Journal of Clinical Medicine* 2020; **9**: 793. https://doi.org/10.3390/jcm9030793.
129. Borys M, Szajowska P, Jednakiewicz M et al. Quadratus Lumborum Block Reduces Postoperative Opioid Consumption and Decreases Persistent Postoperative Pain Severity in Patients Undergoing Both Open and Laparoscopic Nephrectomies—A Randomized Controlled Trial. *Journal of Clinical Medicine* 2021; **10**: 3590. https://doi.org/10.3390/jcm10163590.
130. Boselli E, Bouvet L, Augris-Mathieu C et al. Infraorbital and infratrochlear nerve blocks combined with general anaesthesia for outpatient rhinoseptoplasty: A prospective randomised, double-blind, placebo-controlled study. *Anaesthesia Critical Care & Pain Medicine* 2016; **35**: 31–6. https://doi.org/10.1016/j.accpm.2015.09.002.
131. Boughey JC, Goravanchi F, Parris RN et al. Prospective randomized trial of paravertebral block for patients undergoing breast cancer surgery. *The American Journal of Surgery* 2009; **198**: 720–5. https://doi.org/10.1016/j.amjsurg.2008.11.043.
132. Boulianne M, Paquet P, Veilleux R et al. Effects of quadratus lumborum block regional anesthesia on postoperative pain after colorectal resection: a randomized controlled trial. *Surgical Endoscopy* 2020; **34**: 4157–65. https://doi.org/10.1007/s00464-019-07184-0.
133. Bredtmann RD, Herden HN, Teichmann W et al. Epidural analgesia in colonic surgery: Results of a randomized prospective study. *Journal of British Surgery* 1990; **77**: 638–42. https://doi.org/10.1002/bjs.1800770615.
134. Breebaart MB, Aken DV, Fré OD et al. A prospective randomized double-blind trial of the efficacy of a bilateral lumbar erector spinae block on the 24h morphine consumption after posterior lumbar inter-body fusion surgery. *Trials* 2019; **20**: 441. https://doi.org/10.1186/s13063-019-3541-y.
135. Brix LD, Thillemann TM, Nikolajsen L. Local Anesthesia Combined With Sedation Compared With General Anesthesia for Ambulatory Operative Hysteroscopy: A Randomized Study. *Journal of PeriAnesthesia Nursing* 2016; **31**: 309–16. https://doi.org/10.1016/j.jopan.2015.09.009.
136. Brixel SM, Biboulet P, Swisser F et al. Posterior Quadratus Lumborum Block in Total Hip Arthroplasty: A Randomized Controlled Trial. *Anesthesiology* 2021; **134**: 722–33. https://doi.org/10.1097/ALN.0000000000003745.
137. Brodner G, Aken HV, Hertle L et al. Multimodal Perioperative Management—Combining Thoracic Epidural Analgesia, Forced Mobilization, and Oral Nutrition—Reduces Hormonal and Metabolic Stress and Improves Convalescence After Major Urologic Surgery. *Anesthesia & Analgesia* 2001; **92**: 1594–600. https://doi.org/10.1097/00000539-200106000-00049.
138. Broek RJC van den, Goeteyn J, Houterman S, Bouwman RA, Versyck BJB, Teijink JAW. Interpectoral-pectoserratus plane (PECS II) block in patients undergoing trans-axillary thoracic outlet decompression surgery; A prospective double-blind, randomized, placebo-controlled clinical trial. *Journal of clinical anesthesia* 2022; **82**: 110939. https://doi.org/10.1016/j.jclinane.2022.110939.
139. Brown DR, Hofer RE, Patterson DE et al. Intrathecal Anesthesia and Recovery from Radical Prostatectomy. *Anesthesiology* 2004; **100**: 926–34. https://doi.org/10.1097/00000542-200404000-00024.
140. Brozovich AA, Incavo SJ, Lambert BS et al. Intraosseous Morphine Decreases Postoperative Pain and Pain Medication Use in Total Knee Arthroplasty: A Double-Blind, Randomized Controlled Trial. *The Journal of Arthroplasty* 2022; **37**: S139–46. https://doi.org/10.1016/j.arth.2021.10.009.
141. Brunat G, Pouzeratte Y, Mann C, Didelot J-M, Rochon J-C, Eledjam J-J. Posterior perineal block with ropivacaine 0.75% for pain control during and after hemorrhoidectomy. *Regional anesthesia and pain medicine* 2003; **28**: 228–32. https://doi.org/10.1053/rapm.2003.50132.
142. Buggy DJ, Hall NA, Shah J, Brown J, Williams J. Motor block during patient-controlledepidural analgesia with ropivacaine or ropivacaine/fentanyl afterintrathecal bupivacaine for Caesareansection †. *British Journal of Anaesthesia* 2000; **85**: 468–70. https://doi.org/10.1093/bja/85.3.468.
143. Burden RJ, O’Kelly SW, Sutton D, Cumming J. Spermatic-cord block improves analgesia for day-case testicular surgery. *British journal of urology* 1997; **80**: 472–5. https://doi.org/10.1046/j.1464-410x.1997.00305.x.
144. Burlacu CL, Frizelle HP, Moriarty DC, Buggy DJ. Fentanyl and clonidine as adjunctive analgesics with levobupivacaine in paravertebral analgesia for breast surgery. *Anaesthesia* 2006; **61**: 932–7. https://doi.org/10.1111/j.1365-2044.2006.04793.x.
145. Butterfield NN, Schwarz SKW, Ries CR, Franciosi LG, Day B, MacLeod BA. Combined pre- and post-surgical bupivacaine would infiltrations decrease opioid requirements after knee ligament reconstruction. *Canadian Journal of Anesthesia/Journal canadien d’anesthésie* 2001; **48**: 245–50. https://doi.org/10.1007/BF03019753.
146. Büttner B, Mansur A, Hinz J, Erlenwein J, Bauer M, Bergmann I. Combination of general anesthesia and peripheral nerve block with low-dose ropivacaine reduces postoperative pain for several days after outpatient arthroscopy. *Medicine* 2017; **96**: e6046. https://doi.org/10.1097/MD.0000000000006046.
147. Cai H-D, Lin C-Z, Yu C-X, Lin X-Z. Bilateral Superficial Cervical Plexus Block Reduces Postoperative Nausea and Vomiting and Early Postoperative Pain after Thyroidectomy. *Journal of International Medical Research* 2012; **40**: 1390–8. https://doi.org/10.1177/147323001204000417.
148. Calenda E, Muraine M, Quintyn JC, Brasseur G. Sub‐Tenon infiltration or classical analgesic drugs to relieve postoperative pain. *Clinical & Experimental Ophthalmology* 2004; **32**: 154–8. https://doi.org/10.1111/j.1442-9071.2004.00793.x.
149. Caliskan B, Unlusoy EO, Karaca S. Effect of Preemptive Femoral Nerve Block on Pain Control and Opioid Consumption After Total Knee Arthroplasty: A Randomized Controlled Trial. *Medical Bulletin of Haseki* 2023; **61**: 312–8. https://doi.org/10.4274/haseki.galenos.2023.9549.
150. Campbell DNC, Lim M, Muir MK et al. A prospective randomised study of local versus general anaesthesia for cataract surgery. *Anaesthesia* 1993; **48**: 422–8. https://doi.org/10.1111/j.1365-2044.1993.tb07019.x.
151. Canıtez A, Kozanhan B, Aksoy N, Yildiz M, Tutar MS. Effect of erector spinae plane block on the postoperative quality of recovery after laparoscopic cholecystectomy: a prospective double-blind study. *British Journal of Anaesthesia* 2021; **127**: 629–35. https://doi.org/10.1016/j.bja.2021.06.030.
152. Cao X, Bao M, Ma Y et al. Hydromorphone combined with ropivacaine for erector spinae plane block in patients undergoing modified radical mastectomy: A prospective randomized controlled trial. *Medicine* 2024; **103**: e38758. https://doi.org/10.1097/MD.0000000000038758.
153. Çaparlar C, Altinsoy S, Akelma FK, Özhan M, Ergil J. Posterior Quadratus Lumborum Block Versus Posterior Transversus Abdominis Plane Block for Unilateral Inguinal Hernia Surgery. *Nigerian Journal of Clinical Practice* 2022; **25**: 1457–65. https://doi.org/10.4103/njcp.njcp_1876_21.
154. Caputo M, Alwair H, Rogers CA et al. Thoracic Epidural Anesthesia Improves Early Outcomes in Patients Undergoing Off-pump Coronary Artery Bypass Surgery. *Anesthesiology* 2011; **114**: 380–90. https://doi.org/10.1097/ALN.0b013e318201f571.
155. Carella M, Tran G, Bonhomme VL, Franssen C. Influence of Levobupivacaine Regional Scalp Block on Hemodynamic Stability, Intra- and Postoperative Opioid Consumption in Supratentorial Craniotomies: A Randomized Controlled Trial. *Anesthesia & Analgesia* 2021; **132**: 500–11. https://doi.org/10.1213/ANE.0000000000005230.
156. Carney J, McDonnell JG, Ochana A, Bhinder R, Laffey JG. The Transversus Abdominis Plane Block Provides Effective Postoperative Analgesia in Patients Undergoing Total Abdominal Hysterectomy. *Anesthesia & Analgesia* 2008; **107**: 2056–60. https://doi.org/10.1213/ane.0b013e3181871313.
157. Carron M, Freo U, Innocente F et al. Recovery Profiles of General Anesthesia and Spinal Anesthesia for Chemotherapeutic Perfusion with Circulatory Block (Stop-Flow Perfusion). *Anesthesia & Analgesia* 2007; **105**: 1500–3. https://doi.org/10.1213/01.ane.0000281051.24740.93.
158. Cartwright DP, Kvalsvik O, Cassuto J et al. A Randomized, Blind Comparison of Remifentanil and Alfentanil During Anesthesia for Outpatient Surgery. *Anesthesia & Analgesia* 1997; **85**: 1014–9. https://doi.org/10.1097/00000539-199711000-00011.
159. Casati A, Aldegheri G, Vinciguerra F, Marsan A, Fraschini G, Torri G. Randomized comparison between sevoflurane anaesthesia and unilateral spinal anaesthesia in elderly patients undergoing orthopaedic surgery. *European Journal of Anaesthesiology* 2005; **20**: 640–6. https://doi.org/10.1017/S0265021503001030.
160. Casati A, Cappelleri G, Aldegheri G, Marchetti C, Messina M, Ponti AD. Total intravenous anesthesia, spinal anesthesia or combined sciatic-femoral nerve block for outpatient knee arthroscopy. *Minerva anestesiologica* 2004; **70**: 493–502.
161. Casati A, Cappelleri G, Berti M, Fanelli G, Benedetto P, Torri G. Randomized comparison of remifentanil–propofol with a sciatic–femoral nerve block for out-patient knee arthroscopy. *European Journal of Anaesthesiology* 2002; **19**: 109. https://doi.org/10.1017/S0265021502000194.
162. Catro-Alves LJS, Azevedo VLFD, Braga TFDF, Goncalves AC, Oliveira GSD. The Effect of Neuraxial Versus General Anesthesia Techniques on Postoperative Quality of Recovery and Analgesia After Abdominal Hysterectomy. *Anesthesia & Analgesia* 2011; **113**: 1480–6. https://doi.org/10.1213/ANE.0b013e3182334d8b.
163. Cekic B, Geze S, Erturk E, Akdogan A, Eroglu A. A Comparison of Levobupivacaine and Levobupivacaine-Tramadol Combination in Bilateral Infraorbital Nerve Block for Postoperative Analgesia After Nasal Surgery. *Annals of Plastic Surgery* 2013; **70**: 131–4. https://doi.org/10.1097/SAP.0b013e318236baa8.
164. Çevikkalp E, Narmanlı M, Özgüç H, Ocakoğlu G. Bilateral 4-quadrant laparoscopic-assisted transversus abdominis plane block reduces early postoperative pain after laparoscopic cholecystectomy. *Saudi Medical Journal* 2023; **44**: 145–54. https://doi.org/10.15537/smj.2023.44.2.20220407.
165. Chai B, Yu H, Qian Y et al. Comparison of Postoperative Pain in 70 Women with Breast Cancer Following General Anesthesia for Mastectomy with and without Serratus Anterior Plane Nerve Block. *Medical Science Monitor* 2021; **28**. https://doi.org/10.12659/MSM.934064.
166. Chan A, Ng TKT, Tang BYH. Single-Shot Lumbar Erector Spinae Plane Block in Total Hip Replacement: A Randomized Clinical Trial. *Anesthesia & Analgesia* 2022; **135**: 829–36. https://doi.org/10.1213/ANE.0000000000006162.
167. Chang D-H, Lin Y-T, Chang C-W, Chen Y-S, Hsieh C-Y, Chang K-C. Use of a topical mix of lidocaine and prilocaine during split-thickness skin graft harvest improves postoperative recovery—A prospective randomized controlled trial. *Burns* 2022; **48**: 1396–404. https://doi.org/10.1016/j.burns.2021.08.023.
168. Charalambous CP, Tryfonidis M, Alvi F, Kumar R, Hirst P. Purely Intra-articular Versus General Anesthesia for Proposed Arthroscopic Partial Meniscectomy of the Knee: A Randomized Controlled Trial. *Arthroscopy: The Journal of Arthroscopic & Related Surgery* 2006; **22**: 972–7. https://doi.org/10.1016/j.arthro.2006.04.108.
169. Charier D, Court-Fortune I, Pereira B, Molliex S. Sleep disturbances and related disordered breathing after hip replacement surgery: A randomised controlled trial. *Anaesthesia Critical Care & Pain Medicine* 2021; **40**: 100927. https://doi.org/10.1016/j.accpm.2021.100927.
170. Charvin M, Longeras F, Jouve P et al. Effects of adding a combined femoral and sciatic nerve block with levobupivacaine and clonidine to general anaesthesia in femoropopliteal bypass surgery. *European Journal of Anaesthesiology* 2020; **37**: 787–95. https://doi.org/10.1097/EJA.0000000000001263.
171. Chassery C, Atthar V, Marty P et al. Opioid-free versus opioid-sparing anaesthesia in ambulatory total hip arthroplasty: a randomised controlled trial. *British Journal of Anaesthesia* 2024; **132**: 352–8. https://doi.org/10.1016/j.bja.2023.10.031.
172. Chatellier A, Dugué AE, Caufourier C, Maksud B, Compère JF, Bénateau H. Bloc du nerf alvéolaire inférieur par ropivacaïne : effets sur les nausées et vomissements en postopératoires (NVPO) des ostéotomies mandibulaires. *Revue de Stomatologie et de Chirurgie Maxillo-faciale* 2012; **113**: 417–22. https://doi.org/10.1016/j.stomax.2012.10.003.
173. Chen H, Song W, Wang W et al. Ultrasound-guided parasternal intercostal nerve block for postoperative analgesia in mediastinal mass resection by median sternotomy: a randomized, double-blind, placebo-controlled trial. *BMC Anesthesiology* 2021; **21**: 98. https://doi.org/10.1186/s12871-021-01291-z.
174. Chen L, He W, Liu X, Lv F, Li Y. Application of opioid-free general anesthesia for gynecological laparoscopic surgery under ERAS protocol: a non-inferiority randomized controlled trial. *BMC Anesthesiology* 2023; **23**: 34. https://doi.org/10.1186/s12871-023-01994-5.
175. Chen T-Y, Tseng C-C, Wang L-K, Tsai T-Y, Chen B-S, Chang C-L. The Clinical Use of Small-Dose Tetracaine Spinal Anesthesia for Transurethral Prostatectomy. *Anesthesia & Analgesia* 2001; **92**: 1020–3. https://doi.org/10.1097/00000539-200104000-00040.
176. Chen W-K, Ren L, Wei Y, Zhu D-X, Miao C-H, Xu J-M. General anesthesia combined with epidural anesthesia ameliorates the effect of fast-track surgery by mitigating immunosuppression and facilitating intestinal functional recovery in colon cancer patients. *International Journal of Colorectal Disease* 2015; **30**: 475–81. https://doi.org/10.1007/s00384-014-2098-1.
177. Chen W, Guo N, Wang S, Wang R, Huang F, Li S. General laryngeal mask airway anesthesia with lumbar plexus and sciatic block provides better outcomes than general anesthesia and endotracheal intubation in elderly patients undergoing hip surgery. *Archives of Gerontology and Geriatrics* 2018; **78**: 227–32. https://doi.org/10.1016/j.archger.2018.07.005.
178. Chen Y, Liu W, Gong X, Cheng Q. Comparison of Effects of General Anesthesia and Combined Spinal/Epidural Anesthesia for Cesarean Delivery on Umbilical Cord Blood Gas Values: A Double-Blind, Randomized, Controlled Study. *Medical Science Monitor* 2019; **25**: 5272–9. https://doi.org/10.12659/MSM.914160.
179. Chen Z, Jin Y, Lu G, Jin Y, Feng C, Zhao X. Preoperative Ultrasound-Guided Internal Branch Block of Superior Laryngeal Nerve Reduces Postoperative Sore Throat Caused by Double Lumen Endotracheal Intubation: A Randomized Trial. *Anesthesia & Analgesia* 2023; **137**: 1270–8. https://doi.org/10.1213/ANE.0000000000006534.
180. Cherprenet A-L, Rambourdin-Perraud M, Laforêt S et al. Local anaesthetic infiltration at the end of carotid endarterectomy improves post-operative analgesia. *Acta anaesthesiologica Scandinavica* 2015; **59**: 107–14. https://doi.org/10.1111/aas.12431.
181. Chiraya S, Singh J, Mitra S, Malhotra A, Srivastava MK, Fatima S. Erector spinae plane block for post-operative analgesia in thoracolumbar spine surgery: A randomised controlled trial. *Indian journal of anaesthesia* 2023; **67**: 985–90. https://doi.org/10.4103/ija.ija_357_23.
182. Cho D-Y, Drover DR, Nekhendzy V, Butwick AJ, Collins J, Hwang PH. The effectiveness of preemptive sphenopalatine ganglion block on postoperative pain and functional outcomes after functional endoscopic sinus surgery. *International Forum of Allergy & Rhinology* 2011; **1**: 212–8. https://doi.org/10.1002/alr.20040.
183. Cho H, Kwon H, Song S et al. Quality of postoperative recovery after upper-arm vascular surgery for hemodialysis in patients with end-stage renal disease: A prospective comparison of cervical epidural anesthesia vs general anesthesia. *Medicine* 2020; **99**: e18773. https://doi.org/10.1097/MD.0000000000018773.
184. Cho S, Kim YJ, Jeong K, Moon H-S. Ultrasound-guided bilateral rectus sheath block reduces early postoperative pain after laparoscopic gynecologic surgery: a randomized study. *Journal of Anesthesia* 2018; **32**: 189–97. https://doi.org/10.1007/s00540-018-2457-0.
185. Choi EK, Baek J, Kim DY. Effect of dexmedetomidine and remifentanil infusion on postoperative sore throat after lumbar spine surgery in the prone position. *Medicine* 2023; **102**: e33506. https://doi.org/10.1097/MD.0000000000033506.
186. Choi EK, Seo Y, Lim DG, Park S. Postoperative nausea and vomiting after thyroidectomy: a comparison between dexmedetomidine and remifentanil as part of balanced anesthesia. *Korean Journal of Anesthesiology* 2017; **70**: 299. https://doi.org/10.4097/kjae.2017.70.3.299.
187. Choi H, Huh J, Kim M, Moon SW, Kim KS, Hwang W. Opioid-Free Using Ketamine versus Opioid-Sparing Anesthesia during the Intraoperative Period in Video-Assisted Thoracoscopic Surgery: A Randomized Controlled Trial. *Journal of Personalized Medicine* 2024; **14**: 881. https://doi.org/10.3390/jpm14080881.
188. Choi H, Jung SH, Hong JM, Joo YH, Kim Y, Hong SH. Effects of Bilateral Infraorbital and Infratrochlear Nerve Block on Emergence Agitation after Septorhinoplasty: A Randomized Controlled Trial. *Journal of Clinical Medicine* 2019; **8**: 769. https://doi.org/10.3390/jcm8060769.
189. Choi H, Song JY, Oh EJ, Chae MS, Yu S, Moon YE. The Effect of Opioid-Free Anesthesia on the Quality of Recovery After Gynecological Laparoscopy: A Prospective Randomized Controlled Trial. *Journal of Pain Research* 2022; **15**: 2197–209. https://doi.org/10.2147/JPR.S373412.
190. Choi Y-M, Byeon G-J, Park S-J, Ok Y-M, Shin S-W, Yang K. Postoperative analgesic efficacy of single-shot and continuous transversus abdominis plane block after laparoscopic cholecystectomy: A randomized controlled clinical trial. *Journal of Clinical Anesthesia* 2017; **39**: 146–51. https://doi.org/10.1016/j.jclinane.2017.03.050.
191. Christopherson R, Glavan NJ, Norris EJ et al. Control of blood pressure and heart rate in patients randomized to epidural or general anesthesia for lower extremity vascular surgery. *Journal of Clinical Anesthesia* 1996; **8**: 578–84. https://doi.org/10.1016/S0952-8180(96)00139-0.
192. Chu H, Dong H, Wang Y, Niu Z. Effects of ultrasound-guided paravertebral block on MMP-9 and postoperative pain in patients undergoing VATS lobectomy: a randomized, controlled clinical trial. *BMC Anesthesiology* 2020; **20**: 59. https://doi.org/10.1186/s12871-020-00976-1.
193. Chung CJ, Yun SH, Hwang GB, Park JS, Chin YJ. Intrathecal fentanyl added to hyperbaric ropivacaine for cesarean delivery. *Regional anesthesia and pain medicine* 2002; **27**: 600–3. https://doi.org/10.1053/rapm.2002.36455.
194. Chung C, Choi J, Lee T, Park S. The Effects of Intraoperative Remifentanil Infusion on Postoperative Opioid Consumption in Patients Who Underwent Total Knee Arthroplasty with Femoral Nerve Block. *Journal of Clinical Medicine* 2023; **12**: 4975. https://doi.org/10.3390/jcm12154975.
195. Chung F, Westerling D, Chisholm LDJ, Squires GW. Postoperative recovery after general anaesthesia with and without retrobulbar block in retinal detachment surgery. *Anaesthesia* 1988; **43**: 943–6. https://doi.org/10.1111/j.1365-2044.1988.tb05657.x.
196. Cift T. Shoulder Tip Pain After Cesarean Section. *Journal of Clinical and Diagnostic Research* 2015; **9**: 4-6. https://doi.org/10.7860/JCDR/2015/13841.6314.
197. Ciftci B, Ekinci M, Celik EC, Tukac IC, Bayrak Y, Atalay YO. Efficacy of an Ultrasound-Guided Erector Spinae Plane Block for Postoperative Analgesia Management After Video-Assisted Thoracic Surgery: A Prospective Randomized Study. *Journal of cardiothoracic and vascular anesthesia* 2020; **34**: 444–9. https://doi.org/10.1053/j.jvca.2019.04.026.
198. Ciftci B, Ekinci M, Gölboyu BE et al. High Thoracic Erector Spinae Plane Block for Arthroscopic Shoulder Surgery: A Randomized Prospective Double-Blind Study. *Pain Medicine* 2021; **22**: 776–83. https://doi.org/10.1093/pm/pnaa359.
199. Clough TM, Sandher D, Bale RS, Laurence AS. The use of a local anesthetic foot block in patients undergoing outpatient bony forefoot surgery: A prospective randomized controlled trial. *The Journal of Foot [amp ] Ankle Surgery* 2003; **42**: ajfas0420024. https://doi.org/10.1053/jfas.2003.50001.
200. Cook PT, davies MJ, Cronin KD, Moran P. A Prospective Randomised Trial Comparing Spinal Anaesthesia using Hyperbaric Cinchocaine with General Anaesthesia for Lower Limb Vascular Surgery. *Anaesthesia and Intensive Care* 1986; **14**: 373–80. https://doi.org/10.1177/0310057X8601400409.
201. Copik M, Bialka S, Daszkiewicz A, Misiolek H. Thoracic paravertebral block for postoperative pain management after renal surgery. *European Journal of Anaesthesiology* 2017; **34**: 596–601. https://doi.org/10.1097/EJA.0000000000000673.
202. Copik MM, Sadowska D, Smereka J, Czyzewski D, Misiołek HD, Białka S. Assessment of feasibility of opioid-free anesthesia combined with preoperative thoracic paravertebral block and postoperative intravenous patient-controlled analgesia oxycodone with non-opioid analgesics in the perioperative anesthetic management for video-assisted thoracic surgery. *Anaesthesiology Intensive Therapy* 2024; **56**: 98–107. https://doi.org/10.5114/ait.2024.141279.
203. Coşarcan SK, Gürkan Y, Manici M et al. The effect of ultrasound-guided rectus sheath block on postoperative analgesia in robot assisted prostatectomy: A randomized controlled trial. *Medicine* 2024; **103**: e37975. https://doi.org/10.1097/MD.0000000000037975.
204. Covotta M, Claroni C, Costantini M et al. The Effects of Ultrasound-Guided Transversus Abdominis Plane Block on Acute and Chronic Postsurgical Pain After Robotic Partial Nephrectomy: A Prospective Randomized Clinical Trial. *Pain Medicine* 2020; **21**: 378–86. https://doi.org/10.1093/pm/pnz214.
205. Cros J, Sengès P, Kaprelian S et al. Pectoral I Block Does Not Improve Postoperative Analgesia After Breast Cancer Surgery. *Regional Anesthesia and Pain Medicine* 2018; **43**: 596–604. https://doi.org/10.1097/AAP.0000000000000779.
206. Cui Y, Liu K, He L et al. Comparison of two different anesthesia methods on the quality of surgical condition in transvaginal natural-orifice transluminal endoscopic surgery (vNOTES): A prospective single-blind randomized clinical trial. *Perioperative Care and Operating Room Management* 2022; **27**: 100253. https://doi.org/10.1016/j.pcorm.2022.100253.
207. Čustovic S, Pandža H, Delibegovic S. Effect of Local Anesthesia on the Postoperative Pain After Laparoscopic Appendectomy. *Journal of Laparoendoscopic & Advanced Surgical Techniques* 2019; **29**: 65–71. https://doi.org/10.1089/lap.2018.0452.
208. Dabbagh A, Elyasi H. The role of paravertebral block in decreasing postoperative pain in elective breast surgeries. *Medical science monitor : international medical journal of experimental and clinical research* 2007; **13**: CR464-7.
209. Dabhi N, Dalwadi B, Chandnani A, Bumiya C. Efficacy of Ultrasound‐Guided Erector Spinae Plane Block for Postoperative Analgesia in Modified Radical Mastectomy: a Prospective, Randomized, Clinical Study. *Research Journal of Medical Sciences* 2023; **17**: 35‐41. https://doi.org/10.59218/makrjms.2023.8.35.41.
210. Dahl JB, Schultz P, Anker-Møller E, Christensen EF, Staunstrup HG, Carlsson P. Spinal anaesthesia in young patients using a 29-gauge needle: technical considerations and an evaluation of postoperative complaints compared with general anaesthesia. *British journal of anaesthesia* 1990; **64**: 178–82. https://doi.org/10.1093/bja/64.2.178.
211. Dai J, Li S, Weng Q, Long J, Wu D. Opioid-free anesthesia with ultrasound-guided quadratus lumborum block in the supine position for lower abdominal or pelvic surgery: a randomized controlled trial. *Scientific Reports* 2024; **14**: 4652. https://doi.org/10.1038/s41598-024-55370-5.
212. Dai X, Xing D, Luo J et al. Fascia iliaca compartment block mitigates the fluctuations in heart rate variability and reduces pain with opioid consumption in elderly individuals with hip fractures: A randomized controlled trial. *Heliyon* 2024; **10**: e27375. https://doi.org/10.1016/j.heliyon.2024.e27375.
213. Dalai H, Panigrahi C, Soren DK, Sahu PC, Panda J, Jena SK. DEXMEDETOMIDINE AND ROPIVACAINE IN THORACIC EPIDURAL ANAESTHESIA FOR BREAST SURGERY- A CLINICAL COMPARISON WITH GENERAL ANAESTHESIA. *Journal of Evolution of Medical and Dental Sciences* 2017; **6**: 2149–53. https://doi.org/10.14260/Jemds/2017/466.
214. Dam M, Hansen C, Poulsen TD et al. Transmuscular quadratus lumborum block reduces opioid consumption and prolongs time to first opioid demand after laparoscopic nephrectomy. *Regional Anesthesia & Pain Medicine* 2021; **46**: 18–24. https://doi.org/10.1136/rapm-2020-101745.
215. Danelli G, Berti M, Casati A et al. Spinal block or total intravenous anaesthesia with propofol and remifentanil for gynaecological outpatient procedures. *European Journal of Anaesthesiology* 2002; **19**: 594. https://doi.org/10.1017/S0265021502000960.
216. Dar M, Malik S, Dar Y et al. Comparison of percutaneous nephrolithotomy under epidural anesthesia versus general anesthesia: A randomized prospective study. *Urology Annals* 2021; **13**: 210. https://doi.org/10.4103/UA.UA_82_20.
217. Das B, Kumar D, Sinha PK. A Randomized Comparative Clinical Assessment of Postoperative Analgesic Efficacy of Epidural Ropivacaine and Ropivacaine with Tramadol in Patients Undergoing Abdominal Surgeries: a Prospective Clinical Study. *International Journl of Current Pharmaceutical* 2023; **15**: 246–53.
218. Das S, Bhattacharya P, Mandal M, Mukhopadhyay S, Basu S, Mandol B. Multiple-injection thoracic paravertebral block as an alternative to general anaesthesia for elective breast surgeries: A randomised controlled trial. *Indian Journal of Anaesthesia* 2012; **56**: 27. https://doi.org/10.4103/0019-5049.93340.
219. Dauphin A, Raymer KE, Stanton EB, Fuller HD. Comparison of general anesthesia with and without lumbar epidural for total hip arthroplasty: Effects of epidural block on hip arthroplasty. *Journal of Clinical Anesthesia* 1997; **9**: 200–3. https://doi.org/10.1016/S0952-8180(97)00035-4.
220. Deepika V, Ahuja V, Thapa D, Gombar S, Gupta N. Evaluation of analgesic efficacy of superficial cervical plexus block in patients undergoing modified radical mastoidectomy: A randomised controlled trial. *Indian Journal of Anaesthesia* 2021; **65**: S115–20. https://doi.org/10.4103/ija.ija_339_21.
221. DeMaria S, Govindaraj S, Chinosorvatana N, Kang S, Levine AI. Bilateral Sphenopalatine Ganglion Blockade Improves Postoperative Analgesia after Endoscopic Sinus Surgery. *American Journal of Rhinology & Allergy* 2012; **26**: e23–7. https://doi.org/10.2500/ajra.2012.26.3709.
222. Demiraran Y, Albayrak M, Yorulmaz IS, Ozdemir I. Tramadol and levobupivacaine wound infiltration at Cesarean delivery for postoperative analgesia. *Journal of Anesthesia* 2013; **27**: 175–9. https://doi.org/10.1007/s00540-012-1510-7.
223. Demirel CB, Kalayci M, Ozkocak I, Altunkaya H, Ozer Y, Acikgoz B. A Prospective Randomized Study Comparing Perioperative Outcome Variables After Epidural or General Anesthesia for Lumbar Disc Surgery. *Journal of Neurosurgical Anesthesiology* 2003; **15**: 185–92. https://doi.org/10.1097/00008506-200307000-00005.
224. Dershwitz M, Michałowski P, Chang Y, Rosow CE, Conlay LA. Postoperative nausea and vomiting after total intravenous anesthesia with propofol and remifentanil or alfentanil: how important is the opioid? *Journal of Clinical Anesthesia* 2002; **14**: 275–8. https://doi.org/10.1016/S0952-8180(02)00353-7.
225. Dhawan S, Guri M, Bhati K, Aeron N. Comparison of airway blocks versus general anaesthesia for diagnostic direct laryngoscopy: A randomised comparative trial. *Indian Journal of Anaesthesia* 2020; **64**: 1054. https://doi.org/10.4103/ija.IJA_680_20.
226. Dieudonne N, Gomola A, Bonnichon P, Ozier YM. Prevention of Postoperative Pain After Thyroid Surgery: A Double-Blind Randomized Study of Bilateral Superficial Cervical Plexus Blocks. *Anesthesia & Analgesia* 2001; **92**: 1538–42. https://doi.org/10.1097/00000539-200106000-00038.
227. Dolatabadi M, Motlagh djalali, Maleki A, Norouzi Z. Comparison of Sedation Efficacy Between Remifentanil and Dexmedetomidine in Arteriovenous Fistula Placement Surgery. *Iranian Heart Journal* 2022: 84–90.
228. Donmez T, Erdem VM, Sunamak O, Erdem DA, Avaroglu HI. Laparoscopic total extraperitoneal repair under spinal anesthesia versus general anesthesia: a randomized prospective study. *Therapeutics and Clinical Risk Management* 2016; **Volume 12**: 1599–608. https://doi.org/10.2147/TCRM.S117891.
229. Doo AR, Zhang X, Wang J et al. A COMPARISON OF THE EFFECTS OF GENERAL ANESTHESIA VERSUS SPINAL ANESTHESIA ON CATHETER-RELATED BLADDER DISCOMFORT AFTER HoLEP: A PROSPECTIVE TRIAL. *Journal of Men’s Health* 2020; **16**: 25-34. https://doi.org/10.15586/jomh.v16iSP1.227.
230. Dost B, Kaya C, Ozdemir E et al. Ultrasound-guided erector spinae plane block for postoperative analgesia in patients undergoing open radical prostatectomy: A randomized, placebo-controlled trial. *Journal of Clinical Anesthesia* 2021; **72**: 110277. https://doi.org/10.1016/j.jclinane.2021.110277.
231. Du H, Liu X, Li F, Xue Z, Li Y, Qian B. Anesthetic effect of ultrasound-guided multiple-nerve blockade in modified radical mastectomy in patients with breast cancer. *Medicine* 2021; **100**: e24786. https://doi.org/10.1097/MD.0000000000024786.
232. Duan L, Wang Z, Sun M, Huang L, Ye Q, Wang H. Effect of Ultrasound-Guided Erector Spinae Plane Block on Pain After Laparoscopic Transabdominal Preperitoneal Repair: A Prospective, Double-Blind, Randomized Controlled Study. *Altern Ther Health Med* 2024; **30**: 366-74.
233. Dubilet M, Gruenbaum BF, Semyonov M et al. Erector Spinae Plane (ESP) Block for Postoperative Pain Management after Open Oncologic Abdominal Surgery. *Pain Research and Management* 2023; **2023**: 1–9. https://doi.org/10.1155/2023/9010753.
234. Durán-Vega HC, Ramírez-Montañana A, Galindo OG et al. Ropivacaine in Breast Augmentation Surgery. *Plastic and Reconstructive Surgery - Global Open* 2018; **6**: e1745. https://doi.org/10.1097/GOX.0000000000001745.
235. Ege B, Ege M, Koparal M, Alan H. Comparison of the Anesthetic Efficiency of Lidocaine and Tramadol Hydrochloride in Orthodontic Extractions: A Split-Mouth, Prospective, Randomized, Double-Blind Study. *Journal of Oral and Maxillofacial Surgery* 2020; **78**: 52–62. https://doi.org/10.1016/j.joms.2019.07.010.
236. Eid GM, Elyazed MMA, Mostafa SF, Elghafar MSA. Ultrasound-Guided Erector Rhomboid Intercostal and Subserratus Plane Block (RISS) for perioperative analgesia in patients undergoing thoracotomy: a prospective randomized controlled study. *Acta Anaesthesiologica Belgica* 2024; **75**: 287–95. https://doi.org/10.56126/75.4.58.
237. Ekici NY, Alagöz S. The effectiveness of endoscopic sphenopalatine ganglion block in management of postoperative pain after septal surgery. *International Forum of Allergy & Rhinology* 2019; **9**: 1521–5. https://doi.org/10.1002/alr.22411.
238. Ekinci M, Ciftci B, Celik EC, Karakaya MA, Demiraran Y. The Efficacy of Different Volumes on Ultrasound-Guided Type-I Pectoral Nerve Block for Postoperative Analgesia After Subpectoral Breast Augmentation: A Prospective, Randomized, Controlled Study. *Aesthetic Plastic Surgery* 2019; **43**: 297–304. https://doi.org/10.1007/s00266-019-01322-8.
239. El-Dawlatly AA, Turkistani A, Kettner SC et al. Ultrasound-guided transversus abdominis plane block: description of a new technique and comparison with conventional systemic analgesia during laparoscopic cholecystectomy. *British Journal of Anaesthesia* 2009; **102**: 763–7. https://doi.org/10.1093/bja/aep067.
240. El-Hakim H, Nunez DA, Saleh HA, MacLeod DM, Gardiner Q. A randomised controlled trial of the effect of regional nerve blocks on immediate post-tonsillectomy pain in adult patients. *Clinical Otolaryngology and Allied Sciences* 2000; **25**: 413–7. https://doi.org/10.1046/j.1365-2273.2000.00397.x.
241. Elfadel IA, Fouad G, Hazem A, Elrahman AA, Saleh M. A Comparative Study between Erector Spinae Plane Block with General Anesthesia versus Conventional Intravenous Analgesics with General Anesthesia in Patients undergoing Conserving Surgery for Breast Cancer. *Egyptian Journal of Anaesthesia* 2022; **38**: 383–9. https://doi.org/10.1080/11101849.2022.2092302.
242. Elghamry MR, Messbah WE, Abduallah MA, Elrahwan SM. Role of ultrasound-guided sacral erector spinae plane block for post-operative analgesia in pilonidal sinus surgery: A randomised trial. *Journal of Anaesthesiology Clinical Pharmacology* 2024; **40**: 653–8. https://doi.org/10.4103/joacp.joacp_226_23.
243. Elkassabany N, Ahmed M, Malkowicz SB, Heitjan DF, Isserman JA, Ochroch EA. Comparison between the analgesic efficacy of transversus abdominis plane (TAP) block and placebo in open retropubic radical prostatectomy: a prospective, randomized, double-blinded study. *Journal of Clinical Anesthesia* 2013; **25**: 459–65. https://doi.org/10.1016/j.jclinane.2013.04.009.
244. Ellakany M. Comparative study between general and thoracic spinal anesthesia for laparoscopic cholecystectomy. *Egyptian Journal of Anaesthesia* 2013; **29**: 375–81. https://doi.org/10.1016/j.egja.2013.05.004.
245. Ellatif SEA, Abdelnaby SM. Ultrasound guided erector spinae plane block versus quadratus lumborum block for postoperative analgesia in patient undergoing open nephrectomy: A randomized controlled study. *Egyptian Journal of Anaesthesia* 2021; **37**: 123–34. https://doi.org/10.1080/11101849.2021.1894661.
246. Elliott BM, Collins GJ, Youkey JR, Donohue HJ, Salander JM, Rich NM. Intraoperative local anesthetic injection of the carotid sinus nerve. *The American Journal of Surgery* 1986; **152**: 695–9. https://doi.org/10.1016/0002-9610(86)90451-4.
247. Elnakera A, Elsayed A, Gaafar TY, Elnakera AM. Enhanced Recovery after lumbar laminectomy using combined epidural and general anesthesia with tolerable endotracheal tube: Randomized controlled study. *Ain-Shams Journal of Anesthesiology* 2024; **16**: 1–9. https://doi.org/10.21608/asja.2024.250869.1018.
248. Elsabeeny WY, Elsamahy K, Elazab AM, Ibrahim MA. Feasibility of segmental thoracic epidural anesthesia in cancer patients undergoing feeding jejunostomy: A randomized controlled trial. *Anaesthesia, Pain & Intensive Care* 2021; **25**. https://doi.org/10.35975/apic.v25i5.1623.
249. Eltaher E, Nasr N, Abuelnaga ME, Elgawish Y. Effect of Ultrasound-Guided Thoracolumbar Interfascial Plane Block on the Analgesic Requirements in Patients Undergoing Lumbar Spine Surgery Under General Anesthesia: A Randomized Controlled Trial. *Journal of Pain Research* 2021; **14**: 3465–74. https://doi.org/10.2147/JPR.S329158.
250. Elyazed MMA, Mostafa SF, Abdelghany MS, Eid GM. Ultrasound-Guided Erector Spinae Plane Block in Patients Undergoing Open Epigastric Hernia Repair: A Prospective Randomized Controlled Study. *Anesthesia & Analgesia* 2019; **129**: 235–40. https://doi.org/10.1213/ANE.0000000000004071.
251. Elzohry AAM, Hegab AS, Khalifa OYA, Elhossieny KM, Hameed FAZHA. Safety and Efficacy of Ultrasound-Guided Combined Segmental Thoracic Spinal Epidural Anesthesia in Abdominal Surgeries and Laparoscopic Procedures: A Prospective Randomized Clinical Study. *Anesthesiology and Pain Medicine* 2024; **13**. https://doi.org/10.5812/aapm-138825.
252. Emile SH, Abdel-Razik MA, Elbahrawy K et al. Impact of Ultrasound-Guided Transversus Abdominis Plane Block on Postoperative Pain and Early Outcome After Laparoscopic Bariatric Surgery: a Randomized Double-Blinded Controlled Trial. *Obesity Surgery* 2019; **29**: 1534–41. https://doi.org/10.1007/s11695-019-03720-y.
253. Emile SH, Elfeki H, Elbahrawy K, Sakr A, Shalaby M. Ultrasound-guided versus laparoscopic-guided subcostal transversus abdominis plane (TAP) block versus No TAP block in laparoscopic cholecystectomy; a randomized double-blind controlled trial. *International Journal of Surgery* 2022; **101**: 106639. https://doi.org/10.1016/j.ijsu.2022.106639.
254. Erdem VM, Donmez T, Uzman S, Ferahman S, Hatipoglu E, Sunamak O. Spinal/epidural block as an alternative to general anesthesia for laparoscopic appendectomy: a prospective randomized clinical study. *Videosurgery and Other Miniinvasive Techniques* 2018; **13**: 148–56. https://doi.org/10.5114/wiitm.2018.72684.
255. Erdogan E, Ozenc E. Factors associated with acute and chronic pain after inguinal herniorraphy. *Romanian journal of anaesthesia and intensive care* 2018; **25**: 31–5. https://doi.org/10.21454/rjaic.7518.251.oze.
256. Erdogan MA, Ozgul U, Uçar M et al. Effect of transversus abdominis plane block in combination with general anesthesia on perioperative opioid consumption, hemodynamics, and recovery in living liver donors: The prospective, double‐blinded, randomized study. *Clinical Transplantation* 2017; **31**. https://doi.org/10.1111/ctr.12931.
257. Erhan E, Ugur G, Anadolu O, Saklayan M, Ozyar B. General anaesthesia or spinal anaesthesia for outpatient urological surgery. *European Journal of Anaesthesiology* 2005; **20**: 647–52. https://doi.org/10.1017/S0265021503001042.
258. Eskin MB, Ceylan A, Özhan MÖ, Atik B. Ultrasound-guided erector spinae block versus mid-transverse process to pleura block for postoperative analgesia in lumbar spinal surgery. *Der Anaesthesist* 2020; **69**: 742–50. https://doi.org/10.1007/s00101-020-00848-w.
259. Espelund M, Fomsgaard JS, Haraszuk J, Mathiesen O, Dahl JB. Analgesic efficacy of ultrasound-guided adductor canal blockade after arthroscopic anterior cruciate ligament reconstruction. *European Journal of Anaesthesiology* 2013; **30**: 422–8. https://doi.org/10.1097/EJA.0b013e328360bdb9.
260. Eti Z, Irmak P, Gulluoglu BM, Manukyan MN, Gogus FY. Does Bilateral Superficial Cervical Plexus Block Decrease Analgesic Requirement After Thyroid Surgery? *Anesthesia & Analgesia* 2006; **102**: 1174–6. https://doi.org/10.1213/01.ane.0000202383.51830.c4.
261. Ezeh UO, Shoulder VS, Breeson AD, Lamb MD, Vellacott ID, Martin JL. Local anaesthetic on Filshie clips for pain relief after tubal sterilisation: a randomised double-blind controlled trial. *The Lancet* 1995; **346**: 82–5. https://doi.org/10.1016/S0140-6736(95)92112-5.
262. Fabris L, Maretic A. Effects of general anaesthesia versus spinal anaesthesia for caesarean section on postoperative analgesic consumption and postoperative pain. *Periodicum biologorum* 2009; **111**.
263. Fadhlurrahman AF, Setiawan P, Sumartono C, Perdhana F, Husain TA. The effect of pectointercostal fascial block on stress response in open heart surgery. *Saudi Journal of Anaesthesia* 2024; **18**: 70–6. https://doi.org/10.4103/sja.sja_349_23.
264. Fan Q, Luo J, Zhou Q et al. Esketamine opioid-free intravenous anesthesia versus opioid intravenous anesthesia in spontaneous ventilation video-assisted thoracic surgery: a randomized controlled trial. *Frontiers in Oncology* 2023; **13**. https://doi.org/10.3389/fonc.2023.1145953.
265. Fan R, Zhao L, Hong H. Effect of inhalation anesthesia combined with nerve block on improving postoperative cognitive function in elderly orthopedic patients. *Biomedical Research* 2017; **28**: 4485–9.
266. Farbood A, Asadi S, Amini A et al. The effect of posterior tibial and sural nerve blocks on postoperative pain of patients following open reduction and internal fixation of calcaneal fractures. *Foot and Ankle Surgery* 2022; **28**: 858–62. https://doi.org/10.1016/j.fas.2021.11.009.
267. Farmery AD, Shlugman D, Rahman R, Rosen P. Sub-Tenon’s block reduces both intraoperative and postoperative analgesia requirement in vitreo-retinal surgery under general anaesthesia. *European Journal of Anaesthesiology* 2005; **20**: 973–8. https://doi.org/10.1017/S0265021503001571.
268. Faunø P, Lund B, Christiansen SE, Gjøderum O, Lind M. Analgesic Effect of Hamstring Block After Anterior Cruciate Ligament Reconstruction Compared With Placebo: A Prospective Randomized Trial. *Arthroscopy: The Journal of Arthroscopic & Related Surgery* 2015; **31**: 63–8. https://doi.org/10.1016/j.arthro.2014.07.024.
269. Favaro M de L, Gabor S, Souza DBF, Araújo AA, Milani ALC, Junior MAFR. Quadratus Lumborum Block As A Single Anesthetic Method For Laparoscopic Totally Extraperitoneal (Tep) Inguinal Hernia Repair: A Randomized Clinical Trial. *Scientific Reports* 2020; **10**: 8526. https://doi.org/10.1038/s41598-020-65604-x.
270. Feng C, Xu Y, Chen S et al. Opioid-free anaesthesia reduces postoperative nausea and vomiting after thoracoscopic lung resection: a randomised controlled trial. *British Journal of Anaesthesia* 2024; **132**: 267–76. https://doi.org/10.1016/j.bja.2023.11.008.
271. Fields AC, Gonzalez DO, Chin EH, Nguyen SQ, Zhang LP, Divino CM. Laparoscopic-Assisted Transversus Abdominis Plane Block for Postoperative Pain Control in Laparoscopic Ventral Hernia Repair: A Randomized Controlled Trial. *Journal of the American College of Surgeons* 2015; **221**: 462–9. https://doi.org/10.1016/j.jamcollsurg.2015.04.007.
272. Fong S. Assessment of wound infiltration with bupivacaine in women undergoing day-case gynecological laparoscopy. *Regional Anesthesia and Pain Medicine* 2001; **26**: 131–6. https://doi.org/10.1053/rapm.2001.21836.
273. Fredman B, Zohar E, Ganim T, Shalev M, Jedeikin R. BUPIVACAINE INFILTRATION INTO THE NEUROVASCULAR BUNDLE OF THE PROSTATIC NERVE DOES NOT IMPROVE POSTOPERATIVE PAIN OR RECOVERY FOLLOWING TRANSVESICAL PROSTATECTOMY. *Journal of Urology* 1998; **159**: 154–7. https://doi.org/10.1016/S0022-5347(01)64040-0.
274. Freir NM, Murphy C, Mugawar M, Linnane A, Cunningham AJ. Transversus Abdominis Plane Block for Analgesia in Renal Transplantation. *Anesthesia & Analgesia* 2012; **115**: 953–7. https://doi.org/10.1213/ANE.0b013e3182642117.
275. Fu Y, Fu H, Wei W et al. Effect of bilateral low serratus anterior plane block on quality of recovery after trans-subxiphoid robotic thymectomy: Results of a randomized placebo-controlled trial. *International Journal of Medical Sciences* 2024; **21**: 1241–9. https://doi.org/10.7150/ijms.91797.
276. Fujimoto H, Irie T, Mihara T, Mizuno Y, Nomura T, Goto T. Effect of posterior quadratus lumborum blockade on the quality of recovery after major gynaecological laparoscopic surgery: A randomized controlled trial. *Anaesthesia and Intensive Care* 2019; **47**: 146–51. https://doi.org/10.1177/0310057X19838765.
277. Fyneface-Ogan S, Uzoigwe SA. Caesarean section outcome in eclamptic patients: a comparison of infiltration and general anaesthesia. *West African journal of medicine* 2008; **27**: 250–4.
278. Galetin T, Eckermann C, Defosse J, Kraja O, Lopez-Pastorini A. PATIENTS’ SATISFACTION WITH LOCAL OR GENERAL ANAESTHESIA IN VIDEO-ASSISTED THORACOSCOPIC SURGERY: RESULTS OF THE RANDOMIZED CONTROLLED PASSAT TRIAL. *Cheste Journal* 2022; **161**.
279. Galetin T, Eckermann C, Defosse JM et al. Patients’ satisfaction with local and general anaesthesia for video-assisted thoracoscopic surgery—results of the first randomized controlled trial PASSAT. *European Journal of Cardio-Thoracic Surgery* 2023; **63**. https://doi.org/10.1093/ejcts/ezad046.
280. Galos DK, Taormina DP, Crespo A et al. Does Brachial Plexus Blockade Result in Improved Pain Scores After Distal Radius Fracture Fixation? A Randomized Trial. *Clinical Orthopaedics & Related Research* 2016; **474**: 1247–54. https://doi.org/10.1007/s11999-016-4735-1.
281. Gao X, Wang S, Li Y, Zhou D, Peng X. Clinical Analysis of Different Anesthesia and Analgesia Methods for Patients Undergoing Uniportal Video-assisted Lung Surgery. *Clinical Therapeutics* 2024; **46**: 570–5. https://doi.org/10.1016/j.clinthera.2024.06.009.
282. Gasteiger L, Fiala A, Naegele F et al. The Impact of Preoperative Combined Pectoserratus and/or Interpectoral Plane (Pectoralis Type II) Blocks on Opioid Consumption, Pain, and Overall Benefit of Analgesia in Patients Undergoing Minimally Invasive Cardiac Surgery: A Prospective, Randomized, Controlled, and Triple-blinded Trial. *Journal of Cardiothoracic and Vascular Anesthesia* 2024; **38**: 2973–81. https://doi.org/10.1053/j.jvca.2024.06.036.
283. Gaszynski TM, Strzelczyk JM, Gaszynski WP. Post-anesthesia Recovery after Infusion of Propofol with Remifentanil or Alfentanil or Fentanyl in Morbidly Obese Patients. *Obesity Surgery* 2004; **14**: 498–503. https://doi.org/10.1381/096089204323013488.
284. Gautam B, Tabdar S, Shrestha U. Comparison of Fentanyl and Dexmedetomidine as Intrathecal Adjuvants to Spinal Anaesthesia for Abdominal Hysterectomy. *Journal of Nepal Medical Association* 2018; **56**: 848–55. https://doi.org/10.31729/jnma.3739.
285. Gebhardt V, Monnard M, Weiss C, Schmittner M. Discharge times for knee arthroscopy in spinal vs. general anesthesia. *Open Medicine* 2014; **9**: 446–54. https://doi.org/10.2478/s11536-013-0303-1.
286. Gebhardt V, Zawierucha V, Schöffski O, Schwarz A, Weiss C, Schmittner MD. Spinal anaesthesia with chloroprocaine 1% versus total intravenous anaesthesia for outpatient knee arthroscopy. *European Journal of Anaesthesiology* 2018; **35**: 774–81. https://doi.org/10.1097/EJA.0000000000000794.
287. Ghali AM, Btarny AME. The effect on outcome of peribulbar anaesthesia in conjunction with general anesthesia for vitreoretinal surgery. *Anaesthesia* 2010; **65**: 249–53. https://doi.org/10.1111/j.1365-2044.2009.06191.x.
288. Ghamry ME, Elgebaly AS, Anwar AG, Shaddad MN. Ultrasound-guided erector spinae plane block for acute pain management in patients undergoing posterior lumbar interbody fusion under general anaesthesia. *Southern African Journal of Anaesthesia and Analgesia* 2019; **25**: 26–31. https://doi.org/10.36303/SAJAA.2019.25.6.A4.
289. Ghasemi A, Chamanara M, Paknejad B, Yousefizoshk M, Hazrati E. Dexmedetomidine versus sufentanil as adjuvants to bupivacaine for brachial plexus block during upper extremity surgery: a randomized clinical trial. *Brazilian Journal of Anesthesiology (English Edition)* 2023; **73**: 736–43. https://doi.org/10.1016/j.bjane.2021.03.026.
290. Ghezal H, Bouvet S, Kabani S, Ripart J, Cuvillon P. Ropivacaine versus placebo on postoperative analgesia and chronic pain following third molar extraction: A Prospective Randomized Controlled Study. *Journal of Stomatology, Oral and Maxillofacial Surgery* 2020; **121**: 113–7. https://doi.org/10.1016/j.jormas.2019.07.005.
291. Ghimire A, Bhattarai B, Prasad JN, Shah SP. The Analgesic Effectiveness of Ipsilateral Transversus Abdominis Plane Block in Adult Patients Undergoing Appendectomy: A Prospective Randomized Controlled Trial. *Kathmandu University Medical Journal* 2017; **13**: 361–5. https://doi.org/10.3126/kumj.v13i4.16838.
292. Gökçek E, Kozan G. Postoperative effects of bilateral sphenopalatine ganglion blockade in septorhinoplasty operations; double-blind randomized clinical trial. *Brazilian Journal of Otorhinolaryngology* 2024; **90**: 101373. https://doi.org/10.1016/j.bjorl.2023.101373.
293. Goldstein RY, Montero N, Jain SK, Egol KA, Tejwani NC. Efficacy of Popliteal Block in Postoperative Pain Control After Ankle Fracture Fixation. *Journal of Orthopaedic Trauma* 2012; **26**: 557–61. https://doi.org/10.1097/BOT.0b013e3182638b25.
294. Gonano C, Kettner SC, Ernstbrunner M, Schebesta K, Chiari A, Marhofer P. Comparison of economical aspects of interscalene brachial plexus blockade and general anaesthesia for arthroscopic shoulder surgery. *British Journal of Anaesthesia* 2009; **103**: 428–33. https://doi.org/10.1093/bja/aep173.
295. Gonano C, Leitgeb U, Sitzwohl C, Ihra G, Weinstabl C, Kettner SC. Spinal Versus General Anesthesia for Orthopedic Surgery: Anesthesia Drug and Supply Costs. *Anesthesia & Analgesia* 2006; **102**: 524–9. https://doi.org/10.1213/01.ane.0000194292.81614.c6.
296. Gönüllü NN, Cubukçu A, Alponat A. Comparison of local and general anesthesia in tension-free (Lichtenstein) hernioplasty: a prospective randomized trial. *Hernia : the journal of hernias and abdominal wall surgery* 2002; **6**: 29–32. https://doi.org/10.1007/s10029-002-0040-x.
297. Good RP, Snedden MH, Schieber FC, Polachek A. Effects of a preoperative femoral nerve block on pain management and rehabilitation after total knee arthroplasty. *American journal of orthopedics (Belle Mead, N.J.)* 2007; **36**: 554–7.
298. Gordon SM, Dionne RA, Brahim J, Jabir F, Dubner R. Blockade of peripheral neuronal barrage reduces postoperative pain. *Pain* 1997; **70**: 209–15. <https://doi.org/10.1016/S0304-3959(96)03315-5>.
299. Gorur S, Inanoglu K, Akkurt BCO, Candan Y, Kiper AN. Periprostatic Nerve Blockage Reduces Postoperative Analgesic Consumption and Pain Scores of Patients Undergoing Transurethral Prostate Resection. *Urologia Internationalis* 2007; **79**: 297–301. https://doi.org/10.1159/000109712.
300. Gottfredsdóttir MS, Gislason I, Stefánsson E, Sigurjónsdóttir S, Nielsen NC. Effects of retrobulbar bupivacaine on post‐operative pain and nausea in retinal detachment surgery. *Acta Ophthalmologica* 1993; **71**: 544–7. https://doi.org/10.1111/j.1755-3768.1993.tb04633.x.
301. Grasso A, Orsaria P, Costa F et al. Ultrasound-guided Interfascial Plane Blocks for Non-anesthesiologists in Breast Cancer Surgery: Functional Outcomes and Benefits. *Anticancer Research* 2020; **40**: 2231–8. https://doi.org/10.21873/anticanres.14185.
302. Gravino E, Griffo S, Gentile M, Storti M, Grossi N, Gily B. Comparison of two protocols of conscious analgosedation in video-assisted talc pleurodesis. *Minerva anestesiologica* 2005; **71**: 157–65.
303. Greiss M, Ghobrial BB, Elmageed WMA, Elfawy D, Mostafa R. Dexmedetomidine versus fentanyl on stress response and pain control in adult patients undergoing laparoscopic surgery. *Signa Vitae* 2022; **18**: 116-24. https://doi.org/10.22514/sv.2022.007.
304. Gultekin MH, Erdogan A, Akyol F. Evaluation of the Efficacy of the Erector Spinae Plane Block for Postoperative Pain in Patients Undergoing Percutaneous Nephrolithotomy: A Randomized Controlled Trial. *Journal of Endourology* 2020; **34**: 267–72. https://doi.org/10.1089/end.2019.0777.
305. Gunjan, Kohli M, Singh P, Gupta R. Multimodal versus Conventional Approach for Postoperative Pain Relief in Oral Cancer Patients. *Journal of Clinical and Diagnostic Research* 2016; **10**. https://doi.org/10.7860/JCDR/2016/13785.7027.
306. Guo M, Tang S, Wang Y et al. Comparison of intrathecal low-dose bupivacaine and morphine with intravenous patient control analgesia for postoperative analgesia for video-assisted thoracoscopic surgery. *BMC Anesthesiology* 2023; **23**: 395. https://doi.org/10.1186/s12871-023-02350-3.
307. Gupta L, Sinha S, Pande M, Vajifdar H. Ambulatory laparoscopic tubal ligation: a comparison of general anaesthesia with local anaesthesia and sedation. *J Anesthesiol Clin Pharmacology* 2011: 97–100.
308. Gupta R, Verma R, Bogra J, Kohli M, Raman R, Kushwaha J. A Comparative study of intrathecal dexmedetomidine and fentanyl as adjuvants to Bupivacaine. *Journal of Anaesthesiology Clinical Pharmacology* 2011; **27**: 339. https://doi.org/10.4103/0970-9185.83678.
309. Ha H-K, Lee K-G, Choi KK, Kim WS, Cho HR. Effect of bupivacaine on postoperative pain and analgesics use after single-incision laparoscopic appendectomy: double-blind randomized study. *Annals of Surgical Treatment and Research* 2020; **98**: 96. https://doi.org/10.4174/astr.2020.98.2.96.
310. Habib AMA, Zanaty OM, Anwer HF, Alia DA. The effect of paravertebral block on maxillofacial free flap survival. *International Journal of Oral and Maxillofacial Surgery* 2017; **46**: 706–11. https://doi.org/10.1016/j.ijom.2017.01.022.
311. Hadimioglu N, Ertug Z, Bigat Z, Yilmaz M, Yegin A. A Randomized Study Comparing Combined Spinal Epidural or General Anesthesia for Renal Transplant Surgery. *Transplantation Proceedings* 2005; **37**: 2020–2. https://doi.org/10.1016/j.transproceed.2005.03.034.
312. Hadzic A, Arliss J, Kerimoglu B et al. A Comparison of Infraclavicular Nerve Block versus General Anesthesia for Hand and Wrist Day-case Surgeries. *Anesthesiology* 2004; **101**: 127–32. https://doi.org/10.1097/00000542-200407000-00020.
313. Hadzic A, Karaca PE, Hobeika P et al. Peripheral Nerve Blocks Result in Superior Recovery Profile Compared with General Anesthesia in Outpatient Knee Arthroscopy. *Anesthesia & Analgesia* 2005; **100**: 976–81. https://doi.org/10.1213/01.ANE.0000150944.95158.B9.
314. Hallengren S, Schening A, Lindström A-C et al. Postoperative pain, recovery and discharge after robot‐assisted laparoscopic prostatectomy: A multicentre, single blinded, randomised controlled trial. *Acta Anaesthesiologica Scandinavica* 2024; **68**: 1006–15. https://doi.org/10.1111/aas.14465.
315. Hamed MA, Boules ML, Mahmoud MAEM, Abdelghaffar RA. The effect of erector spinae plane block on fentanyl consumption during open abdominal hysterectomy: a randomised controlled study. *BMC Anesthesiology* 2023; **23**: 194. https://doi.org/10.1186/s12871-023-02156-3.
316. Hamed R, Gamal L, Elsawy S, Baker MA, Abbas YH. Efficacy of ultrasound guided sphenopalatine ganglion block in management of emergence agitation after sinoscopic nasal surgery: a randomized double-blind controlled study. *Anaesthesia Critical Care & Pain Medicine* 2024; **43**: 101429. https://doi.org/10.1016/j.accpm.2024.101429.
317. Hammad MY, Elkholy M, Ebrahim ARA. Ultrasound Guided Continuous Quadratus Lumborum Block III for Postoperative Analgesia in The Percutaneous Nephrolithotomy: A Randomized Controlled Trial. *NeuroQuantology* 2022; **20**.
318. Handley GH, Silbert BS, Mooney PH, Schweitzer SA, Allen NB. Combined general and epidural anesthesia versus general anesthesia for major abdominal surgery: postanesthesia recovery characteristics. *Regional anesthesia* 1997; **22**: 435–41. https://doi.org/10.1016/s1098-7339(97)80030-2.
319. Hannibal K, Galatius H, Hansen A, Obel E, Ejlersen E. Preoperative Wound Infiltration with Bupivacaine Reduces Early and Late Opioid Requirement After Hysterectomy. *Anesthesia & Analgesia* 1996; **83**: 376–81. https://doi.org/10.1097/00000539-199608000-00030.
320. Hansen C, Dam M, Nielsen MV et al. Transmuscular quadratus lumborum block for total laparoscopic hysterectomy: a double-blind, randomized, placebo-controlled trial. *Regional Anesthesia & Pain Medicine* 2021; **46**: 25–30. https://doi.org/10.1136/rapm-2020-101931.
321. Hansen EG, Duedahl TH, Rømsing J, Hilsted K-L, Dahl JB. Intra‐operative remifentanil might influence pain levels in the immediate post‐operative period after major abdominal surgery. *Acta Anaesthesiologica Scandinavica* 2005; **49**: 1464–70. https://doi.org/10.1111/j.1399-6576.2005.00861.x.
322. Hanson NA, Derby RE, Auyong DB et al. Ultrasound-guided adductor canal block for arthroscopic medial meniscectomy: a randomized, double-blind trial. *Canadian Journal of Anesthesia/Journal canadien d’anesthésie* 2013; **60**: 874–80. https://doi.org/10.1007/s12630-013-9992-9.
323. Harsten A, Kehlet H, Ljung P, Toksvig‐Larsen S. Total intravenous general anaesthesia vs. spinal anaesthesia for total hip arthroplasty: a randomised, controlled trial. *Acta Anaesthesiologica Scandinavica* 2015; **59**: 298–309. https://doi.org/10.1111/aas.12456.
324. Harsten A, Kehlet H, Toksvig-Larsen S. Recovery after total intravenous general anaesthesia or spinal anaesthesia for total knee arthroplasty: a randomized trial. *British Journal of Anaesthesia* 2013; **111**: 391–9. https://doi.org/10.1093/bja/aet104.
325. Haruethaivijitchock P, Ng JL, Taksavanitcha G et al. Postoperative analgesic efficacy of modified continuous transversus abdominis plane block in laparoscopic colorectal surgery: a triple-blind randomized controlled trial. *Techniques in Coloproctology* 2020; **24**: 1179–87. https://doi.org/10.1007/s10151-020-02311-9.
326. Hassan RM, Hashim RM. Analgesic efficacy of ultrasound guided versus landmark-based bilateral superficial cervical plexus block for thyroid surgery. *Egyptian Journal of Anaesthesia* 2017; **33**: 365–73. https://doi.org/10.1016/j.egja.2017.07.002.
327. Hatipoglu Z, Gulec E, Turktan M et al. Comparative study of ultrasound-guided paravertebral block versus intravenous tramadol for postoperative pain control in percutaneous nephrolithotomy. *BMC Anesthesiology* 2018; **18**: 24. https://doi.org/10.1186/s12871-018-0479-7.
328. He Y, Huang M, Zhong Q, Ni H, Yu Z, Zhang X. Analgesic Effect of Ultrasound-Guided Anterior Quadratus Lumborum Block at the L2 Level in Patients Undergoing Laparoscopic Partial Nephrectomy: A Single-Center, Randomized Controlled Trial. *Pain Research and Management* 2022; **2022**: 1–8. https://doi.org/10.1155/2022/8958859.
329. Heid F, Jungbluth R, Schmitz J, Jage J. Intraoperative wound instillation of ropivacaine extraperitoneally subfascially fails to provide analgesia after remifentanil–isoflurane for abdominal hysterectomy. *Acute Pain* 2005; **7**: 133–7. https://doi.org/10.1016/j.acpain.2005.08.003.
330. Heidari SM, Soltani H, Hashemi SJ, Talakoub R, Soleimani B. Comparative study of two anesthesia methods according to postoperative complications and one month mortality rate in the candidates of hip surgery. *Journal of research in medical sciences : the official journal of Isfahan University of Medical Sciences* 2011; **16**: 323–30.
331. Helvacioglu A, Weis R. Operative laparoscopy and postoperative pain relief. *Fertility and sterility* 1992; **57**: 548–52.
332. Herrador-Benito J, Páramo-Zunzunegui J, Rodríguez-Caravaca G, Durán-Poveda M. Pre-incisional local infiltration with levobupivacaine in laparoscopic cholecystectomy: a randomized and clinical trial. *Cirugía y Cirujanos* 2024; **92**. https://doi.org/10.24875/CIRU.230000221.
333. Hojski A, Krämer M, Gecas P et al. The efficacy of loco-regional ropivacaine analgesia via intercostal catheters after lung resection: a randomized, double-blind, placebo-controlled, superiority study. *European Journal of Cardio-Thoracic Surgery* 2024; **66**. https://doi.org/10.1093/ejcts/ezae342.
334. Hong J-Y, Yang SC, Yi J, Kil HK. Epidural ropivacaine and sufentanil and the perioperative stress response after a radical retropubic prostatectomy. *Acta anaesthesiologica Scandinavica* 2011; **55**: 282–9. https://doi.org/10.1111/j.1399-6576.2010.02360.x.
335. Hosgood SA, Thiyagarajan UM, Nicholson HFL, Jeyapalan I, Nicholson ML. Randomized Clinical Trial of Transversus Abdominis Plane Block Versus Placebo Control in Live-Donor Nephrectomy. *Transplantation* 2012; **94**: 520–5. https://doi.org/10.1097/TP.0b013e31825c1697.
336. Hosseinpour M, Behdad A, Resaei M. Assessment of the effect of local versus general anesthesia on the pain perception after inguinal hernia surgery. *Ambulatory Surgery* 2013: 10–2.
337. Hu J, Chen Q, Xu Q, Song Y, Wei K, Lei X. Analgesic effect of ultrasound-guided erector spinae plane block (espb) in general anesthesia for cesarean section: a randomized controlled trial. *BMC Anesthesiology* 2022; **22**: 244. https://doi.org/10.1186/s12871-022-01781-8.
338. Hu Z, Zhou Y, Zhao G et al. Effects of quadratus lumborum block on perioperative multimodal analgesia and postoperative outcomes in patients undergoing radical prostatectomy. *BMC Anesthesiology* 2022; **22**: 213. https://doi.org/10.1186/s12871-022-01755-w.
339. Huda AU, Alshahrani AS, Yasir M, Sawilah A, Alharthi AAN. Erector spinae block reduces intraoperative and postoperative opioid consumption in patients undergoing laparoscopic sleeve gastrectomy: A randomized controlled trial. *Qatar medical journal* 2024; **2024**: 58. https://doi.org/10.5339/qmj.2024.58.
340. Hunt W, Nath M, Bowrey S, Colvin L, Thompson JP. Effect of a continuous perineural levobupivacaine infusion on pain after major lower limb amputation: a randomised double-blind placebo-controlled trial. *BMJ Open* 2023; **13**: e060349. https://doi.org/10.1136/bmjopen-2021-060349.
341. Hwang B-Y, Kim E, Kwon J et al. The analgesic efficacy of a single injection of ultrasound-guided retrolaminar paravertebral block for breast surgery: a prospective, randomized, double-blinded study. *The Korean Journal of Pain* 2020; **33**: 378–85. https://doi.org/10.3344/kjp.2020.33.4.378.
342. Hwang W, Lee J, Park J, Joo J. Dexmedetomidine versus remifentanil in postoperative pain control after spinal surgery: a randomized controlled study. *BMC Anesthesiology* 2015; **15**: 21. https://doi.org/10.1186/s12871-015-0004-1.
343. Ibrahim AS, Farrag WS, Aly MG. Ultrasound guided bilateral rectus sheath block and serum TNF-α and IL-6 after radical prostatectomy: A randomized double blinded study. *Egyptian Journal of Anaesthesia* 2018; **34**: 139–43. https://doi.org/10.1016/j.egja.2018.07.003.
344. Ishio J, Komasawa N, Kido H, Minami T. Evaluation of ultrasound-guided posterior quadratus lumborum block for postoperative analgesia after laparoscopic gynecologic surgery. *Journal of Clinical Anesthesia* 2017; **41**: 1–4. https://doi.org/10.1016/j.jclinane.2017.05.015.
345. Ismail MT, Elshmaa NS. Pre-emptive analgesia by nerve stimulator guided pudendal nerve block for posterior colpoperineorrhaphy. *European Journal of Obstetrics & Gynecology and Reproductive Biology* 2012; **163**: 200–3. https://doi.org/10.1016/j.ejogrb.2012.03.032.
346. Jabalameli M, Rouholamin S, Gourtanian F. A comparison of the effects of fentanyl and remifentanil on nausea, vomiting, and pain after cesarean section. *Iranian journal of medical sciences* 2011; **36**: 183–7.
347. Jæger P, Grevstad U, Henningsen MH, Gottschau B, Mathiesen O, Dahl JB. Effect of adductor‐canal‐blockade on established, severe post‐operative pain after total knee arthroplasty: a randomised study. *Acta Anaesthesiologica Scandinavica* 2012; **56**: 1013–9. https://doi.org/10.1111/j.1399-6576.2012.02737.x.
348. Jahanbakhsh S, Bameshki A, Khashayar P. Remifentanil-Induced Abdominal Pain: A Randomised Clinical Trial. *Anaesthesia and Intensive Care* 2009; **37**: 447–9. https://doi.org/10.1177/0310057X0903700302.
349. Jamnig D, Kapral S, Urak G et al. Addition of fentanyl to mepivacaine does not affect the duration of brachial plexus block. *Acute Pain* 2003; **5**: 51–6. https://doi.org/10.1016/S1366-0071(03)00027-5.
350. Jankowski CJ, Hebl JR, Stuart MJ et al. A Comparison of Psoas Compartment Block and Spinal and General Anesthesia for Outpatient Knee Arthroscopy. *Anesthesia & Analgesia* 2003; **97**: 1003–9. https://doi.org/10.1213/01.ANE.0000081798.89853.E7.
351. Janssen H, Stosch R von, Pöschl R et al. Blood pressure response to combined general anaesthesia/interscalene brachial plexus block for outpatient shoulder arthroscopy. *BMC Anesthesiology* 2014; **14**: 50. https://doi.org/10.1186/1471-2253-14-50.
352. Jellish SW, Sheikh T, Baker WH, Louie EK, Slogoff S. Hemodynamic Stability, Myocardial Ischemia, and Perioperative Outcome After Carotid Surgery with Remifentanil/Propofol or Isoflurane/Fentanyl Anesthesia. *Journal of Neurosurgical Anesthesiology* 2003; **15**: 176–84. https://doi.org/10.1097/00008506-200307000-00004.
353. Jellish WS, Thalji Z, Stevenson K, Shea J. A Prospective Randomized Study Comparing Short- and Intermediate-Term Perioperative Outcome Variables After Spinal or General Anesthesia for Lumbar Disk and Laminectomy Surgery. *Anesthesia & Analgesia* 1996; **83**: 559–64. https://doi.org/10.1097/00000539-199609000-00021.
354. Jeong H, Jeong S, Lim HJ, Lee J, Yoo KY. Cerebral Oxygen Saturation Measured by Near-infrared Spectroscopy and Jugular Venous Bulb Oxygen Saturation during Arthroscopic Shoulder Surgery in Beach Chair Position under Sevoflurane-Nitrous Oxide or Propofol-Remifentanil Anesthesia. *Anesthesiology* 2012; **116**: 1047–56. https://doi.org/10.1097/ALN.0b013e31825154d2.
355. Jessen C, Brix LD, Nielsen TD, Espelund US, Lund B, Bendtsen TF. Efficacy of iliopsoas plane block for patients undergoing hip arthroscopy: a prospective, triple-blind, randomized, placebo-controlled trial. *Regional Anesthesia & Pain Medicine* 2025; **50**: 5–10. https://doi.org/10.1136/rapm-2023-104989.
356. Jiang F, Wu A, Liang Y et al. Assessment of Ultrasound-Guided Continuous Low Serratus Anterior Plane Block for Pain Management After Hepatectomy: A Randomized Controlled Trial. *Journal of Pain Research* 2023; **16**: 2383–92. https://doi.org/10.2147/JPR.S406498.
357. Jiang L, Wang C, Tong J, Han X, Miao C, Liang C. Comparison between thoracic epidural analgesia VS patient controlled analgesia on chronic postoperative pain after video-assisted thoracoscopic surgery: A prospective randomized controlled study. *Journal of Clinical Anesthesia* 2025; **100**: 111685. https://doi.org/10.1016/j.jclinane.2024.111685.
358. Jin L, Yu Y, Miao P, Huang Y, Yu S, Guo K. Effect of Continuous Erector Spinae Plane Block on Postoperative Recovery in Patients Undergoing Minimally Invasive Cardiac Surgery: A Prospective, Randomized Controlled Clinical Trial. *Current Medical Science* 2024; **44**: 1103–12. https://doi.org/10.1007/s11596-024-2593-4.
359. Jin Y, Zhao S, Cai J et al. Erector Spinae Plane Block for Perioperative Pain Control and Short-term Outcomes in Lumbar Laminoplasty: A Randomized Clinical Trial. *Journal of Pain Research* 2021; **14**: 2717–27. https://doi.org/10.2147/JPR.S321514.
360. Jinaworn P, Pannangpetch P, Bunanantanasan K et al. Efficacy of Erector Spinae Plane Block on Postoperative Analgesia for Patients Undergoing Metabolic Bariatric Surgery: A Randomized Controlled Trial. *Obesity Surgery* 2024; **34**: 4211–9. https://doi.org/10.1007/s11695-024-07515-8.
361. Johansen M, Harbo G, Ilium P. Preincisional Infiltration With Bupivacaine in Tonsillectomy. *Archives of Otolaryngology - Head and Neck Surgery* 1996; **122**: 261–3. https://doi.org/10.1001/archotol.1996.01890150039007.
362. Johansson A, Kornfält J, Nordin L, Svensson L, Ingvar C, Lundberg J. Wound infiltration with ropivacaine and fentanyl: effects on postoperative pain and PONV after breast surgery. *Journal of Clinical Anesthesia* 2003; **15**: 113–8. https://doi.org/10.1016/S0952-8180(02)00511-1.
363. Jonnavithula N, Khandelia H, Durga P, Ramachandran G. Role of wound instillation with bupivacaine through surgical drains for postoperative analgesia in modified radical mastectomy. *Indian journal of anaesthesia* 2015; **59**: 15–20. https://doi.org/10.4103/0019-5049.149443.
364. Jørgensen H, Fomsgaard JS, Dirks J, Wetterslev J, Andreasson B, Dahl JB. Effect of peri‐ and postoperative epidural anaesthesia on pain and gastrointestinal function after abdominal hysterectomy. *British Journal of Anaesthesia* 2001; **87**: 577–83. https://doi.org/10.1093/bja/87.4.577.
365. Jose DE, Mathew LM, Koshy I, Mathew A, Ganapathi P. Comparison of General Anaesthesia and Epidural Anaesthesia in Lumbar Microdiscectomies- A Prospective Comparative Study. *Journal of Clinical and Diagnostic Research* 2023. **17**: 44-8. https://doi.org/10.7860/JCDR/2023/60334.18087.
366. Joshi GP, Warner DS, Twersky RS, Fleisher LA. A comparison of the remifentanil and fentanyl adverse effect profile in a multicenter phase IV study. *Journal of Clinical Anesthesia* 2002; **14**: 494–9. https://doi.org/10.1016/S0952-8180(02)00404-X.
367. Jung H-S, Chun K-J, Kim JY, Lee J, Lee JS. Does Surgical-site Multimodal Drug Injection After Palmar Plating of Distal Radius Fractures Improve Pain Scores? *Clinical Orthopaedics & Related Research* 2020; **478**: 2663–9. https://doi.org/10.1097/CORR.0000000000001212.
368. Kahveci K, Doger C, Ornek D, Gokcinar D, Aydemir S, Ozay R. Perioperative outcome and cost-effectiveness of spinal versus general anesthesia for lumbar spine surgery. *Neurologia i Neurochirurgia Polska* 2014; **48**: 167–73. https://doi.org/10.1016/j.pjnns.2014.05.005.
369. Kairaluoma PM, Bachmann MS, Korpinen AK, Rosenberg PH, Pere PJ. Single-Injection Paravertebral Block Before General Anesthesia Enhances Analgesia After Breast Cancer Surgery With and Without Associated Lymph Node Biopsy. *Anesthesia & Analgesia* 2004; **99**: 1837–43. https://doi.org/10.1213/01.ANE.0000136775.15566.87.
370. Kakagia DD, Fotiadis S, Tripsiannis G, Tsoutsos D. Postoperative Analgesic Effect of Locally Infiltrated Levobupivacaine in Fleur-de-Lys Abdominoplasty. *Aesthetic Plastic Surgery* 2007; **31**: 128–32. https://doi.org/10.1007/s00266-006-0187-4.
371. Kamel AAF, Fahmy AM, Medhat MM, Elmesallamy WAEA, Salem DAE. Retrolaminar block for opioid-free anaesthesia and enhanced recovery after posterior lumbar discectomy: A randomised controlled study. *Indian Journal of Anaesthesia* 2024; **68**: 261–6. https://doi.org/10.4103/ija.ija_773_23.
372. Kamiya Y, Hasegawa M, Yoshida T, Takamatsu M, Koyama Y. Impact of pectoral nerve block on postoperative pain and quality of recovery in patients undergoing breast cancer surgery. *European Journal of Anaesthesiology* 2018; **35**: 215–23. https://doi.org/10.1097/EJA.0000000000000762.
373. Kanaujia SK, Yadav AK, Chaurasiya MK, Naithani B, Chaudhary A. Comparative Evaluation of Dexmedetomidine and Butorphanol on Perioperative Hemodynamics, Sedation and Postoperative Analgesia for Patients Undergoing Lower Abdominal Surgery. *International Journal of Pharmaceutical and Clinical Research* 2024; **16**: 403-13
374. Kang K, Meng X, Li B et al. Effect of thoracic paravertebral nerve block on the early postoperative rehabilitation in patients undergoing thoracoscopic radical lung cancer surgery. *World Journal of Surgical Oncology* 2020; **18**: 298. https://doi.org/10.1186/s12957-020-02071-8.
375. Kang KH, Kim BS, Kang H. The benefits of preincision ropivacaine infiltration for reducing postoperative pain after robotic bilateral axillo-breast approach thyroidectomy: a prospective, randomized, double-blind, placebo-controlled study. *Annals of surgical treatment and research* 2015; **88**: 193–9. https://doi.org/10.4174/astr.2015.88.4.193.
376. Kannan Sudeesh, Surhonne Netra S, R Chetan Kumar, B Kavitha, D Devika Rani, S R Raghavendra Rao. Effects of bilateral superficial cervical plexus block on sevoflurane consumption during thyroid surgery under entropy-guided general anesthesia: a prospective randomized study. *Korean Journal of Anesthesiology* 2018; **71**: 141–8. https://doi.org/10.4097/kjae.2018.71.2.141.
377. Karacalar S, Bilen CY, Sarihasan B, Sarikaya S. Spinal-Epidural Anesthesia Versus General Anesthesia in the Management of Percutaneous Nephrolithotripsy. *Journal of Endourology* 2009; **23**: 1591–7. https://doi.org/10.1089/end.2009.0224.
378. Karakış A, Tapar H, Özsoy Z et al. Eficácia analgésica perioperatória do bloqueio bilateral do plexo cervical superficial em pacientes submetidos à tireoidectomia: estudo clínico randomizado. *Brazilian Journal of Anesthesiology* 2019; **69**: 455–60. https://doi.org/10.1016/j.bjan.2019.06.013.
379. Karaman S, Kocabas S, Uyar M, Zincircioglu C, Firat V. Intrathecal morphine: Effects on perioperative hemodynamics, postoperative analgesia, and stress response for total abdominal hysterectomy. *Advances in Therapy* 2006; **23**: 295–306. https://doi.org/10.1007/BF02850135.
380. Karaman T, Ozsoy AZ, Karaman S et al. Efeitos do bloqueio do plano transverso abdominal sobre o consumo de analgésico e anestésico durante histerectomia abdominal total: um estudo randômico e controlado. *Brazilian Journal of Anesthesiology* 2018; **68**: 285–91. https://doi.org/10.1016/j.bjan.2017.12.005.
381. Karaveli A, Kaplan S, Kavakli AS, Kosar MN, Mayir B. The Effect of Ultrasound-Guided Erector Spinae Plane Block on Postoperative Opioid Consumption and Respiratory Recovery in Laparoscopic Sleeve Gastrectomy: A Randomized Controlled Study. *Obesity Surgery* 2025; **35**: 112–21. https://doi.org/10.1007/s11695-024-07576-9.
382. Kartalov A, Jankulovski N, Kuzmanovska B et al. The Effect of Rectus Sheath Block as a Supplement of General Anesthesia on Postoperative Analgesia in Adult Patient Undergoing Umbilical Hernia Repair. *PRILOZI* 2017; **38**: 135–42. https://doi.org/10.2478/prilozi-2018-0014.
383. Karthikeyan VS, Sistla SC, Badhe AS et al. Randomized Controlled Trial on the Efficacy of Bilateral Superficial Cervical Plexus Block in Thyroidectomy. *Pain Practice* 2013; **13**: 539–46. https://doi.org/10.1111/papr.12022.
384. Katar MK, Turan UF. Efficacy and Safety of Paragastric Neural Blockade in Controlling Pain, Nausea, and Vomiting After Sleeve Gastrectomy: A Randomized Controlled Trial. *Obesity Surgery* 2024; **34**: 2383–90. https://doi.org/10.1007/s11695-024-07255-9.
385. Katatbeh MA, Khasawneh L, Al-Mashakbeh Y et al. Role of pre-incisional external bilateral ultrasound guide glossopharyngeal nerve block with diluted bupivacaine mixture in pain management in oropharyngeal surgery: A randomized control trial. *American Journal of Otolaryngology* 2022; **43**: 103468. https://doi.org/10.1016/j.amjoto.2022.103468.
386. Katz J, Clairoux M, Redahan C et al. High dose alfentanil pre-empts pain after abdominal hysterectomy. *Pain* 1996; **68**: 109–18. https://doi.org/10.1016/S0304-3959(96)03172-7.
387. Katz J, Cohen L, Schmid R, Chan VWS, Wowk A. Postoperative morphine use and hyperalgesia are reduced by preoperative but not intraoperative epidural analgesia: implications for preemptive analgesia and the prevention of central sensitization. *Anesthesiology* 2003; **98**: 1449–60. https://doi.org/10.1097/00000542-200306000-00023.
388. Kaufman Y, Hirsch I, Ostrovsky L et al. Pain Relief by Continuous Intraperitoneal Nebulization of Ropivacaine during Gynecologic Laparoscopic Surgery–A Randomized Study and Review of the Literature. *Journal of Minimally Invasive Gynecology* 2008; **15**: 554–8. https://doi.org/10.1016/j.jmig.2008.05.012.
389. Kaur U, Shamshery C, Agarwal A, Prakash N, Valiveru RC, Mishra P. Evaluation of postoperative pain in patients undergoing modified radical mastectomy with pectoralis or serratus-intercostal fascial plane blocks. *Korean Journal of Anesthesiology* 2020; **73**: 425–33. https://doi.org/10.4097/kja.20159.
390. Kausalya R, Jacob R. Efficacy of Low-Dose Epidural Anaesthesia in Surgery of the Anal Canal—A Randomised Controlled Trial. *Anaesthesia and Intensive Care* 1994; **22**: 161–4. https://doi.org/10.1177/0310057X9402200207.
391. Kavakli AS, Sahin T, Koc U, Karaveli A. Ultrasound-Guided External Oblique Intercostal Plane Block for Postoperative Analgesia in Laparoscopic Sleeve Gastrectomy: A Prospective, Randomized, Controlled, Patient and Observer-Blinded Study. *Obesity Surgery* 2024; **34**: 1505–12. https://doi.org/10.1007/s11695-024-07174-9.
392. Kavanagh BP, Katz J, Sandler AN et al. Multimodal analgesia before thoracic surgery does not reduce postoperative pain. *British journal of anaesthesia* 1994; **73**: 184–9. https://doi.org/10.1093/bja/73.2.184.
393. Kawahara R, Tamai Y, Yamasaki K, Okuno S, Hanada R, Funato T. The analgesic efficacy of ultrasound-guided transversus abdominis plane block with mid-axillary approach after gynecologic laparoscopic surgery: A randomized controlled trial. *Journal of anaesthesiology, clinical pharmacology* 2015; **31**: 67–71. https://doi.org/10.4103/0970-9185.150547.
394. Kaya M, Oğuz G, Şenel G, Kadıoğulları N. Postoperative analgesia after modified radical mastectomy: the efficacy of interscalene brachial plexus block. *Journal of Anesthesia* 2013; **27**: 862–7. https://doi.org/10.1007/s00540-013-1647-z.
395. Kejriwal R, Cooper J, Legg A, Stanley J, Rosenfeldt MP, Walsh SJ. Efficacy of the Adductor Canal Approach to Saphenous Nerve Block for Anterior Cruciate Ligament Reconstruction With Hamstring Autograft: A Randomized Controlled Trial. *Orthopaedic journal of sports medicine* 2018; **6**: 2325967118800948. https://doi.org/10.1177/2325967118800948.
396. Kekul O, Ustun YB, Kaya C et al. Analgesic efficacy of the bilateral erector spinae plane block for colorectal surgery: a randomized controlled trial. *Journal of anesthesia, analgesia and critical care* 2022; **2**: 43. https://doi.org/10.1186/s44158-022-00073-4.
397. Keller DS, Ermlich BO, Schiltz N et al. The Effect of Transversus Abdominis Plane Blocks on Postoperative Pain in Laparoscopic Colorectal Surgery. *Diseases of the Colon & Rectum* 2014; **57**: 1290–7. https://doi.org/10.1097/DCR.0000000000000211.
398. Keller M, Dinkel F, Jacoby J et al. Oblique subcostal transverse abdominis plane block for postoperative pain control in patients undergoing open sublay mesh hernia repair: a prospective double-blind randomized placebo-controlled clinical trial. *Regional Anesthesia & Pain Medicine* 2024: rapm-2024-105596. https://doi.org/10.1136/rapm-2024-105596.
399. Kesimci E, Öztürk L, Bercin S, Kırış M, Eldem A, Kanbak O. Role of sphenopalatine ganglion block for postoperative analgesia after functional endoscopic sinus surgery. *European Archives of Oto-Rhino-Laryngology* 2012; **269**: 165–9. https://doi.org/10.1007/s00405-011-1702-z.
400. Ketata S, Maktouf Y, Zouche I et al. Intravenous versus intracuff alkalinized lidocaine to prevent postoperative sore throat: a prospective randomized controlled trial. *The Pan African Medical Journal* 2024; **48**. https://doi.org/10.11604/pamj.2024.48.18.40317.
401. Khajavi MR, Asadian MA, Imani F, Etezadi F, Moharari RS, Amirjamshidi A. General anesthesia versus combined epidural/general anesthesia for elective lumbar spine disc surgery: A randomized clinical trial comparing the impact of the two methods upon the outcome variables. *Surgical neurology international* 2013; **4**: 105. https://doi.org/10.4103/2152-7806.116683.
402. Khalil I, Itani SE, Naja Z et al. Nerve stimulator–guided pudendal nerve block vs general anesthesia for postoperative pain management after anterior and posterior vaginal wall repair: a prospective randomized trial. *Journal of Clinical Anesthesia* 2016; **34**: 668–75. https://doi.org/10.1016/j.jclinane.2016.07.024.
403. Kilbasanli S, Kaçmaz M. General anesthesia versus combined interscalene nerve/superficial cervical plexus block in arthroscopic rotator cuff repair: A randomized prospective control trial. *Medicine* 2023; **102**: e35522. https://doi.org/10.1097/MD.0000000000035522.
404. Kim JY, Lee JS, Kim JY et al. Iliopsoas plane block does not improve pain after primary total hip arthroplasty in the presence of multimodal analgesia: a single institution randomized controlled trial. *Regional Anesthesia & Pain Medicine* 2025; **50**: 257–63. https://doi.org/10.1136/rapm-2023-105092.
405. Kim JS, Choi JB, Lee SY et al. Pain related to robotic cholecystectomy with lower abdominal ports. *Medicine* 2016; **95**: e4445. https://doi.org/10.1097/MD.0000000000004445.
406. Kim J-S, Lee J, Soh E-Y et al. Analgesic Effects of Ultrasound-Guided Serratus-Intercostal Plane Block and Ultrasound-Guided Intermediate Cervical Plexus Block After Single-Incision Transaxillary Robotic Thyroidectomy. *Regional Anesthesia and Pain Medicine* 2016; **41**: 584–8. https://doi.org/10.1097/AAP.0000000000000430.
407. Kim M-S, Kim B-H, Han YE, Nam DW, Hah JH. Clinical outcomes after local anesthesia with monitored anesthesia care during thyroidectomy and selective neck dissection: a randomized study. *European Archives of Oto-Rhino-Laryngology* 2017; **274**: 3789–94. https://doi.org/10.1007/s00405-017-4707-4.
408. Kim MG, Kim SI, Ok SY et al. The analgesic effect of ultrasound-guided transverse abdominis plane block after laparoscopic totally extraperitoneal hernia repair. *Korean journal of anesthesiology* 2012; **63**: 227–32. https://doi.org/10.4097/kjae.2012.63.3.227.
409. Kim MG, Kim SI, Ok SY et al. Is transverse abdominis plane block effective following local anesthetic infiltration in laparoscopic totally extraperitoneal hernia repair? *Korean Journal of Anesthesiology* 2014; **67**: 398. https://doi.org/10.4097/kjae.2014.67.6.398.
410. Kim Y, Bae H, Yoo S et al. Effect of remifentanil on post-operative analgesic consumption in patients undergoing shoulder arthroplasty after interscalene brachial plexus block: a randomized controlled trial. *Journal of Anesthesia* 2022; **36**: 506–13. https://doi.org/10.1007/s00540-022-03085-0.
411. Kir MC, Kir G. Ankle Nerve Block Adjuvant to General Anesthesia Reduces Postsurgical Pain and Improves Functional Outcomes in Hallux Valgus Surgery. *Medical Principles and Practice* 2018; **27**: 236–40. https://doi.org/10.1159/000488321.
412. Kiribayashi M, Inagaki Y, Nishimura Y, Yamasaki K, Takahashi S, Ueda K. Caudal blockade shortens the time to walking exercise in elderly patients following low back surgery. *Journal of Anesthesia* 2010; **24**: 192–6. https://doi.org/10.1007/s00540-009-0840-6.
413. Kita T, Maki N, Song YS, Arai F, Nakai T. Caudal epidural anesthesia administered intraoperatively provides for effective postoperative analgesia after total hip arthroplasty. *Journal of Clinical Anesthesia* 2007; **19**: 204–8. https://doi.org/10.1016/j.jclinane.2006.10.011.
414. Klaibert B, Lohser J, Tang R, Jew M, McGuire A, Wilson J. Efficacy of ultrasound-guided single-injection erector spinae plane block for thoracoscopic wedge resection: a prospective randomized control trial. *Regional Anesthesia & Pain Medicine* 2022; **47**: 749–54. https://doi.org/10.1136/rapm-2022-103602.
415. Kock MD, Lavand’homme P, Waterloos H. The Short-Lasting Analgesia and Long-Term Antihyperalgesic Effect of Intrathecal Clonidine in Patients Undergoing Colonic Surgery. *Anesthesia & Analgesia* 2005; **101**: 566–72. https://doi.org/10.1213/01.ANE.0000157121.71808.04.
416. Kodali VRK, Shree S, Prasad M, Sambandam KKG, Karthekeyan RB, Vakamudi M. A Comparative Study of Bilateral Erector Spinae Block Versus Intravenous Dexmedetomidine for Perioperative Pain Management in Patients Undergoing Off-Pump Coronary Artery Bypass Grafting - A Single-Blind Randomized Controlled Trial. *Journal of Cardiothoracic and Vascular Anesthesia* 2022; **36**: 4085–92. https://doi.org/10.1053/j.jvca.2022.07.015.
417. Kolsi K, Ghozzi H, Masmoudi A et al. Intraperitoneal lignocaine for analgesia after laparoscopic cholecystectomy. *Acute Pain* 2000; **3**: 200–5. https://doi.org/10.1016/S1366-0071(00)90005-6.
418. Korhonen A-M, Valanne JV, Jokela RM, Ravaska P, Korttila KT. A Comparison of Selective Spinal Anesthesia with Hyperbaric Bupivacaine and General Anesthesia with Desflurane for Outpatient Knee Arthroscopy. *Anesthesia & Analgesia* 2004: 1668–73. https://doi.org/10.1213/01.ANE.0000139351.40608.05.
419. Köroğlu S, Takmaz SA, Kaymak C, Narli A, Karalezli K, Dikmen B. The preoperative analgesic effect of 3-in-1 block on postoperative pain and tramadol consumption in total hip arthroplasty. *Agri : Agri (Algoloji) Dernegi’nin Yayin organidir = The journal of the Turkish Society of Algology* 2008; **20**: 19–25.
420. Koshchak E, Qian D, Fang S et al. EP176 Erector spinae plane block vs. pecto-intercostal fascial plane block vs. control for sternotomy: a prospective randomized trial. *ePoster Session 5 – Station 6*. BMJ Publishing Group Ltd, 2023: A135.2-A136.
421. Kovac AL, Azad SS, Steer P, Witkowski T, Batenhorst R, McNeal S. Remifentanil versus alfentanil in a balanced anesthetic technique for total abdominal hysterectomy. *Journal of Clinical Anesthesia* 1997; **9**: 532–41. https://doi.org/10.1016/S0952-8180(97)00140-2.
422. Kratz T, Dette F, Schmitt J, Wiesmann T, Wulf H, Zoremba M. Impact of regional femoral nerve block during general anesthesia for hip arthoplasty on blood pressure, heart rate and pain control: A randomized controlled study. *Technology and Health Care* 2015; **23**: 313–22. https://doi.org/10.3233/THC-150898.
423. Kristin N. Vitreoretinal surgery: pre-emptive analgesia. *British Journal of Ophthalmology* 2001; **85**: 1328–31. https://doi.org/10.1136/bjo.85.11.1328.
424. Krobot R, Premuzic J. Comparison of general and spinal anaesthesia in patients undergoing open ventral hernia repair. *Periodicum Biologurum* 2013; **115**: 225–9.
425. Kuju RB, Dongol Y, Verma R. Effectiveness of Spinal Anaesthesia versus General Anaesthesia for Open Cholecystectomy. *Journal of Nepal Health Research Council* 2016; **14**: 93–8.
426. Kumar A, Kumar V, Kumar P, Ranjan V, Verma VK. Comparison of Efficacy of Bupivacaine Plus Tramadol with Bupivacaine Plus Dexmedetomidine as Preincisional Infiltration in Patients Undergoing Abdominal Surgery Under General Anasthesia: A Prospective Randomised Double Blinding Study. *International Journal of Current Pharmaceutical Review and Research* 2023; **15**: 146-52.
427. Kumar A, Sinha C, Kumar A, Kumari P, Mukul S. Ultrasound-guided trigeminal nerve block and its comparison with conventional analgesics in patients undergoing faciomaxillary surgery: Randomised control trial. *Indian Journal of Anaesthesia* 2018; **62**: 871. https://doi.org/10.4103/ija.IJA_256_18.
428. Kumar KN, Kalyane RN, Singh NG et al. Efficacy of Bilateral Pectoralis Nerve Block for Ultrafast Tracking and Postoperative Pain Management in Cardiac Surgery. *Annals of Cardiac Anaesthesia* 2018; **21**: 333–8. https://doi.org/10.4103/aca.ACA_15_18.
429. Kumar N, Thapa D, Gombar S, Ahuja V, Gupta R. Analgesic efficacy of pre‐operative stellate ganglion block on postoperative pain relief: a randomised controlled trial. *Anaesthesia* 2014; **69**: 954–60. https://doi.org/10.1111/anae.12774.
430. Kumar P, Singh A, Sharma J, Parshad S, Johar S, Kaur K. Assessment of ultrasound guided erector spinae plane block for early post-operative analgesia for modified radical mastectomy: a prospective, randomized, controlled study. *Medical Gas Research* 2024; **14**: 201–5. https://doi.org/10.4103/mgr.mgr_74_20.
431. Kumar P, Kumar R, Harshwardhan, Kumari S. A Prospective Randomised Study Comparing Postoperative Outcome after Regional or General Anesthesia for Incisional Hernia Surgery in Obese Patients. *International Journal of Pharmaceutical and Clinical Research* 2013; **15**: 44–51.
432. Kumar S, Goel D, Sharma S et al. A randomised controlled study of the post-operative analgesic efficacy of ultrasound-guided pectoral nerve block in the first 24 h after modified radical mastectomy. *Indian Journal of Anaesthesia* 2018; **62**: 436. https://doi.org/10.4103/ija.IJA_523_17.
433. Kumar S, Palaniappan JM, Kishan A. Preemptive Caudal Ropivacaine: An Effective Analgesic during Degenerative Lumbar Spine Surgery. *Asian spine journal* 2017; **11**: 113–9. https://doi.org/10.4184/asj.2017.11.1.113.
434. Kumari M, Meena S, Patel SR. A HOSPITAL BASED PROSPECTIVE STUDY TO ASSESS THE COMPARISON OF COMBINED EPIDURAL & GENERAL ANESTHESIA VERSUS GENERAL ANESTHESIA FOR LAPAROSCOPIC CHOLECYSTECTOMY AT TERTIARY CARE CENTER. *Journal of Clinical and Diagnostic Research* 2025; **6**: 729-33. https://doi.org/10.47009/jamp.2024.6.3.150.
435. Kuo CP, Jao SW, Chen KM et al. Comparison of the effects of thoracic epidural analgesia and i.v. infusion with lidocaine on cytokine response, postoperative pain and bowel function in patients undergoing colonic surgery. *British Journal of Anaesthesia* 2006; **97**: 640–6. https://doi.org/10.1093/bja/ael217.
436. Kurtoğlu M, Ateş S, Bakkaloğlu B et al. Epidural anesthesia versus general anesthesia in patients undergoing minimally invasive direct coronary artery bypass surgery. *Anadolu kardiyoloji dergisi : AKD = the Anatolian journal of cardiology* 2009; **9**: 54–8.
437. Kuş A, Yörükoğlu UH, Aksu C, Çınar S, Cantürk NZ, Gürkan Y. Efeito do bloqueio paravertebral torácico na redução de seroma em cirurgia de mama – estudo randomizado controlado. *Brazilian Journal of Anesthesiology* 2020; **70**: 215–9. https://doi.org/10.1016/j.bjan.2019.12.015.
438. Kusderci H, Tulgar S, Genc C et al. EP125 Evaluation of ultrasound-guided external oblique intercostal plane block for postoperative analgesia in laparoscopic cholecystectomy: A prospective, randomized, controlled clinical trial. *ePoster Session 4 – Station 3*. BMJ Publishing Group Ltd, 2023; **48**: A106.2-A107. https://doi.org/1 0.1136/rapm-2023-ESRA.187.
439. Kwon O, Lee J-M, Park J et al. Influence of anesthesia methods on surgical outcomes and renal function in retrograde intrarenal stone surgery: a prospective, randomized controlled study. *BMC anesthesiology* 2019; **19**: 239. https://doi.org/10.1186/s12871-019-0901-9.
440. Kwon WK, Choi JW, Kang JE et al. Long Thoracic Nerve Block in Video-Assisted Thoracoscopic Wedge Resection for Pneumothorax. *Anaesthesia and Intensive Care* 2012; **40**: 773–9. https://doi.org/10.1177/0310057X1204000504.
441. L N, B AK, Brindha R, S S. Impact of Dexmedetomidine and Fentanyl on Analgesic Efficacy and Side Effects in Post-Laparoscopic Vaginal Hysterectomy: A Comparison with Intraperitoneal Ropivacaine. *International Journal of Pharmaceutical Quality Assurance* 2024; **15**: 327–30. https://doi.org/10.25258/ijpqa.15.1.51.
442. Lai R, Luo Q, Lai J, Lu X, Xu M. Ultrasound-guided quadratus lumborum block for perioperative analgesia in robot-assisted partial nephrectomy: a randomized controlled trial. *Trials* 2021; **22**: 840. https://doi.org/10.1186/s13063-021-05815-3.
443. Lam ND, Khanh ND, Anh ND. Research Article Evaluating the pain relief effects after caesarean delivery by ultrasound-guided transversus abdominis plane (tap) block in Hanoi Obstetrics and Gynecology Hospital, Vietnam. *Genetics and Molecular Research* 2021; **20**. https://doi.org/10.4238/gmr18739.
444. Lech J, Bryk M, Jasek P, Kabała MM. The assessment of haemodynamic stability, gas exchange parameters, and the quality of postoperative analgesia in patients undergoing arthroscopic shoulder joint surgery in two different types of anaesthesia – preliminary research. *Medical Studies* 2020; **36**: 18–25. https://doi.org/10.5114/ms.2020.94084.
445. Ledowski T, Wulf H. The influence of fentanyl vs. s-ketamine on intubating conditions during induction of anaesthesia with etomidate and rocuronium. *European Journal of Anaesthesiology* 2001; **18**: 519–23. https://doi.org/10.1046/j.1365-2346.2001.00886.x.
446. Lee H-Y, Kim SH, So KY, Kim DJ. Effects of interscalene brachial plexus block to intra-operative hemodynamics and postoperative pain for arthroscopic shoulder surgery. *Korean Journal of Anesthesiology* 2012; **62**: 30. https://doi.org/10.4097/kjae.2012.62.1.30.
447. Lee LHY, Irwin MG, Lui S. Intraoperative Remifentanil Infusion Does Not Increase Postoperative Opioid Consumption Compared with 70% Nitrous Oxide. *Anesthesiology* 2005; **102**: 398–402. https://doi.org/10.1097/00000542-200502000-00024.
448. Lee S, Ko JS, Kang R et al. Analgesic effects of ultrasound-guided preoperative posterior Quadratus Lumborum block in laparoscopic hepatectomy: A prospective double-blinded randomized controlled trial. *Journal of Clinical Anesthesia* 2024; **97**: 111504. https://doi.org/10.1016/j.jclinane.2024.111504.
449. Lehmann LJ, Loosen G, Weiss C, Schmittner MD. Interscalene plexus block versus general anaesthesia for shoulder surgery: a randomized controlled study. *European Journal of Orthopaedic Surgery & Traumatology* 2015; **25**: 255–61. https://doi.org/10.1007/s00590-014-1483-3.
450. Lena P, Balarac N, Arnulf JJ, Bigeon JY, Tapia M, Bonnet F. Fast-track coronary artery bypass grafting surgery under general anesthesia with remifentanil and spinal analgesia with morphine and clonidine. *Journal of Cardiothoracic and Vascular Anesthesia* 2005; **19**: 49–53. https://doi.org/10.1053/j.jvca.2004.11.009.
451. Lena P, Balarac N, Lena D et al. Fast-Track Anesthesia With Remifentanil and Spinal Analgesia for Cardiac Surgery: The Effect on Pain Control and Quality of Recovery. *Journal of Cardiothoracic and Vascular Anesthesia* 2008; **22**: 536–42. https://doi.org/10.1053/j.jvca.2008.04.012.
452. Li C, Chen Y, Han K et al. Does Intraoperative Multi-Drug Wound Infiltration Relieve Postoperative Pain Following Single-Level TLIF Surgery? A Randomized Controlled Trial. *Pain physician* 2023; **26**: 13–9.
453. Li K, Li L, Gao M et al. Application of ultrasound-guided subcostal transversus abdominis plane block in gastric cancer patients undergoing open gastrectomy. *International journal of clinical and experimental medicine* 2015; **8**: 13976–82.
454. Li M, Zhang S, Yao Y et al. Effect of General Anesthesia Combined with Anterior Serratus Muscle Plane Block on Pain Relief and Cognitive Function in Undergoing Thoracoscopic Lobectomy. *Indian Journal of Pharmaceutical Sciences* 2024; **86**. https://doi.org/10.36468/pharmaceutical-sciences.spl.844.
455. Li N, Qi X, Bao J et al. A Comparative Study of Esketamine-Propofol and Sufentanil-Propofol for Analgesia and Sedation During Breast Minimally Invasive Rotary Resection with Local Anesthesia: A Randomized Double-Blind Clinical Trial. *Drug Design, Development and Therapy* 2024; **18**: 5397–407. https://doi.org/10.2147/DDDT.S487872.
456. Li P, Ru F. Postoperative Analgesic Impacts of Ultrasound-Guided Intercostal Nerve Block with Different Concentrations of Ropivacaine in Combination with General Anesthesia for Patients Undergoing Thoracoscopic Surgery. *Journal of Biological Regulators and Homeostatic Agents* 2024; **38**. https://doi.org/10.23812/j.biol.regul.homeost.agents.20243807.450.
457. Li Q, Liao Y, Wang X, Zhan M, Xiao L, Chen Y. Efficacy of bilateral catheter superficial parasternal intercostal plane blocks using programmed intermittent bolus for opioid-sparing postoperative analgesia in cardiac surgery with sternotomy: A randomized, double-blind, placebo-controlled trial. *Journal of Clinical Anesthesia* 2024; **95**: 111430. https://doi.org/10.1016/j.jclinane.2024.111430.
458. Li Q, Zhang L, Zhou H-M, Wu X-W. Ultrasound-Guided Erector Spinae Plane Block in Elderly Patients Undergoing Total Hip Arthroplasty: A Triple-Blind, Randomized Controlled Trial. *The Journal of Arthroplasty* 2025; **40**: 999–1004. https://doi.org/10.1016/j.arth.2024.10.052.
459. Li Q, Li Q, Peng W et al. Ultrasound-guided bilateral erector spinae plane block in laparoscopic colon cancer surgery. *Die Anaesthesiologie* 2022; **71**: 224–32. https://doi.org/10.1007/s00101-021-01076-6.
460. Li X-D, Li Y-J, Fan C-Y. Efficacy and safety of ultrasound‐guided quadratus lumborum block in patients receiving percutaneous nephrolithotomy under general anaesthesia. *International Journal of Clinical Practice* 2021; **75**. https://doi.org/10.1111/ijcp.14439.
461. Li Y, Dong H, Tan S, Qian Y, Jin W. Effects of thoracic epidural anesthesia/analgesia on the stress response, pain relief, hospital stay, and treatment costs of patients with esophageal carcinoma undergoing thoracic surgery. *Medicine* 2019; **98**: e14362. https://doi.org/10.1097/MD.0000000000014362.
462. Liu J, Zheng X, Zhang Y. Comparative effects on three anesthesia methods in gynecologic laparoscopic surgery. *International Journal of Clinical and Experimental Medicine* 2017; **10**.
463. Liu L, Ni X, Zhang L, Zhao K, Xie H, Zhu J. Effects of ultrasound-guided erector spinae plane block on postoperative analgesia and plasma cytokine levels after uniportal VATS: a prospective randomized controlled trial. *Journal of Anesthesia* 2021; **35**: 3–9. https://doi.org/10.1007/s00540-020-02848-x.
464. Liu R, Qin H, Wang M, Li K, Zhao G. Transversus abdominis plane block with general anesthesia blunts the perioperative stress response in patients undergoing radical gastrectomy. *BMC anesthesiology* 2019; **19**: 205. https://doi.org/10.1186/s12871-019-0861-0.
465. Liu T, Liu J, Yang L, Wu Z, Zhang Y, Gao F. Ultrasound-guided lesser occipital nerve combined with great auricular nerve block for vestibular schwannoma craniotomy via a suboccipital retrosigmoid approach: a prospective, double-blind randomized controlled trial. *BMC Anesthesiology* 2024; **24**: 247. https://doi.org/10.1186/s12871-024-02642-2.
466. Liu XN, Noh Y-M, Yang C-J, Kim JU, Chung MH, Noh KC. Effects of a Single-Dose Interscalene Block on Pain and Stress Biomarkers in Patients Undergoing Arthroscopic Rotator Cuff Repair: A Randomized Controlled Trial. *Arthroscopy: The Journal of Arthroscopic & Related Surgery* 2017; **33**: 918–26. https://doi.org/10.1016/j.arthro.2016.09.018.
467. Liu X, Hu X, Li R, Zhang Y. Combination of post-fascia iliaca compartment block and dexmedetomidine in pain and inflammation control after total hip arthroplasty for elder patients: a randomized control study. *Journal of orthopaedic surgery and research* 2020; **15**: 42. https://doi.org/10.1186/s13018-020-1562-6.
468. Löchel J, Janz V, Leopold VJ, Krämer M, Wassilew GI. Transversus abdominis Plane Block for Improved Early Postoperative Pain Management after Periacetabular Osteotomy: A Randomized Clinical Trial. *Journal of Clinical Medicine* 2021; **10**: 394. https://doi.org/10.3390/jcm10030394.
469. Loocke JV, Heintz A, Mulier J. Impact of opioid free anesthesia on glycemia: a randomized controlled trial. *Acta Anaesthesiologica Belgica* 2022; **73**: 25–31. https://doi.org/10.56126/73.S1.27.
470. Lotfy MA, Ayaad MG. Efficacy of dexmedetomidine-based opioid-free anesthesia on the control of surgery-induced inflammatory response and outcomes in patients undergoing open abdominal hysterectomy. *Egyptian Journal of Anaesthesia* 2022; **38**: 497–504. https://doi.org/10.1080/11101849.2022.2118412.
471. Lowenstein L, Zimmer EZ, Deutsch M, Paz Y, Yaniv D, Jakobi P. Preoperative analgesia with local lidocaine infiltration for abdominal hysterectomy pain management. *European Journal of Obstetrics & Gynecology and Reproductive Biology* 2008; **136**: 239–42. https://doi.org/10.1016/j.ejogrb.2006.11.008.
472. Luchetti M, Palomba R, Sica G, Massa G, Tufano R. Effectiveness and safety of combined epidural and general anesthesia for laparoscopic cholecystectomy. *Regional Anesthesia* 1996; **21**: 465-9.
473. Luo G, Zhu J, Ni H et al. Pretreatment with Pectoral Nerve Block II Is Effective for Reducing Pain in Patients Undergoing Thoracoscopic Lobectomy: A Randomized, Double‐Blind, Placebo‐Controlled Trial. *BioMed Research International* 2021; **2021**. https://doi.org/10.1155/2021/6693221.
474. Luo Z, Zeng W, Chen X et al. Cocktail of Ropivacaine, Morphine, and Diprospan Reduces Pain and Prolongs Analgesic Effects after Total Knee Arthroplasty: A Prospective Randomized Controlled Trial. *International Journal of Clinical Practice* 2024; **2024**: 1–10. https://doi.org/10.1155/2024/3697846.
475. M N, Pandey RK, Sharma A et al. Pectoral nerve blocks to improve analgesia after breast cancer surgery: A prospective, randomized and controlled trial. *Journal of Clinical Anesthesia* 2018; **45**: 12–7. https://doi.org/10.1016/j.jclinane.2017.11.027.
476. Ma H, Perez J, Bertsch J et al. Intraoperative Non-Opiate Anesthesia for Patients Undergoing Arthroscopic Temporomandibular Joint Surgery: A Randomized Controlled Trial. *Drug Design, Development and Therapy* 2024; **18**: 4915–25. https://doi.org/10.2147/DDDT.S486134.
477. Ma J, Wang X-Y, Sun Q-X et al. Transversus abdominis plane block reduces remifentanil and propofol consumption, evaluated by closed-loop titration guided by bispectral index. *Experimental and therapeutic medicine* 2018; **16**: 3897–902. https://doi.org/10.3892/etm.2018.6707.
478. Macalou D, Trueck S, Meuret P et al. Postoperative Analgesia After Total Knee Replacement: The Effect of an Obturator Nerve Block Added to the Femoral 3-in-1 Nerve Block. *Anesthesia & Analgesia* 2004; **99**: 251–4. https://doi.org/10.1213/01.ANE.0000121350.09915.84.
479. Maguire AM, Kumar N, Parker JL, Rowbotham DJ, Thompson JP. Comparison of effects of remifentanil and alfentanil on cardiovascular response to tracheal intubation in hypertensive patients. *British Journal of Anaesthesia* 2001; **86**: 90–3. https://doi.org/10.1093/bja/86.1.90.
480. Mahmoud AM, Alsaied MA, Ragab SG, Abdelfattah YA, Farghaly OS, Shawky MA. Inter-Semispinalis Plane Block Versus General Anesthesia for Postoperative Analgesia in Posterior Cervical Spine Surgery: A Randomized Controlled Trial. *Anesthesiology and Pain Medicine* 2024; **14**. https://doi.org/10.5812/aapm-143369.
481. Makarem J, Majedi H, Bahmaee F et al. Evaluation of the Efficacy of Low-Dose Naloxone for the Prevention of Acute Remifentanil-Induced Hyperalgesia in Patients Undergoing General Anesthesia for Laparotomy. *Archives of Neuroscience* 2020; **7**. https://doi.org/10.5812/ans.108836.
482. Makkar JK, Dahiya D, Jain D, Kuber A, Kajal K, Singh NP. Effect of balanced anaesthesia with and without modified pectoralis nerve block on postoperative analgesia after breast surgeries: A randomised controlled trial. *Indian Journal of Anaesthesia* 2021; **65**: S110–4. https://doi.org/10.4103/ija.IJA_125_21.
483. Manrique F, Pardo GC, Forero FC, Leon CT, Armesto DR, Chaverra S. Estudio controlado aleatorio, efectividad analgésica del bloqueo del plano erector espinal en cirugía hepatopancreatobiliar. *Archivos de Medicina (Manizales)* 2024; **24**. https://doi.org/10.30554/archmed.24.2.5087.2024.
484. Manzoor S, Khan T, Zahoor S et al. Post-thoracotomy ipsilateral shoulder pain: What should be preferred to optimize it - phrenic nerve infiltration or paracetamol infusion? *Annals of Cardiac Anaesthesia* 2019; **22**: 291. https://doi.org/10.4103/aca.ACA_76_18.
485. Mariano ER, Watson D, Loland VJ et al. Bilateral infraorbital nerve blocks decrease postoperative pain but do not reduce time to discharge following outpatient nasal surgery. *Canadian Journal of Anesthesia/Journal canadien d’anesthésie* 2009; **56**: 584–9. https://doi.org/10.1007/s12630-009-9119-5.
486. Marochkov A, Yaskevich V. Multi-injection paravertebral block with low volume local anesthetic for anesthesia in radical mastectomy – randomized controlled trial. *Anaesthesiology Intensive Therapy* 2021; **53**: 141–5. https://doi.org/10.5114/ait.2021.105649.
487. Maru ND, Sharma A, Pravinbhai VN. ULTRASOUND GUIDED BILATERAL RECTUS SHEATH BLOCK IN MANAGEMENT OF POSTOPERATIVE PAIN IN LAPAROSCOPIC GYNECOLOGIC SURGERY: A RANDOMIZED DOUBLE BLIND CONTROLLED STUDY. *International Journal of Medicine and Public Health*, 2024; **14**: 20-4. https://doi.org/10.70034/ijmedph.2024.4.5
488. Massoth C, Schwellenbach J, Saadat-Gilani K et al. Impact of opioid-free anaesthesia on postoperative nausea, vomiting and pain after gynaecological laparoscopy - A randomised controlled trial. *Journal of Clinical Anesthesia* 2021; **75**: 110437. https://doi.org/10.1016/j.jclinane.2021.110437.
489. Matsumoto M, Flores EM, Kimachi PP et al. Benefits in radical mastectomy protocol: a randomized trial evaluating the use of regional anesthesia. *Scientific Reports* 2018; **8**: 7815. https://doi.org/10.1038/s41598-018-26273-z.
490. Mazloomdoost D, Pauls RN, Hennen EN et al. Liposomal bupivacaine decreases pain following retropubic sling placement: a randomized placebo-controlled trial. *American Journal of Obstetrics and Gynecology* 2017; **217**: 598.e1-598.e11. https://doi.org/10.1016/j.ajog.2017.07.001.
491. Mazzinari G, Rovira L, Casasempere A et al. Interfascial block at the serratus muscle plane versus conventional analgesia in breast surgery: a randomized controlled trial. *Regional Anesthesia & Pain Medicine* 2019; **44**: 52–8. https://doi.org/10.1136/rapm-2018-000004.
492. Mehta Y, Vats M, Sharma M, Arora R, Trehan N. Thoracic epidural analgesia for off-pump coronary artery bypass surgery in patients with chronic obstructive pulmonary disease. *Annals of Cardiac Anaesthesia* 2010; **13**: 224. https://doi.org/10.4103/0971-9784.69062.
493. Memedov C, Mentes O, Simsek A et al. Comparison Of Analgesic Effects Of Intraperitoneal Lornoxicam And Ropivacaine Administration In Laparoscopic Cholecystectomy. *Medical Journal of Trakya University* 2008; **27**: 142-49 https://doi.org/10.5174/tutfd.2008.01046.2.
494. Menck JT, Tenório SB, Oliveira RM de et al. Opioid-free Anesthesia for Laparoscopic Gastroplasty. A Prospective and Randomized Trial. *The Open Anesthesia Journal* 2022; **16**. https://doi.org/10.2174/25896458-v16-e2208110.
495. Menshawi MA, Fahim HM. Dexmedetomidine versus remifentanil infusion for controlled hypotension in shoulder arthroscopy: a comparative study. *Ain-Shams Journal of Anesthesiology* 2020; **12**: 21. https://doi.org/10.1186/s42077-020-00072-z.
496. Messner M, Albrecht S, Lang W, Sittl R, Dinkel M. The Superficial Cervical Plexus Block for Postoperative Pain Therapy in Carotid Artery Surgery. A Prospective Randomised Controlled Trial. *European Journal of Vascular and Endovascular Surgery* 2007; **33**: 50–4. https://doi.org/10.1016/j.ejvs.2006.06.024.
497. Miguel R, Barlow I, Morrell M, Scharf J, Sanusi D, Fu E. A Prospective, Randomized, Double-blind Comparison of Epidural and Intravenous Sufentanil Infusions. *Anesthesiology* 1994; **81**: 346–52. https://doi.org/10.1097/00000542-199408000-00012.
498. Minqiang L, Mingfei M, Fengzhu H et al. Assessing the clinical advantage of opioid-reduced anesthesia in thoracoscopic sympathectomy: a prospective randomized controlled trial. *BMC Anesthesiology* 2024; **24**: 325. https://doi.org/10.1186/s12871-024-02711-6.
499. Misk M, Alshawadfy A, Lamei M, Khames F, Elgawad MA, Hendawy HA. Evaluating the perioperative analgesic effect of ultrasound-guided trigeminal nerve block in adult patients undergoing maxillofacial surgery under general anesthesia: A randomized controlled study. *Egyptian Journal of Anaesthesia* 2023; **39**: 598–604. https://doi.org/10.1080/11101849.2023.2238529.
500. Mitsunaga JK, Calsavara VF, Onari ES et al. Spinal block and delirium in oncologic patients after laparoscopic surgery in the Trendelenburg position: A randomized controlled trial. *PLOS ONE* 2021; **16**: e0249808. https://doi.org/10.1371/journal.pone.0249808.
501. Mittal T, Dey A, Siddhartha R, Nali A, Sharma B, Malik V. Efficacy of ultrasound-guided transversus abdominis plane (TAP) block for postoperative analgesia in laparoscopic gastric sleeve resection: a randomized single blinded case control study. *Surgical Endoscopy* 2018; **32**: 4985–9. https://doi.org/10.1007/s00464-018-6261-6.
502. Miu M, Royer C, Gaillat C et al. Lack of Analgesic Effect Induced by Ropivacaine Wound Infiltration in Thyroid Surgery. *Anesthesia & Analgesia* 2016; **122**: 559–64. https://doi.org/10.1213/ANE.0000000000001041.
503. Mohamed RM, Morsy I, Hamed JMD. Bilateral ultrasound guided erector spinae plane block vs. preoperative pregabalin for postoperative pain management in lumbar spine surgery. *Anaesthesia, Pain & Intensive Care* 2023; **27**: 513-20. https://doi.org/10.35975/apic.v27i5.2306
504. Mohammed HA, Belal AM, Elkalla RS, Soliman SM. Analgesic efficacy of pre-emptive ultrasound-guided mid-point transverse process to pleura block for patients undergoing posterolateral thoracotomy incisions: Randomized controlled trial. *Egyptian Journal of Anaesthesia* 2023; **39**: 538–45. https://doi.org/10.1080/11101849.2023.2231709.
505. Mohasseb AM, Elebiedy MG, Mohammed MN. A randomised comparative study of erector spinae plane block versus low-dose ketamine-dexmedetomidine intravenous infusion as intraoperative opioid-free analgesia for modified radical mastectomy. *Indian Journal of Anaesthesia* 2024; **68**: 651–7. https://doi.org/10.4103/ija.ija_1167_23.
506. Molins G, Valls-Ontañón A, Nadal MD, Hernández-Alfaro F. Ultrasound-Guided Suprazygomatic Maxillary Nerve Block Is Effective in Reducing Postoperative Opioid Use Following Bimaxillary Osteotomy. *Journal of Oral and Maxillofacial Surgery* 2024; **82**: 412–21. https://doi.org/10.1016/j.joms.2023.12.018.
507. Moller JF, Nikolajsen L, Rodt SA, Ronning H, Carlsson PS. Thoracic Paravertebral Block for Breast Cancer Surgery: A Randomized Double-Blind Study. *Anesthesia & Analgesia* 2007; **105**: 1848–51. https://doi.org/10.1213/01.ane.0000286135.21333.fd.
508. Mondal S, Pandey RK, Kumar M, Sharma A, Darlong V, Punj J. Analgesic efficacy of classical thoracolumbar interfascial plane block versus modified thoracolumbar interfascial plane block in patients undergoing lumbar disc surgeries: A comparative, randomised controlled trial. *Indian Journal of Anaesthesia* 2024; **68**: 366–73. https://doi.org/10.4103/ija.ija_1153_23.
509. Mont LS du, Jazayeri A, Besch G, Guinot P-G, Steinmetz E. Continuous Transversus Abdominis Plane Infusion via Surgically Inserted Catheter for Postoperative Analgesia After Abdominal Aortic Surgery by Retroperitoneal Approach: TAPCACAO Study. *Annals of Vascular Surgery* 2022; **86**: 104–10. https://doi.org/10.1016/j.avsg.2022.05.034.
510. Morel J, Pascal J, Charier D et al. Preoperative Peribulbar Block in Patients Undergoing Retinal Detachment Surgery Under General Anesthesia: A Randomized Double-Blind Study. *Anesthesia & Analgesia* 2006; **102**: 1082–7. https://doi.org/10.1213/01.ane.0000198638.93784.42.
511. Moro FD, Aiello L, Pavarin P, Zattoni F. Ultrasound-guided transversus abdominis plane block (US-TAPb) for robot-assisted radical prostatectomy: a novel ‘4-point’ technique—results of a prospective, randomized study. *Journal of Robotic Surgery* 2019; **13**: 147–51. https://doi.org/10.1007/s11701-018-0858-6.
512. Morrow BC, Milligan KR, Murthy BVS. Analgesia following day‐case knee arthroscopy—the effect of piroxicam with or without bupivacaine infiltration. *Anaesthesia* 1995; **50**: 461–3. https://doi.org/10.1111/j.1365-2044.1995.tb06006.x.
513. Moslemiid F, Gadamkheyr E, Gaderi M. Evaluation of postoperative shivering with remifentanil-propofol intravenous anesthesia in ambulatory gynecologic procedures: the relationship with intraoperative core body temperature. *Crescent Journal of Medical and Biological Sciences* 2020*.*
514. Mostafa H, Shamaa HE, Refaai NE, Akati AE. Randomized Double Blind Comparison Between Sciatic-Femoral Nerve Block and Propofol-Remifentanil, Propofol-Alfentanil General Anesthetics in Out-Patient Knee Arthroscopy. *Pakistan Journal of Biological Sciences* 2008; **11**: 359–65. https://doi.org/10.3923/pjbs.2008.359.365.
515. Mostafa RH, Gohneim MA-F, El-din DMK, Ismaiel M, Abdelmohsen I, Refaat S. Effect of Scalp Block on Postoperative nausea and vomiting & Recovery Profile after Craniotomy: A Randomized, Double-Blind, Controlled Study: Postoperative nausea and vomiting after Craniotomy. *Journal of Cellular & Molecular Anesthesia* 2020; **5**: 235-45. https://doi.org/10.22037/jcma.v5i4.32015.
516. Mostafa SF, Elyazed MMA, Eid GM, Belal AM. Inter-semispinal plane (ISP) block for postoperative analgesia following cervical spine surgery: A prospective randomized controlled trial. *Journal of Clinical Anesthesia* 2022; **83**: 110974. https://doi.org/10.1016/j.jclinane.2022.110974.
517. Moussa AA. Opioid saving strategy: bilateral single-site thoracic paravertebral block in right lobe donor hepatectomy. *Middle East journal of anaesthesiology* 2008; **19**: 789–801.
518. Nair A, Amula VE, Naik V et al. Comparison of Postoperative Analgesia in Patients Undergoing Ileostomy Closure with and Without Dual Transversus Abdominis Plane (TAP) Block: A Randomized Controlled Trial. *Rambam Maimonides Medical Journal* 2019; **10**: e0004. https://doi.org/10.5041/RMMJ.10356.
519. Naja MZ, El-Rajab M, Kabalan W et al. Effectiveness of local anaesthesia (clonidine and fentanyl) infiltration for post-submucosal resection pain relief: a randomized, double-blinded clinical trial. *The Journal of Laryngology & Otology* 2007; **121**: 19–24. https://doi.org/10.1017/S0022215106003306.
520. Naja MZ, Ziade MF, Lönnqvist PA. General anaesthesia combined with bilateral paravertebral blockade (T5–6) vs. general anaesthesia for laparoscopic cholecystectomy: a prospective, randomized clinical trial. *European Journal of Anaesthesiology* 2004; **21**: 489–95. https://doi.org/10.1017/S026502150400612X.
521. Naja Z, Tannir MA, Naccache N et al. General anaesthesia combined with bilateral paravertebral blockade (T11-T12-L1) vs general anaesthesia for bilateral varicocelectomy. A randomized double-blind clinical trial. *Le Journal medical libanais. The Lebanese medical journal* 2011; **59**: 126–30.
522. Najeeb H, Mehdi S, Siddiqui A, Batool S. Pectoral Nerves I, II and Serratus Plane Blocks in Multimodal Analgesia for Mastectomy: A Randomised Clinical Trial. *Journal of the College of Physicians and Surgeons Pakistan* 2019; **29**: 910–4. https://doi.org/10.29271/jcpsp.2019.10.910.
523. Nalankilli VP, Christopher PJ, Motati H et al. A randomized controlled trial on the effectiveness of laparoscopic-guided transversus abdominis plane block in patients undergoing laparoscopic IPOM plus. *Hernia* 2024; **28**: 1257–63. https://doi.org/10.1007/s10029-024-03016-w.
524. Nam SW, Do S-H, Hwang J-W, Park I, Hwang I, Na H-S. Effects of opioid-sparing general anesthesia on postoperative nausea and vomiting in laparoscopic gynecological surgery. *Korean journal of anesthesiology* 2024; **77**: 605–13. https://doi.org/10.4097/kja.24336.
525. Nashibi M. Evaluation of ultrasound guided erector spinae plane block efficacy on post operative pain in lumbar spine surgery: a randomized clinical trial. *Ağrı - The Journal of The Turkish Society of Algology* 2022; **34**: 174-79. https://doi.org/10.14744/agri.2021.04864.
526. Negi S, Sen I, Arya V, Sharma A. Dexmedetomidine versus fentanyl as coadjuvants of balanced anaesthesia technique in renal transplant recipients. *Middle East journal of anaesthesiology* 2014; **22**: 549–57.
527. Nehra D, Gemmell L, Pye JK. Pain relief after inguinal hernia repair: A randomized double-blind study. *Journal of British Surgery* 1995; **82**: 1245–7. https://doi.org/10.1002/bjs.1800820929.
528. Neil L. Effect of Dexmedetomidine Versus Fentanyl on Haemodynamic Response to Patients Undergoing Elective Laparoscopic Surgery: A Double Blinded Randomized Controlled Study. *Journal of Clinical and Diagnostic Research* 2017; **11**: 1-4. https://doi.org/10.7860/JCDR/2017/24152.9563.
529. Neuburger PJ, Ngai JY, Chacon MM et al. A Prospective Randomized Study of Paravertebral Blockade in Patients Undergoing Robotic Mitral Valve Repair. *Journal of Cardiothoracic and Vascular Anesthesia* 2015; **29**: 930–6. https://doi.org/10.1053/j.jvca.2014.10.010.
530. Neupane A, Jain D, Arora S et al. Evaluation of ultrasound-guided suprazygomatic maxillary nerve block in functional endoscopic sinus surgery for postoperative pain relief: A randomised controlled trial. *Indian Journal of Anaesthesia* 2024; **68**: 706–11. https://doi.org/10.4103/ija.ija_81_24.
531. Nho J-H, Jang B-W, An CY et al. General versus Brachial Plexus Block Anesthesia in Pain Management after Internal Fixation in Patients with Distal Radius Fracture: A Randomized Controlled Trial. *International Journal of Environmental Research and Public Health* 2022; **19**: 9155. https://doi.org/10.3390/ijerph19159155.
532. Niraj G, Searle A, Mathews M et al. Analgesic efficacy of ultrasound-guided transversus abdominis plane block in patients undergoing open appendicectomy. *British Journal of Anaesthesia* 2009; **103**: 601–5. https://doi.org/10.1093/bja/aep175.
533. Noorain Z, Waqar SH, Shah SA, Huddah S, Shah SA. COMPARISON OF POSTOPERATIVE PAIN WITH AND WITHOUT PECTORAL BLOCK IN PATIENTS UNDERGOING MODIFIED RADICAL MASTECTOMY. *Journal of Ayub Medical College Abbottabad* 2024; **36**: 25–8. https://doi.org/10.55519/JAMC-01-12588.
534. Norris EJ, Beattie C, Perler BA et al. Double-masked Randomized Trial Comparing Alternate Combinations of Intraoperative Anesthesia and Postoperative Analgesia in Abdominal Aortic Surgery. *Anesthesiology* 2001; **95**: 1054–67. https://doi.org/10.1097/00000542-200111000-00006.
535. Obersztyn M, Trejnowska E, Nadziakiewicz P, Knapik P. Evaluation of thoracic epidural analgesia in patients undergoing coronary artery bypass surgery – a prospective randomized trial. *Polish Journal of Cardio-Thoracic Surgery* 2018; **15**: 72–8. https://doi.org/10.5114/kitp.2018.76471.
536. Oh TK, Yim J, Kim J et al. Effects of preoperative ultrasound-guided transversus abdominis plane block on pain after laparoscopic surgery for colorectal cancer: a double-blind randomized controlled trial. *Surgical Endoscopy* 2017; **31**: 127–34. https://doi.org/10.1007/s00464-016-4941-7.
537. Okello MO, Mung’ayi V, Adam R, Kabugi J. A comparison of risk of hypotension using standard doses of remifentanil versus dexmedetomidine infusions in adult patients undergoing surgery under general anaesthesia at the Aga Khan University Hospital, Nairobi. *African Health Sciences* 2018; **18**: 1267. https://doi.org/10.4314/ahs.v18i4.48.
538. Ökmen K, Ökmen BM. Ultrasound-guided anterior quadratus lumborum block for postoperative pain after percutaneous nephrolithotomy: a randomized controlled trial. *Korean Journal of Anesthesiology* 2020; **73**: 44–50. https://doi.org/10.4097/kja.19175.
539. Ökmen K, Ökmen BM, Topal S. Ultrasound-guided posterior quadratus lumborum block for postoperative pain after laparoscopic cholecystectomy: A randomized controlled double blind study. *Journal of Clinical Anesthesia* 2018; **49**: 112–7. https://doi.org/10.1016/j.jclinane.2018.06.027.
540. Omar AM. Postoperative continuous transversus abdominis plane block vs continuous wound infusion of levobupivacaine in females undergoing open gynecologic procedures. *Egyptian Journal of Anaesthesia* 2013; **29**: 383–8. https://doi.org/10.1016/j.egja.2013.05.011.
541. Ömür D, Oğuzalp H, Kiraz HA et al. The analgesic efficacy of ultrasound-guided transversus abdominis plane block on postoperative pain and morphine consumption in varicocelectomy. *Saudi medical journal* 2016; **37**: 648–55. https://doi.org/10.15537/Smj.2016.6.14332.
542. Onan B, Onan IS, Kilickan L, Sanisoglu I. Effects of Epidural Anesthesia on Acute and Chronic Pain After Coronary Artery Bypass Grafting. *Journal of Cardiac Surgery* 2013; **28**: 248–53. https://doi.org/10.1111/jocs.12086.
543. Ong JC, Lin CP, Fook-Chong SM, Tang A, Ying YK, Keng TB. Continuous Infiltration of Local Anaesthetic following Total Knee Arthroplasty. *Journal of Orthopaedic Surgery* 2010; **18**: 203–7. https://doi.org/10.1177/230949901001800214.
544. Ongewe A, Mung’ayi V, Bal R. Effect of low-dose ketamine versus fentanyl on attenuating the haemodynamic response to laryngoscopy and endotracheal intubation in patients undergoing general anaesthesia: a prospective, double-blinded, randomised controlled trial. *African health sciences* 2019; **19**: 2752–63. https://doi.org/10.4314/ahs.v19i3.51.
545. Onishi E, Murakami M, Nishino R, Ohba R, Yamauchi M. Analgesic Effect of Double-Level Retrolaminar Paravertebral Block for Breast Cancer Surgery in the Early Postoperative Period: A Placebo-Controlled, Randomized Clinical Trial. *The Tohoku Journal of Experimental Medicine* 2018; **245**: 179–85. https://doi.org/10.1620/tjem.245.179.
546. Ornek D, Metin S, Deren S et al. The influence of various anesthesia techniques on postoperative recovery and discharge criteria among geriatric patients. *Clinics* 2010; **65**: 941–6. https://doi.org/10.1590/S1807-59322010001000003.
547. Orsolya M, Zoltan MA, Gherman V et al. The effect of anaesthetic management on neutrophil gelatinase associated lipocalin (NGAL) levels after robotic surgical oncology. *Journal of BUON*, 2015: **20**; 317-24.
548. Ouaz M, Attia H, Hmida NB, Driss N, Majdoub A. Bloc cervical intermédiaire écho-guidé lors de la thyroïdectomie totale sous anesthésie générale : effet sur l’épargne morphinique per- et postoperatoire, étude contrôlée randomisée. *Anesthésie & Réanimation* 2019; **5**: 10–5. https://doi.org/10.1016/j.anrea.2018.03.015.
549. Owczuk R, Dylczyk-Sommer A, Wojciechowski J et al. The influence of epidural blockade on gut permeability in patients undergoing open surgical repair of abdominal aortic aneurysm. *Anestezjologia Intensywna Terapia* 2016; **48**: 122–7. https://doi.org/10.5603/AIT.a2016.0014.
550. Oza V, Vadgama P, Parmar V, Rawal K. A STUDY OF EFFICACY OF IITV-GUIDED ERECTOR SPINAE PLANE (ESP) BLOCK AS NOVEL MULTIMODAL ANALGESIA TECHNIQUE FOR POSTOPERATIVE ANALGESIA IN MODIFIED RADICAL MASTECTOMY(MRM). *International Journal of Academic Medicine and Pharmacy* 2024; **6**: 991–5.
551. Ozcan A, Ozcan N, Gulec H, Yalcin F, Basar H. Comparison of the effects of fentanyl, remifentanil, and dexmedetomidine on neuromuscular blockade. *Journal of Anesthesia* 2012; **26**: 196–9. https://doi.org/10.1007/s00540-011-1270-9.
552. Ozcan S, Ozer AB, Yasar MA, Erhan OL. Effects of combined general anesthesia and thoracic epidural analgesia on cytokine response in patients undergoing laparoscopic cholecystectomy. *Nigerian Journal of Clinical Practice* 2016; **19**: 436. https://doi.org/10.4103/1119-3077.183308.
553. Özdilek A, Beyoğlu ÇA, Demirdağ Ç et al. Perioperative Analgesic Effects of Preemptive Ultrasound-Guided Subcostal Transversus Abdominis Plane Block for Percutaneous Nephrolithotomy: A Prospective, Randomized Trial. *Journal of Endourology* 2020; **34**: 434–40. https://doi.org/10.1089/end.2019.0766.
554. Özgün H, Kurt MN, Kurt I, Çevikel MH. Comparison of Local, Spinal, and General Anaesthesia for Inguinal Herniorrhaphy. *The European Journal of Surgery* 2002; **168**: 455–9. https://doi.org/10.1080/110241502321116442.
555. Ozgun M, Hosten T, Solak M. Effect of Bilateral Superficial Cervical Plexus Block on Postoperative Analgesic Consumption in Patients Undergoing Thyroid Surgery. *Cureus* 2022; **14**: e21212. https://doi.org/10.7759/cureus.21212.
556. Özhan MÖ, Tanrıöver A, Atik B, Çaparlar CÖ, Eşkin BM, Süzer AM. Preoperative ankle block for postoperative analgesia in foot surgery. *Der Anaesthesist* 2020; **69**: 565–72. https://doi.org/10.1007/s00101-020-00754-1.
557. Oztekin U, Caniklioglu M, Selmi V et al. Do Anesthesia Methods in Retrograde Intrarenal Surgery Make Difference Regarding the Success of Ureteral Access and Surgical Outcomes? *Journal of Laparoendoscopic & Advanced Surgical Techniques* 2020; **30**: 273–7. https://doi.org/10.1089/lap.2019.0548.
558. Padun J, Basumatary B, Das A, Sonowal D. A Comparative Study of Intrathecal Dexmedetomidine and Fentanyl as Adjuvants to Bupivacaine in Lower Abdominal, Perineal and Lower Limb Surgeries. *International Journal of Toxicological and Pharmacological Research* 2023; **13**: 128–40.
559. Paikra PK, Mahobia M, Kashyap A, Singh M, Jain A. Epidural Injection of Local Anesthetic As an Adjunct to General Anesthesia Prolongs Postoperative Analgesia in Elective Laminectomy/Discectomy Cases. *International Journal of Pharmaceutical and Clinical Research* 2023; **15**: 160–5.
560. Pal R, Rajput S, Arora KK. Opioid-based Anaesthesia versus Opioid Free Anaesthesia in Laparoscopic Cholecystectomies: A Randomised Clinical Study. *Journal of Clinical and Diagnostic Research* 2023; **17**: 10-14. https://doi.org/10.7860/JCDR/2023/64362.18152.
561. Palanne R, Rantasalo M, Vakkuri A et al. Effects of anaesthesia method and tourniquet use on recovery following total knee arthroplasty: a randomised controlled study. *British Journal of Anaesthesia* 2020; **125**: 762–72. https://doi.org/10.1016/j.bja.2020.03.036.
562. Paliwal N, Maurya N, Suthar OP, Janweja S. Segmental thoracic spinal anesthesia versus general anesthesia for breast cancer surgery: A prospective randomized-controlled open-label trial. *Journal of Anaesthesiology Clinical Pharmacology* 2022; **38**: 560–5. https://doi.org/10.4103/joacp.JOACP_679_20.
563. Pan PH, James CF. Anesthetic-postoperative morphine regimens for cesarean section and postoperative oxygen saturation monitored by a telemetric pulse oximetry network for 24 continuous hours. *Journal of Clinical Anesthesia* 1994; **6**: 124–8. https://doi.org/10.1016/0952-8180(94)90009-4.
564. Pandazi AK, Louizos AA, Stivaktakis JM, Davilis DJ, Georgiou LG. Inhalational Anesthetic Technique in Microlaryngeal Surgery: A Comparison between Sevoflurane-Remifentanil and Sevoflurane-Alfentanil Anesthesia. *Annals of Otology, Rhinology & Laryngology* 2003; **112**: 373–8. https://doi.org/10.1177/000348940311200414.
565. Pandey P, Singh M, Varandani S, Singh R. The Effectiveness of Bilateral Superficial Cervical Plexus Block as a Preemptive Analgesia in Thyroid Surgery Performed Under General Anaesthesia. *Research Journal of Medical Science* 2023; 757-762. https://doi.org/10.36478/10.59218/ makrjms.2023.757.762
566. Pandey SP, Yadav U, Khan MMA, Singh AK, Verma S, Nigam S. Efficacy of Ultrasound-Guided Erector Spinae Plane Block in Percutaneous Nephrolithotomy. *Cureus*2023; **15**: e40186. https://doi.org/10.7759/cureus.40186.
567. Papadima A, Boutsikou M, Lagoudianakis EE et al. Lymphocyte apoptosis after major abdominal surgery is not influenced by anesthetic technique: a comparative study of general anesthesia versus combined general and epidural analgesia. *Journal of Clinical Anesthesia* 2009; **21**: 414–21. https://doi.org/10.1016/j.jclinane.2008.10.015.
568. Parikh D, Patkar G, Ganvir M, Sawant A, Tendolkar B. Is segmental epidural anaesthesia an optimal technique for patients undergoing percutaneous nephrolithotomy? *Indian Journal of Anaesthesia* 2017; **61**: 308. https://doi.org/10.4103/0019-5049.204247.
569. Parikh G, Shah V, Vora K, Parikh B, Modi M, Panchal A. Ultrasound guided peritubal infiltration of 0.25% ropivacaine for postoperative pain relief in percutaneous nephrolithotomy. *Middle East Journal Anestheisilogy* 2013; **22**: 149-54.
570. Park J-S, Kim K-J, Lee JH, Jeong W-Y, Lee J-R. A Randomized Comparison of Remifentanil Target-Controlled Infusion Versus Dexmedetomidine Single-Dose Administration. *American Journal of Therapeutics* 2016; **23**: e690–6. https://doi.org/10.1097/01.mjt.0000433939.84373.2d.
571. Park K-B, Cho H-O, Kim MS, Jeon YD. Rebound Pain After Arthroscopic Cuff Repair With Interscalene Brachial Plexus Block Anesthesia Is Reduced by Surgeon-Administered Multimodal Shoulder Injections: A Prospective Randomized Controlled Trial. *Arthroscopy: The Journal of Arthroscopic & Related Surgery* 2025; **41**: 1291–8. https://doi.org/10.1016/j.arthro.2024.07.029.
572. Parthasarathy S, Krishnapriyanka KJ, Saravanan B. Effectiveness of pre-emptive nerve block on opioid consumption in patients undergoing nasal surgery under general anaesthesia: A double-blinded randomised controlled study. *Indian Journal of Anaesthesia* 2022; **66**: 133–9. https://doi.org/10.4103/ija.ija_813_21.
573. Patel H, Shah N, Syed A, Shah P, Macwan S. Evaluating the Analgesic Efficacy of Superficial Cervical Plexus Block for Head and Neck Surgeries: A Comparative Randomized Control Study. *Cureus* 2023; **15**: e39303 https://doi.org/10.7759/cureus.39303.
574. Patel MA, Gadsden JC, Nedeljkovic SS et al. Brachial Plexus Block with Liposomal Bupivacaine for Shoulder Surgery Improves Analgesia and Reduces Opioid Consumption: Results from a Multicenter, Randomized, Double-Blind, Controlled Trial. *Pain Medicine* 2020; **21**: 387-400. https://doi.org/10.1093/pm/pnz103.
575. Peddapally A, Vaithiyalingam E, Ettiyan S, Veeraraghavan H. Combination of Dexmedetomidine with Bupivacaine versus Fentanyl with Bupivacaine Intrathecally for Prolongation of Postoperative Analgesia in Lower Limb Surgeries: A Randomised Clinical Study. *Journal of Clinical and Diagnostic Research* 2024; **18**: 30-34. https://doi.org/10.7860/JCDR/2024/63889.19075.
576. Pei L, Zhou Y, Tan G et al. Ultrasound-Assisted Thoracic Paravertebral Block Reduces Intraoperative Opioid Requirement and Improves Analgesia after Breast Cancer Surgery: A Randomized, Controlled, Single-Center Trial. *PLOS ONE* 2015; **10**: e0142249. https://doi.org/10.1371/journal.pone.0142249.
577. Peksoz U, Celik M, Alici HA, Celik SM, Yayik AM, Ahiskalioglu A. The Effect of Transmuscular Quadratus Lumborum Block on Postoperative Opioid Consumption in Patients Undergoing Percutaneous Nephrolithotomy: A Randomized Controlled Study. *Cureus* 2021; **13**: e18344. https://doi.org/10.7759/cureus.18344.
578. Pellicano M, Zullo F, Fiorentino A, Tommaselli G. Conscious sedation versus general anaesthesia for minilaparoscopic gamete intra-Fallopian transfer: a prospective randomized study. *Human Reproduction* 2001; **16**: 2295–7. https://doi.org/10.1093/humrep/16.11.2295.
579. Peng P, Claxton A, Chung F, Chan V, Miniaci A, Krishnathas A. Femoral nerve block and ketorolac in patients undergoing anterior cruciate ligament reconstruction. *Canadian Journal of Anesthesia/Journal canadien d’anesthésie* 1999; **46**: 919–24. https://doi.org/10.1007/BF03013124.
580. Perniola A, Magnuson A, Axelsson K, Gupta A. Intraperitoneal Local Anesthetics Have Predominant Local Analgesic Effect. *Anesthesiology* 2014; **121**: 352–61. https://doi.org/10.1097/ALN.0000000000000267.
581. Pham MQ, Nguyen AX, Tran TTP. Bilateral superficial cervical plexus block improves pain control after thyroidectomy under general anesthesia: a randomized, double-blind, clinical trial. *Anaesthesia, Pain & Intensive Care* 2023; **27**: 214–9. https://doi.org/10.35975/apic.v27i2.2185.
582. Pi J, Sun Y, Zhang Z, Wan C. Combined anesthesia shows better curative effect and less perioperative neuroendocrine disorder than general anesthesia in early stage NSCLC patients. *The Journal of international medical research* 2019; **47**: 4743–52. https://doi.org/10.1177/0300060519862102.
583. Pikramenos K, Zachou M, Apostolatou E et al. The effects of method of anaesthesia on the safety and effectiveness of Radical Retropubic Prostatectomy. *Archivio Italiano di Urologia e Andrologia* 2022; **94**: 396–400. https://doi.org/10.4081/aiua.2022.4.396.
584. Ping-Chen, Lin Q-S, Lin X-Z. Optimal concentration of the transversus abdominis plane block in enhanced recovery after surgery protocols for patients of advanced age undergoing laparoscopic rectal cancer surgery. *Journal of International Medical Research* 2018; **46**: 4437–46. https://doi.org/10.1177/0300060518790699.
585. Pipaliya K, Ramanuj R. Evaluating the Efficacy of Intraperitoneal Bupivacaine Alone, or with Dexmedetomidine or with Tramadol for Pain Management Following Total Laparoscopic Hysterectomy. *International Journal of Pharmaceutical and Clinical Research* 2023; **15**: 269–77.
586. Pirie K, Doane MA, Riedel B, Myles PS. Analgesia for major laparoscopic abdominal surgery: a randomised feasibility trial using intrathecal morphine. *Anaesthesia* 2022; **77**: 428–37. https://doi.org/10.1111/anae.15651.
587. Plantevin F, Pascal J, Morel J et al. Effect of mandibular nerve block on postoperative analgesia in patients undergoing oropharyngeal carcinoma surgery under general anaesthesia. *British Journal of Anaesthesia* 2007; **99**: 708–12. https://doi.org/10.1093/bja/aem242.
588. Poonkuzhali G, Elakkiya E, Nalina M, Radha S. A study on the efficacy of erector spinae plane block with ropivacaine as post-op analgesia for modified radical mastectomy surgery. *International Journal of Academic Medicine and Pharmacy* 2023; **5**: 1488–92. https://doi.org/10.47009/jamp.2023.5.3.303.
589. Pourseidi B, Khorram-Manesh A. Effect of intercostals neural blockade with Marcaine (bupivacaine) on postoperative pain after laparoscopic cholecystectomy. *Surgical Endoscopy* 2007; **21**: 1557–9. https://doi.org/10.1007/s00464-006-9181-9.
590. Prabhu M, Clapp MA, McQuaid-Hanson E et al. Liposomal Bupivacaine Block at the Time of Cesarean Delivery to Decrease Postoperative Pain. *Obstetrics & Gynecology* 2018; **132**: 70–8. https://doi.org/10.1097/AOG.0000000000002649.
591. Pradeep, Sameera, Ldr S. Assessment of the safety and efficacy of laparoscopic cholecystectomy under spinal anaesthesia vs general anaesthesia: a comparative study. *International Journal of Life Sciences, Biotechnology and Pharma* 2023; **12**.
592. Pradhan A, Mohapatra A, Rout B, Mishra HS. Feasibility and Safety of Laparoscopic Appendicectomy under Spinal anaesthesia Vs General Anaesthesia: a Comparative Study in a Tertiary Health Care. *International Journal of Pharmaceutical and Clinical Research* 2024; **16**.
593. Prasad MK, Jain P, Varshney RK, Khare A, Jheetay GS. Tumescent local anesthesia as an alternative to general anesthesia in release of post-burn neck contracture and skin graft harvesting: A comparative study. *Anaesthesia, Pain & Intensive Care* 2021; **25**. https://doi.org/10.35975/apic.v25i1.1434.
594. Punithavathy D, Farissa A, Haniharan K, Mushahida S. Comparative study of efficacy of intrathecal fentanyl. Dexmedetomidine and fentanyl-dexmedetomidine combination as adjuvants to 0.5% hyperbaric bupivacaine for lower limb orthopaedic surgery. *International Journal of Academic Medicine and Pharmacy* 2024; **6**: 1232-7.
595. Purwar B, Ismail KM, Turner N et al. General or Spinal Anaesthetic for Vaginal Surgery in Pelvic Floor Disorders (GOSSIP): a feasibility randomised controlled trial. *International Urogynecology Journal* 2015; **26**: 1171–8. https://doi.org/10.1007/s00192-015-2670-4.
596. Pusch F, Freitag H, Weinstabl C, Obwegeser R, Huber E, Wildling E. Single‐injection paravertebral block compared to general anaesthesia in breast surgery. *Acta Anaesthesiologica Scandinavica* 1999; **43**: 770–4. https://doi.org/10.1034/j.1399-6576.1999.430714.x.
597. Qiu L, Bu X, Shen J et al. Observation of the analgesic effect of superficial or deep anterior serratus plane block on patients undergoing thoracoscopic lobectomy. *Medicine* 2021; **100**: e24352. https://doi.org/10.1097/MD.0000000000024352.
598. Qiu Y, Li X. The effect of general anesthesia compound thoracic paravertebral blockade on postoperative pain relief for patients in thoracic surgery. *Biomedical Research* 2017; **28**: 1638-41.
599. Qiu Y, Lu X, Liu Y, Chen X, Wu J. Efficacy of the intraoperative opioid-sparing anesthesia on quality of patients’ recovery in video-assisted thoracoscopic surgery: a randomized trial. *Journal of Thoracic Disease* 2022; **14**: 2544–55. https://doi.org/10.21037/jtd-22-50.
600. Qu G, Cui X, Liu H, Ji Z, Huang Y. Ultrasound-guided Transversus Abdominis Plane Block Improves Postoperative Analgesia and Early Recovery in Patients Undergoing Retroperitoneoscopic Urologic Surgeries: A Randomized Controlled Double-blinded Trial. *Chinese Medical Sciences Journal* 2016; **31**: 137–41. https://doi.org/10.1016/S1001-9294(16)30041-4.
601. Ra YS, Kim CH, Lee GY, Han JI. The analgesic effect of the ultrasound-guided transverse abdominis plane block after laparoscopic cholecystectomy. *Korean journal of anesthesiology* 2010; **58**: 362–8. https://doi.org/10.4097/kjae.2010.58.4.362.
602. Racle JP, Benkhadra A, Poy JY, Gleizal B, Gaudray A. Etude comparative de l’anesthésie générale et de la rachianesthésie chez la femme âgée dans la chirurgie de la hanche. *Annales Françaises d’Anesthésie et de Réanimation* 1986; **5**: 24–30. https://doi.org/10.1016/S0750-7658(86)80118-6.
603. Radtke S, Boren T, Depasquale S. Paracervical Block as a Strategy to Reduce Postoperative Pain after Laparoscopic Hysterectomy: A Randomized Controlled Trial. *Journal of Minimally Invasive Gynecology* 2019; **26**: 1164–8. https://doi.org/10.1016/j.jmig.2018.12.001.
604. Raheem MA, Badawy FA, Elsayed HM. Comparative study of analgesic effect of epidural ketamine vs. epidural tramadol in patients undergoing mastectomy under thoracic epidural anesthesia. *Egyptian Journal of Anaesthesia* 2022; **38**: 672–9. https://doi.org/10.1080/11101849.2022.2143182.
605. Rahimi M, Majedi H, Shamsi A, Eftekhar N, Kadhim RA. Comparison of incidents of nausea and vomiting between general anesthesia With Endotracheal tube and spinal anesthesia in cesarean sections*. Journal of Population Therapeutics and Clinical Pharmacology* 2023; **30**.
606. Rahimzadeh P, Faiz SHR, Salehi S, Imani F, Mueller AL, Sabouri AS. Unilateral Right-Sided Ultrasound-Guided Erector Spinae Plane Block for Post-Laparoscopic Cholecystectomy Analgesia: A Randomized Control Trial. *Anesthesiology and Pain Medicine* 2022; **12**. https://doi.org/10.5812/aapm-132152.
607. Rahimzadeh P, Mahmoudi K, Khodaverdi M, Faiz SHR. Effects of ultrasound guided ganglion stellate blockade on intraoperative and postoperative hemodynamic responses in laparoscopic gynecologic surgery. *Videosurgery and Other Miniinvasive Techniques* 2019; **15**: 351–7. https://doi.org/10.5114/wiitm.2019.89653.
608. Rahmanian A, Malekpour F, Rakei SM, Ghaffarpasand F, Mehrabani G. The Effects of Bupivacaine on Postoperative Back Pain After Lumbar Laminectomy. *Neurosurgery Quarterly* 2016; **26**: 293–7. https://doi.org/10.1097/WNQ.0000000000000185.
609. Rajan A, Tarsis S, S M, I A. A comparative evaluation of 0.75% ropivacaine with fentanyl versus 0.75% ropivacaine with dexmedetomidine in intrathecal anesthesia for lower limb surgeries. *National Journal of Physiology, Pharmacy and Pharmacology* 2024; **14**: 985-90. https://doi.org/10.5455/njppp.2024.14.03140202401042024.
610. Rani US, Panda NB, Chauhan R, Mahajan S, Kaloria N, Tripathi M. Comparison of the effects of opioid-free anesthesia (OFA) and opioid-based anesthesia (OBA) on postoperative analgesia and intraoperative hemodynamics in patients undergoing spine surgery: A prospective randomized double-blind controlled trial. *Saudi journal of anaesthesia* 2024; **18**: 173–80. https://doi.org/10.4103/sja.sja_341_23.
611. Refaie AMN, Mowafy MK. Reduction of early postoperative pain after diagnostic laparoscopy with local bupivacaine: a randomized placebo controlled study. *Middle East Fertility Society Journal* 2005; **10**.
612. Reuben S, Gutta S, Maciolek H, Sklar J. A comparison of local intraarticular anesthesia versus general anesthesia for ambulatory arthroscopic knee surgery. *Ambulatory Surgery* 2005; **12**: 39–44. https://doi.org/10.1016/j.ambsur.2004.09.001.
613. Rezniczek GA, Hecken JM, Rehman S, Dogan A, Tempfer CB, Hilal Z. Syringe or mask? Loop electrosurgical excision procedure under local or general anesthesia: a randomized trial. *American Journal of Obstetrics and Gynecology* 2020; **223**: 888.e1-888.e9. https://doi.org/10.1016/j.ajog.2020.06.041.
614. Richa F, Yazigi A, Sleilaty G, Yazbeck P. Comparison between dexmedetomidine and remifentanil for controlled hypotension during tympanoplasty. *European Journal of Anaesthesiology* 2008; **25**: 369–74. https://doi.org/10.1017/S0265021508003761.
615. Richardson MG, Dooley JW. The Effects of General Versus Epidural Anesthesia for Outpatient Extracorporeal Shock Wave Lithotripsy. *Anesthesia & Analgesia* 1998; **86**: 1214–8. https://doi.org/10.1097/00000539-199806000-00015.
616. Rickford JK, Speedy HM, Tytler JA, Lim M. Comparative evaluation of general, epidural and spinal anaesthesia for extracorporeal shockwave lithotripsy. *Annals of the Royal College of Surgeons of England* 1988; **70**: 69–73.
617. Rigamonti A, Garavaglia MM, Ma K et al. Effect of bilateral scalp nerve blocks on postoperative pain and discharge times in patients undergoing supratentorial craniotomy and general anesthesia: a randomized-controlled trial. *Canadian Journal of Anesthesia/Journal canadien d’anesthésie* 2020; **67**: 452–61. https://doi.org/10.1007/s12630-019-01558-7.
618. Ritchie ED, Tong D, Chung F, Norris AM, Miniaci A, Vairavanathan SD. Suprascapular Nerve Block for Postoperative Pain Relief in Arthroscopic Shoulder Surgery. *Anesthesia & Analgesia* 1997; **84**: 1306–12. https://doi.org/10.1097/00000539-199706000-00024.
619. Rosaeg OP, Bell M, Cicutti NJ, Dennehy KC, Lui AC, Krepski B. Pre-incision infiltration with lidocaine reduces pain and opioid consumption after reduction mammoplasty. *Regional anesthesia and pain medicine* 1998; **23**: 575–9. https://doi.org/10.1016/s1098-7339(98)90084-0.
620. Ross SB, Christodoulou M, Ross N et al. Epidural versus general anesthesia for laparo-endoscopic single-site cholecystectomy: a randomized controlled trial. *Surgical Endoscopy* 2024; **38**: 1414–21. https://doi.org/10.1007/s00464-023-10628-3.
621. Ross SB, Mangar D, Karlnoski R et al. Laparo-endoscopic single-site (LESS) cholecystectomy with epidural vs. general anesthesia. *Surgical Endoscopy* 2013; **27**: 1810–9. https://doi.org/10.1007/s00464-012-2667-8.
622. RT A, V D, S J, M R. To study the efficacy of bilateral ultrasound guided erector spinae plane block for postoperative analgesia in donor laparoscopic nephrectomy surgery. *Indian Journal of Anaesthesia* 2020; **64**: S35–40.
623. Rubod C, Hanssens S, Lucot J-P, Pougeoise M-M, Devos P, Dalmas A-F. Bloc du nerf pudendal en chirurgie périnéo-vulvaire : étude monocentrique prospective randomisée. *Gynécologie Obstétrique & Fertilité* 2015; **43**: 97–103. https://doi.org/10.1016/j.gyobfe.2014.12.006.
624. Ryu J-H, Sohn I-S, Do S-H. Controlled hypotension for middle ear surgery: a comparison between remifentanil and magnesium sulphate. *British journal of anaesthesia* 2009; **103**: 490–5. https://doi.org/10.1093/bja/aep229.
625. Sarakatsianou C, Georgopoulou S, Baloyiannis I et al. Spinal versus general anesthesia for transabdominal preperitoneal (TAPP) repair of inguinal hernia: Interim analysis of a controlled randomized trial. *The American Journal of Surgery* 2017; **214**: 239–45. https://doi.org/10.1016/j.amjsurg.2017.01.032.
626. Shen L, Yansong X, Lu J, He W. Ultrasound-guided thoracic paravertebral nerve block in patients undergoing radical mastectomy. *European Journal of Gynaecological Oncology* 2023; **44**: 90-6. https://doi.org/10.22514/ejgo.2023.083.
627. Sklika E, Kalimeris K, Perrea D, Starvropoulos N, Kostopanagiotou G, Matsota P. Remifentanil vs fentanyl during day case dental surgery in people with special needs: a comparative, pilot study of their effect on stress response and postoperative pain. *Middle East J Anaesthesiol* 2016; **5**: 509–15.
628. Hans, Sucheta, Singh Sangeeta, Nikhil Menia. COMPARATIVE STUDY OF GENERAL ANAESTHESIA VERSUS SPINAL ANAESTHESIA IN TERMS OF EFFICACY AND HEMODYNAMIC STABILITY IN PATIENTS UNDERGOING ELECTIVE LAPAROSCOPIC CHOLECYSTECTOMY. *International Journal of Academic Medicine and Pharmacy* 2022; **4**: 266-71. https://doi.org/ 10.47009/jamp.2022.4.5.53.
629. Sudré ECM, Salvador M do C, Bruno GE, Vassallo DV, Lauretti GR, Filho GNS. Remifentanil versus dexmedetomidine as coadjutants of standardized anesthetic technique in morbidly obese patients. *Revista Brasileira de Anestesiologia* 2004; **54**. https://doi.org/10.1590/S0034-70942004000200005.
630. Kalaivani V, Vinayak SP, Sreevathsa MR, Bharati VH, Yatish B. Laparoscopic Cholecystectomy Under Spinal Anaesthesia vs. General Anaesthesia: A Prospective Randomised Study. *Journal of clinical and diagnostic research : JCDR* 2014; **8**: NC01-4. https://doi.org/10.7860/JCDR/2014/9829.4700.
631. Valapala G, Ganesh V, Mrudula V. A prospective study on the comparison of general anesthesia and tea (thoracic epidural anesthesia) for perioperative outcomes in patients undergoing modified radical mastectomy. *International Journal of Research in Pharmaceutical Sciences* 2020; **11**: 7665–9.
632. Wodlin NB, Nilsson L, Kjølhede P. The impact of mode of anaesthesia on postoperative recovery from fast-track abdominal hysterectomy: a randomised clinical trial. *BJOG: An International Journal of Obstetrics & Gynaecology* 2011; **118**: 299–308. <https://doi.org/10.1111/j.1471-0528.2010.02697.x>.
633. Saboo V, Biswas D, Naz A, Seal SL, Ray R. Local infiltration of bupivacaine along the incision line following cesarean section reduces postoperative pain and analgesia requirement: a double‑blinded randomized controlled study. *J South Asian Feder Obs Gynae* 2022; **5**: 596–601. https://doi.org/10.5005/jp-journals-10006-2114
634. Sadrolsadat SH, Mahdavi AR, Moharari RS, et al. A prospective randomized trial comparing the technique of spinal and general anesthesia for lumbar disk surgery: a study of 100 cases. *Surg Neurol* 2009; **71**: 60–65. https://doi.org/10.1016/j.surneu.2008.08.003
635. Sahan M, Sarilar O, Akbulut MF, et al. Flexible ureterorenoscopy and laser lithotripsy with regional anesthesia vs general anesthesia: a prospective randomized study. *Int Braz J Urol* 2020; **46**: 1010–1018. https://doi.org/10.1590/S1677-5538.IBJU.2019.0770
636. Şahin A, Baran O. Effect of ultrasound-guided erector spinae plane block on post-surgical pain in patients undergoing nephrectomy: a single-center, randomized, double-blind, controlled trial. *J Int Med Res* 2022; **50**: 1–13. https://doi.org/10.1177/03000605221086737
637. Saini MK, Sisodia R, Gupta RC, Jethava D. Comparison of bilateral USG-guided erector spinae block versus control group for intraoperative hemodynamic stability and post-operative analgesia in spine surgeries under general anesthesia. *Asian J Pharm Clin Res* 2023; **16**: 79–83. https://doi.org/10.22159/ajpcr.2023v16i10.48708
638. Sakallı M, Ceyhan A, Yarkan Uysal H, Yazıcı I, Başar H. The efficacy of ilioinguinal and iliohypogastric nerve block for postoperative pain after caesarean section. *J Res Med Sci* 2010; **15**: 6–13.
639. Salem AE, El-Mawy MG, Al-Kholy AF. Multimodal, non-opioid based analgesia for women presented for laparoscopic hysterectomy. *Egypt J Anaesth* 2022; **38**: 139–149. https://doi.org/10.1080/11101849.2022.2031547
640. Salman N, Durukan AB, Gurbuz HA, et al. Comparison of effects of epidural bupivacaine and intravenous meperidine analgesia on patient recovery following elective abdominal aortic surgery. *Med Sci Monit* 2013; **19**: 347–352. https://doi.org/10.12659/MSM.889005
641. Salonia A, Crescenti A, Suardi N, et al. General versus spinal anesthesia in patients undergoing radical retropubic prostatectomy: results of a prospective, randomized study. *Urology* 2004; **64**: 95–100. https://doi.org/10.1016/j.urology.2004.03.010
642. Salonia A, Suardi N, Crescenti A, Colombo R, Rigatti P, Montorsi F. General versus spinal anesthesia with different forms of sedation in patients undergoing radical retropubic prostatectomy: results of a prospective, randomized study. *Int J Urol* 2006; **13**: 1185–1190. https://doi.org/10.1111/j.1442-2042.2006.01524.x
643. Salviz EA, Xu D, Frulla A, et al. Continuous interscalene block in patients having outpatient rotator cuff repair surgery: a prospective randomized trial. *Anesth Analg* 2013; **117**: 1485–1492. https://doi.org/10.1213/01.ane.0000436607.40643.0a
644. Sandhya S, Puthenveettil N, Vinodan K. Intraperitoneal nebulization of ropivacaine for control of pain after laparoscopic cholecystectomy – A randomized control trial. *J Anaesthesiol Clin Pharmacol* 2021; **37**: 443–448. https://doi.org/10.4103/joacp.JOACP_358_19
645. Sangadala P, Talawar P, Tripathy DK, Kaushal A, Gupta A, Raj N. Comparison of block characteristics and outcomes in opioid-free and opioid-based thoracic continuous spinal anaesthesia in patients undergoing major abdominal surgery: A double-blinded randomised controlled trial. *Indian J Anaesth* 2024; **68**: 280–286. https://doi.org/10.4103/ija.ija_729_23
646. Sarakat­sianou C, Baloyiannis I, Pervilotis K, Georgopoulou S, Tzovaras G. Quality of life after laparoscopic trans‑abdominal pre‑peritoneal inguinal hernia repair: spinal vs general anesthesia. *Hernia* 2021; **25**: 789–796. https://doi.org/10.1007/s10029-020-02313-4
647. Sarma R, Gupta N, Gupta A, et al. Effect of opioid-free general anesthesia versus opioid-based general anesthesia on postoperative pain and immune response in patients undergoing breast cancer surgery: A randomized controlled trial. *Indian J Surg Oncol* 2024; **15**: 901–908. https://doi.org/10.1007/s13193-024-02012-x
648. Satici MH, Tutar MS, Tire Y, et al. The effect of sacral erector spinae plane block on the quality of recovery after total hip arthroplasty: a prospective, randomized, controlled, multicenter study. *Minerva Anestesiol* 2025; **91**: 278–285. https://doi.org/10.23736/S0375-9393.24.18353-8
649. Saygı Aİ, Özdamar Ö, Gün İ, Emirkadı H, Müngen E, Akpak YK. Comparison of maternal and fetal outcomes among patients undergoing cesarean section under general and spinal anesthesia: a randomized clinical trial. *Sao Paulo Med J* 2015; **133**: 227–234. https://doi.org/10.1590/1516-3180.2014.8901012
650. Scawn RL, Allen MJ, Rose GE, Verity DH. Randomised, masked study of local anaesthesia administered prior to external dacryocystorhinostomy under general anaesthesia. *Eye (Lond)* 2019; **33**: 374–379. https://doi.org/10.1038/s41433-018-0201-5
651. Scheinin B, Rosenberg PH. Effect of prophylactic epidural morphine or bupivacaine on postoperative pain after upper abdominal surgery. *Acta Anaesthesiol Scand* 1982; **26**: 474–478. https://doi.org/10.1111/j.1399-6576.1982.tb01802.x
652. Schuitemaker RJ, Sala-Blanch X, Sánchez Cohen AP, López-Pantaleon LA, Mayoral RJ, Cubero M. Analgesic efficacy of modified pectoral block plus serratus plane block in breast augmentation surgery: A randomised, controlled, triple-blind clinical trial. *Rev Esp Anestesiol Reanim (Engl Ed)* 2019; **66**: 62–71. https://doi.org/10.1016/j.redar.2018.08.001
653. Segal D, Awad N, Nasir H, Mustafa S, Lowenstein L. Combined spinal and general anesthesia vs general anesthesia for robotic sacrocervicopexy: a randomized controlled trial. *Int Urogynecol J* 2014; **25**: 369–374. https://doi.org/10.1007/s00192-013-2194-8
654. Seki H, Furumoto K, Sato M, et al. Effects of epidural anesthesia on postoperative nausea and vomiting in laparoscopic gynecological surgery: a randomized controlled trial. *J Anesth* 2018; **32**: 608–615. https://doi.org/10.1007/s00540-018-2525-5
655. Senapathi TGA, Widnyana IM, Ariwibawa IGNM, Jaya AGPS, Janardana IMD. Combined ultrasound-guided Pecs II block and general anesthesia are effective for reducing pain from modified radical mastectomy. *J Pain Res* 2017; **10**: 1353–1358. https://doi.org/10.2147/JPR.S137637
656. Sertcakacilar G, Yildiz GO, Bayram B, Pektas Y, Cukurova Z, Hergunsel GO. Comparing preoperative anxiety effects of brachial plexus block and general anesthesia for orthopedic upper-extremity surgery: A randomized, controlled trial. *Medicina (Kaunas)* 2022; **58**: 1296. https://doi.org/10.3390/medicina58091296
657. Sessler DI, Pei L, Huang Y, et al. Recurrence of breast cancer after regional or general anaesthesia: a randomised controlled trial. *Lancet* 2019; **394**: 1807–1815. https://doi.org/10.1016/S0140-6736(19)32313-X
658. Shah R, Thapa AS, Lamichhane N, Kc SR. Safety and efficacy of spinal anaesthesia in percutaneous nephrolithotomy. *JNMA J Nepal Med Assoc* 2016; **55**: 61–66.
659. Shah SB, Chawla R, Pahade A, Mittal A, Bhargava AK, Kumar R. Comparison of pectoralis plane blocks with ketamine‑dexmedetomidine adjuncts and opioid‑based general anaesthesia in patients undergoing modified radical mastectomy. *Indian J Anaesth* 2020; **64**: 1038–1046. https://doi.org/10.4103/ija.IJA_8_20
660. Shah P, Dave U, Jadeja C. Comparative study of buprenorphine as adjuvant in PNS guided axillary brachial plexus block vs placebo. *Int J Toxicol Pharmacol Res* 2024; **14**: 129–133.
661. Shaikh M, Anwaar-ul-Hassan Khan M, Kumar K, Soomro AU, Kumar P, Siddiqui MA. Comparison of dexmedetomidine versus fentanyl in unilateral spinal anesthesia in lower limb orthopedic surgeries: randomized control trial. *J Pharm Technol Clin Pharm* 2024; **31**: 1–6.
662. Sharma S, Mehta N, Khan A. Transversus abdominis plane (TAP) block as a modality for postoperative analgesia in abdominal surgeries. *J Evol Med Dent Sci* 2017; **6**: 3385–3389.
663. Sharma S, Arora S, Jafra A, Singh G. Efficacy of erector spinae plane block for postoperative analgesia in total mastectomy and axillary clearance: a randomized controlled trial. *Saudi J Anaesth* 2020; **14**: 186–191. https://doi.org/10.4103/sja.SJA_625_19
664. Sharma S, Tiwari S, Sharma K, Nair N. Randomized controlled trial comparing the efficacy of pectoral nerve block with general anesthesia alone in patients undergoing unilateral mastectomy. *Indian J Surg Oncol* 2021; **12**: 158–163. https://doi.org/10.1007/s13193-020-01269-2
665. Sharma S, Gupta A, Mathur V, Sisodia R, Gupta S, Khandelwal S. Comparison of analgesic efficacy and duration of erector spinae block with general anaesthesia and multimodal analgesia with general anaesthesia in spine surgeries: a prospective, randomized study. *J Pharm Technol Clin Pharm* 2024; **31**: 1–6.
666. She H, Qin Y, Peng W, et al. Anterior quadratus lumborum block for total laparoscopic hysterectomy: a randomized, double-blind, placebo-controlled trial. *Clin J Pain* 2023; **39**: 571–579. https://doi.org/10.1097/AJP.0000000000001156
667. Shende D, Sadhasivam S, Madan R. Effects of peribulbar bupivacaine as an adjunct to general anaesthesia on peri-operative outcome following retinal detachment surgery. *Anaesthesia* 2000; **55**: 970–975. https://doi.org/10.1046/j.1365-2044.2000.01535.x
668. Shih M‑L, Duh Q‑Y, Hsieh C‑B, et al. Bilateral superficial cervical plexus block combined with general anesthesia administered in thyroid operations. *World J Surg* 2010; **34**: 2338–2343. https://doi.org/10.1007/s00268-010-0698-7
669. Shin H‑J, Kim S‑T, Yim K‑H, Lee H‑S, Sim J‑H, Shin Y‑D. Preemptive analgesic efficacy of ultrasound‑guided transversus abdominis plane block in patients undergoing gynecologic surgery via a transverse lower abdominal skin incision. *Korean J Anesthesiol* 2011; **61**: 413–418. https://doi.org/10.4097/kjae.2011.61.5.413
670. Shin S, Bai SJ, Rha KH, So Y, Oh YJ. The effects of combined epidural and general anesthesia on the autonomic nervous system and bioavailability of nitric oxide in patients undergoing laparoscopic pelvic surgery. *Surg Endosc* 2013; **27**: 918–926. https://doi.org/10.1007/s00464-012-2536-5
671. Shin S, Kim SH, Park KK, Kim SJ, Bae JC, Choi YS. Effects of anesthesia techniques on outcomes after hip fracture surgery in elderly patients: a prospective, randomized, controlled trial. *J Clin Med* 2020; **9**: 1605. https://doi.org/10.3390/jcm9061605
672. Shir Y, Frank SM, Brendler CB, Raja SN. Postoperative morbidity is similar in patients anesthetized with epidural and general anesthesia for radical prostatectomy. *Urology* 1994; **44**: 232–236. https://doi.org/10.1016/s0090-4295(94)80137-1
673. Shirakami G, Teratani Y, Fukuda K. Nocturnal episodic hypoxemia after ambulatory breast cancer surgery: comparison of sevoflurane and propofol–fentanyl anesthesia. *J Anesth* 2006; **20**: 78–85. https://doi.org/10.1007/s00540-005-0377-7
674. Shokri H, Ali I, Kasem A. Evaluation of the analgesic efficacy of bilateral ultrasound‑guided transversus thoracic muscle plane block on post‑sternotomy pain: a randomized controlled trial. *Local Reg Anesth* 2021; **14**: 145–152. https://doi.org/10.2147/LRA.S338685
675. Shukla U, Kumari P, Yadav JBS, Kumar A. Ultrasound guided quadratus lumborum block versus transversus abdominis plane block for postoperative analgesia in patients undergoing total abdominal hysterectomy: a RCT. *J Clin Diagn Res* 2021; **15**: 1–5. https://doi.org/10.7860/JCDR/2021/46063.14494
676. Shreeram A, Samantaray M, Prathibha VK. A prospective comparative randomized controlled study of erector spinae plane block using local anesthetics versus general anesthesia for the perioperative control of pain in patients undergoing thoracoscopic procedures. *Natl J Physiol Pharm Pharmacol* 2022; **12**: 1829–1836. https://doi.org/10.5455/njppp.2022.12.09452202229092022
677. Shyam D, Mridupaban N, Choudhury A. A clinical comparative study between general anaesthesia and spinal anaesthesia for laparoscopic cholecystectomy in elective surgeries. *JPTCP* 2023; **30**: 19.
678. Sia AT, Chow MY, Koay CK, Chong JL. Intrathecal pethidine: an alternative anaesthetic for transurethral resection of prostate? *Anaesth Intensive Care* 1997; **25**: 650–654. https://doi.org/10.1177/0310057X9702500610
679. Siam EM, Abo Aliaa DM, Elmedany S, Abdelaa ME. Erector spinae plane block combined with general anaesthesia versus conventional general anaesthesia in lumbar spine surgery. *Egypt J Anaesth* 2020; **36**: 201–226. https://doi.org/10.1080/11101849.2020.1821501
680. Siddiqui TH, Choudhary N, Kumar A, Kohli A, Wadhawan S, Bhadoria P. Comparative evaluation of dexmedetomidine and fentanyl in total intravenous anesthesia for laparoscopic cholecystectomy: a randomised controlled study. *J Anaesthesiol Clin Pharmacol* 2021; **37**: 255–260. https://doi.org/10.4103/joacp.JOACP_253_18
681. Sifaki F, Vogiatzaki T, Mantzoros I, et al. The effectiveness of ultrasound‑guided, continuous, bilateral erector spinae plane block in perioperative pain management of patients undergoing colorectal surgery: a randomized, controlled, double blind, prospective trial. *J Clin Med* 2023; **12**: 7465. https://doi.org/10.3390/jcm12237465
682. Silvestro S, Dodaro C, Cortese G, Marsilia P, Lallo C. Role of loco‑regional anaesthesia in preventing post‑operative pain in major abdominal surgery. *Acta Biomed* 2003; **74**: 89–90.
683. Simavili S, Kaygusuz I, Kinay T, Akinci Baylan A, Kafali H. Bupivacaine‑soaked absorbable gelatin sponges in caesarean section wounds: effect on postoperative pain, analgesic requirement and haemodynamic profile. *Int J Obstet Anesth* 2014; **23**: 302–308. https://doi.org/10.1016/j.ijoa.2014.07.004
684. Singelyn FJ, Lhotel L, Fabre B. Pain relief after arthroscopic shoulder surgery: a comparison of intraarticular analgesia, suprascapular nerve block, and interscalene brachial plexus block. *Anesth Analg* 2004; **99**: 589–592. https://doi.org/10.1213/01.ANE.0000125112.83117.49
685. Singh S, Kumar G, Akhileshwar. Ultrasound‑guided erector spinae plane block for postoperative analgesia in modified radical mastectomy: a randomised control study. *Indian J Anaesth* 2019; **63**: 200–204. https://doi.org/10.4103/ija.IJA_758_18
686. Singh S, Choudhary NK, Lalin D, Verma VK. Bilateral ultrasound‑guided erector spinae plane block for postoperative analgesia in lumbar spine surgery: a randomized control trial. *J Neurosurg Anesthesiol* 2020; **32**: 330–334. https://doi.org/10.1097/ANA.0000000000000603
687. Singh A, Kayina CA, Naik N, et al. Transurethral lidocaine (100 mg) bladder irrigation (TULI100) reduces the incidence of catheter related bladder discomfort in transurethral resection of bladder tumors: a randomized, double blind, controlled trial. *Int J Urol* 2023; **30**: 264–270. https://doi.org/10.1111/iju.15100
688. Sivapurapu V, Murugharaj SS, Venkata SSP. Comparison of intra-articular analgesics in arthroscopic anterior cruciate ligament reconstruction surgeries: a randomized controlled trial. *J Anaesthesiol Clin Pharmacol* 2017; **33**: 391–396. https://doi.org/10.4103/joacp.JOACP_257_16
689. Sivapurapu V, Gopal SV, Solomon A. Determination of the efficacy of ultrasound-guided bilateral transversus abdominis plane (US‑TAP) block in laparoscopic total extraperitoneal (TEP) repair of unilateral hernia surgeries: a randomized controlled trial. *J Anaesthesiol Clin Pharmacol* 2021; **37**: 475–480. https://doi.org/10.4103/joacp.JOACP_353_19
690. Slopnick EA, Sears SB, Chapman GC, et al. Pudendal nerve block analgesia at the time of vaginal surgery: a randomized, double‑blinded, sham‑controlled trial. *Urogynecology* 2023; **29**: 827–835. https://doi.org/10.1097/SPV.0000000000001351
691. Smith I, Van Hemelrijck J, White PF. Efficacy of esmolol versus alfentanil as a supplement to propofol–nitrous oxide anesthesia. *Anesth Analg* 1991; **73**: 540–546. https://doi.org/10.1213/00000539-199111000-00014
692. Smith I, Van Hemelrijck J, White PF, Shively R. Effects of local anesthesia on recovery after outpatient arthroscopy. *Anesth Analg* 1991; **73**: 536–539. https://doi.org/10.1213/00000539-199111000-00013
693. Sneyd JR, Camu F, Doenicke A, et al. Remifentanil and fentanyl during anaesthesia for major abdominal and gynaecological surgery. An open, comparative study of safety and efficacy. *Eur J Anaesthesiol* 2001; **18**: 605–614. https://doi.org/10.1046/j.1365-2346.2001.00929.x
694. Soltanimohammadi S, Seyedi M. Comparison of postoperative analgesic effect of tramadol with lidocaine when used as subcutaneous local anesthetic. *Int J Pharmacol* 2007; **3**: 207–209.
695. Soni S, Roy A, Mukherjee A, Pandey K, Mukherjee D. Efficacy of erector spinae plane block in modified radical mastectomy for postoperative analgesia: a randomised controlled study. *J Clin Diagn Res* 2024; **18**: UC33–UC37. https://doi.org/10.7860/JCDR/2024/61337.18956
696. Soudagar DH, Gandhi BP, Chittela S, Vicharapu S. Intrathecal hyperbaric bupivacaine-fentanyl versus hyperbaric bupivacaine saline in appendicectomy patients, attending the tertiary care centre, South India. *Res J Med Sci* 2024; **18**: 62–67. https://doi.org/10.36478/makrjms.2024.7.62.67
697. Soudi AM, Hammad RA, ElShafie MA, Ahmed IMS, Alhadidy MA. Comparing opioid free general anesthesia to traditional balanced general anesthesia regarding achievement of enhanced recovery in laparoscopic bariatric surgeries. *Ain-Shams J Anesthesiol* 2022; **14**: 24.
698. Sousa PCCB, Alves Bersot CD, Pereira LFG, et al. Evaluation of pectoral nerve blocks type II (PEC II) for augmentation mammoplasty: prospective, randomized, and double-blind study. *Surgeries* 2024; **5**: 1010–1022. https://doi.org/10.3390/surgeries5040081
699. Souto MM, Radaelli E, Giordani AE, Savaris A, Bassols GF. Effectiveness of local anesthetics in laparoscopic cholecystectomy: a randomized clinical trial. *Surg Laparosc Endosc Percutan Tech* 2015; **25**: 317–320. https://doi.org/10.1097/SLE.0000000000000166
700. Sprung J, Sanders MS, Warner ME, et al. Pain relief and functional status after vaginal hysterectomy: intrathecal versus general anesthesia. *Can J Anaesth* 2006; **53**: 690–700. https://doi.org/10.1007/BF03021628
701. Srejic U, Litonius E, Gandhi S, et al. Bilateral superficial trigeminal nerve blocks are not more effective than a placebo in abolishing post‑operative headache pain in pituitary transsphenoidal neurosurgery: a prospective, randomized, double‑blinded clinical trial. *Rev Recent Clin Trials* 2023; **18**: 228–237. https://doi.org/10.2174/1574887118666230227113217
702. Standl T, Eckert S, Schulteam Esch J. Postoperative complaints after spinal and thiopentone–isoflurane anaesthesia in patients undergoing orthopaedic surgery. Spinal versus general anaesthesia. *Acta Anaesthesiol Scand* 1996; **40**: 222–226. https://doi.org/10.1111/j.1399-6576.1996.tb04423.x
703. Stefanidis K, Paschopoulos M, Dusias B, Adonakis G, Lolis D. A randomized study of local or general anesthesia for laser conization of the cervix. *Arch Gynecol Obstet* 1998; **261**: 75–78. https://doi.org/10.1007/s004040050202
704. Stevens RD, Van Gessel E, Flory N, Fournier R, Gamulin Z. Lumbar plexus block reduces pain and blood loss associated with total hip arthroplasty. *Anesthesiology* 2000; **93**: 115–121. https://doi.org/10.1097/00000542-200007000-00021
705. Stewart AV, Vaghadi H, Collins L, Mitchell GW. Small-dose selective spinal anaesthesia for short-duration outpatient gynaecological laparoscopy: recovery characteristics compared with propofol anaesthesia. *Br J Anaesth* 2001; **86**: 570–572. https://doi.org/10.1093/bja/86.4.570
706. Subbiah M, Madhuwarshinee KM, Vinothan RJS, et al. A novel combined anesthetic technique to improve the surgical working conditions of lumbar and thoracolumbar spine surgery from a spine surgeon's perspective: a prospective randomized controlled study. *Asian Spine J* 2023; **17**: 285–292. https://doi.org/10.31616/asj.2022.0198
707. Subramanian V, Aggarwal S, Kale S, Parthasarathy AH, Batra A. Intraoperative superior hypogastric plexus block for postoperative pain following gynecological laparotomies. *Anaesth Pain Intensive Care* 2019; **23**: 157–161. https://doi.org/10.35975/apic.v23i2.1060
708. Sulak M, Ahiskalioglu A, Yayik A, et al. The effect of ultrasound-guided serratus plane block on the quality of life in patients undergoing modified radical mastectomy and axillary lymph node dissection: a randomized controlled study. *Anaesthesiol Intensive Ther* 2022; **54**: 48–55. https://doi.org/10.5114/ait.2022.114203
709. Sun L, Li Q, Wang Q, Ma F, Han W, Wang M. Bilateral thoracic paravertebral block combined with general anesthesia vs. general anesthesia for patients undergoing off-pump coronary artery bypass grafting: a feasibility study. *BMC Anesthesiol* 2019; **19**: 101. https://doi.org/10.1186/s12871-019-0768-9
710. Sun S, Wang C, Zhang J, Sun P. Occurrence and severity of catheter-related bladder discomfort of general anesthesia plus epidural anesthesia vs. general anesthesia in abdominal operation with urinary catheterization: a randomized, controlled study. *Front Surg* 2021; **8**: 658598. https://doi.org/10.3389/fsurg.2021.658598
711. Sun J, Wang S, Wang J, Gao X, Wang G. Effect of intravenous infusion of lidocaine compared with ultrasound-guided transverse abdominal plane block on the quality of postoperative recovery in patients undergoing laparoscopic bariatric surgery. *Drug Des Devel Ther* 2022; **16**: 739–748. https://doi.org/10.2147/DDDT.S356880
712. Sun X, Wang Q, Xia T, et al. Efficacy of ultrasound-guided bilateral costal margin block in laparoscopy-assisted gastrectomy: a double-blind randomized trial. *Anaesth Crit Care Pain Med* 2023; **42**: 101227. https://doi.org/10.1016/j.accpm.2023.101227
713. Sundarathiti P, Pasutarnchat K, Kongdan Y, Suranutkarin P. Thoracic epidural anesthesia (TEA) with 0.2% ropivacaine in combination with ipsilateral brachial plexus block (BPB) for modified radical mastectomy (MRM). *J Med Assoc Thai* 2005; **88**: 513–520.
714. Sundarathiti P, von Bormann B, Suvikapakornkul R, Lertsthichai P, Arnuntasupakul V. Paravertebral catheter for three-level injection in radical mastectomy: a randomised controlled study. *PLoS One* 2015; **10**: e0129539. https://doi.org/10.1371/journal.pone.0129539
715. Sürek A, Bozkurt MA, Ferahman S, Gemici E, Dönmez T. Laparoscopic total extraperitoneal inguinal hernia repair under epidural anesthesia versus general anesthesia. *Surg Laparosc Endosc Percutan Tech* 2020; **30**: 471–475. https://doi.org/10.1097/SLE.0000000000000817
716. Surico D, Mencaglia L, Riboni F, Vigone A, Leo L, Surico N. Minilaparotomy in spinal anaesthesia: a surgical choice in treatment of benign gynaecologic disease. *Arch Gynecol Obstet* 2010; **281**: 461–465. https://doi.org/10.1007/s00404-009-1113-1
717. Surve RM, Bansal S, Reddy M, Philip M. Use of dexmedetomidine along with local infiltration versus general anesthesia for burr hole and evacuation of chronic subdural hematoma (CSDH). *J Neurosurg Anesthesiol* 2017; **29**: 274–280. https://doi.org/10.1097/ANA.0000000000000305
718. Svircevic V, Nierich AP, Moons KG, et al. Thoracic epidural anesthesia for cardiac surgery: a randomized trial. *Anesthesiology* 2011; **114**: 262–270. https://doi.org/10.1097/ALN.0b013e318201d2de
719. Swaro S, Karan D, Banerjee S. Comparison of fentanyl and dexmedetomidine as an adjuvant to bupivacaine in supraclavicular brachial plexus block: a randomized double‑blind prospective study. *Asian J Pharm Clin Res* 2016; **9**: 74–77. https://doi.org/10.22159/ajpcr.2016.v9i5.11928
720. Szmuk P, Ezri T, Sessler DI, Stein A, Geva D. Spinal anesthesia speeds active postoperative rewarming. *Anesthesiology* 1997; **87**: 1050–1054. https://doi.org/10.1097/00000542-199711000-00007
721. Na‑Young Tae, Jin Wook Yi, Jong‑Kwon Jung, Junhyung Lee, Sooman Jo, Hyunzu Kim. A randomized comparison of multimodal analgesia and fentanyl‑based patient‑controlled analgesia in women undergoing robot‑assisted bilateral axillary breast approach thyroidectomy. *J Clin Med* 2024; **13**: 702. https://doi.org/10.3390/jcm13030702
722. Takolander R, Bergqvist D, Hulthén UL, Johansson A, Katzman PL. Carotid artery surgery. Local versus general anaesthesia as related to sympathetic activity and cardiovascular effects. *Eur J Vasc Surg* 1990; **4**: 265–270. https://doi.org/10.1016/s0950-821x(05)80205-1
723. Tan CH, Kun KY, Onsing MK, Chan MK, Chiu WKY, Tai CM. Postincisional local anaesthetic infiltration of the rectus muscle decreases early pain and morphine consumption after abdominal hysterectomy. *Acute Pain* 2002; **4**: 49–52. https://doi.org/10.1016/S1366-0071(02)00022-0
724. Tan TT, Teoh WHL, Woo DCM, Ocampo CE, Shah MK, Sia ATH. A randomised trial of the analgesic efficacy of ultrasound‑guided transversus abdominis plane block after caesarean delivery under general anaesthesia. *Eur J Anaesthesiol* 2012; **29**: 88–94. https://doi.org/10.1097/EJA.0b013e32834f015f
725. Tan WF, Guo B, Ma H, Li XQ, Fang B, Lv HW. Changes in postoperative night bispectral index of patients undergoing thoracic surgery with different types of anaesthesia management: a randomized controlled trial. *Clin Exp Pharmacol Physiol* 2016; **43**: 304–311. https://doi.org/10.1111/1440-1681.12530
726. Tan NL, Gotmaker R, Barrington MJ. Impact of local infiltration analgesia on the quality of recovery after anterior total hip arthroplasty: a randomized, triple‑blind, placebo‑controlled trial. *Anesth Analg* 2019; **129**: 1715–1722. https://doi.org/10.1213/ANE.0000000000004255
727. Tang W, Luo G, Lu Y, Chen C, Liu H, Li Y. Application of a new serratus anterior plane block in modified radical mastectomy under ultrasound guidance: a prospective, randomized controlled trial. *J Clin Anesth* 2021; **74**: 110377.<https://doi.org/10.1016/j.jclinane.2021.110377>
728. Tang L, Fang P, Fang Y, Lu Y, Xu G, Liu X. Comparison of effects between combined lumbar‑sacral plexus block plus general anesthesia and unilateral spinal anesthesia in elderly patients undergoing hip fracture surgery: a pilot randomized controlled trial. *Evid Based Complement Alternat Med* 2021; **2021**: 6685497. https://doi.org/10.1155/2021/6685497
729. Tang T, Lang F, Gao S, Chen L. Effect of combined thoracic paravertebral block and general anesthesia vs general anesthesia alone on postoperative stress and pain in patients undergoing laparoscopic radical nephrectomy. *Med Sci Monit* 2022; **28**: e933623. https://doi.org/10.12659/MSM.933623
730. Tanggaard K, Hasselager RP, Holmich ER, et al. Anterior quadratus lumborum block does not reduce postoperative opioid consumption following laparoscopic hemicolectomy: a randomized, double‑blind, controlled trial in an ERAS setting. *Reg Anesth Pain Med* 2023; **48**: 7–13. https://doi.org/10.1136/rapm-2022-103895
731. Tangpaitoon T, Nisoog C, Lojanapiwat B. Efficacy and safety of percutaneous nephrolithotomy (PCNL): a prospective and randomized study comparing regional epidural anesthesia with general anesthesia. *Int Braz J Urol* 2012; **38**: 504–511. https://doi.org/10.1590/S1677-55382012000400010
732. Taninishi H, Matsusaki T, Morimatsu H. Transversus abdominis plane block reduced early postoperative pain after robot‑assisted prostatectomy: a randomized controlled trial. *Sci Rep* 2020; **10**: 3761. https://doi.org/10.1038/s41598-020-60687-y
733. Tauber H, Streif W, Gebetsberger J, Gasteiger L, Pierer E, Knoflach M, et al. Cardiac output and cerebral blood flow during carotid surgery in regional versus general anesthesia: a prospective randomized controlled study. *J Vasc Surg* 2021; **74**: 930–937.e2. https://doi.org/10.1016/j.jvs.2021.03.042
734. Teasdale C, McCrum AM, Williams NB, Horton RE. A randomised controlled trial to compare local with general anaesthesia for short‑stay inguinal hernia repair. *Ann R Coll Surg Engl* 1982; **64**: 238–242.
735. Techanivate A, Dusitkasem S, Anuwattanavit C. Dexmedetomidine compare with fentanyl for postoperative analgesia in outpatient gynecologic laparoscopy: a randomized controlled trial. *J Med Assoc Thai* 2012; **95**: 383–390.
736. Tenling A, Joachimsson PO, Tydén H, Wegenius G, Hedenstierna G. Thoracic epidural anesthesia as an adjunct to general anesthesia for cardiac surgery: effects on ventilation–perfusion relationships. *J Cardiothorac Vasc Anesth* 1999; **13**: 258–264. https://doi.org/10.1016/s1053-0770(99)90260-4
737. Terheggen MA, Wille F, Borel Rinkes IH, Ionescu TI, Knape JT. Paravertebral blockade for minor breast surgery. *Anesth Analg* 2002; **94**: 355–359. https://doi.org/10.1097/00000539-200202000-00023
738. Terranova C, Schiavoni L, Ficarola F, et al. The role of TAP/RS block in minor gynecologic laparoscopic surgery: a randomized clinical trial. *Gynecol Obstet Invest* 2024; **89**: 103–110. https://doi.org/10.1159/000535835
739. Thagirisa A, Raheem S. Perioperative anesthetic management in patients with obesity: a multi disciplinary approach. *Res J Med Sci* 2024; **18**: 405–410. https://doi.org/10.36478/makrjms.2024.7.405.410
740. Theodoraki K, Papacharlampous P, Tsaroucha A, Vezakis A, Argyra E. The effect of transversus abdominis plane block on acute and chronic pain after inguinal hernia repair: a randomized controlled trial. *Int J Surg* 2019; **63**: 63–70. https://doi.org/10.1016/j.ijsu.2019.02.007
741. Thepsorn M, Sereeyotin J, Pannangpetch P. Effects of combined lower thoracic epidural/general anesthesia on pain control in patients undergoing elective lumbar spine surgery: a randomized controlled trial. *Spine (Phila Pa 1976)* 2018; **43**: 1381‑1385. https://doi.org/10.1097/BRS.0000000000002662
742. Thepsoparn M, Punyawattanakit P, Jaruwangsanti N, Singhatanadagige W, Chalermkitpanit P. Effects of general anesthesia with and without thoracic epidural block on length of stay after open spine surgery: a single‑blinded randomized controlled trial. *Spine J* 2022; **22**: 1694‑1699. https://doi.org/10.1016/j.spinee.2022.05.015
743. Thomas M, Philip FA, Mathew AP, Krishna KMJ. Intraoperative pectoral nerve block (Pec) for breast cancer surgery: a randomized controlled trial. *J Anaesthesiol Clin Pharmacol* 2018; **34**: 318‑323. https://doi.org/10.4103/joacp.JOACP_191_17
744. Titman S, Hommel A, Dobrydnjov I, Johansson A. The efficacy of high volume of local infiltration analgesia for postoperative pain relief after total hip arthroplasty under general anaesthesia – a randomised controlled trial. *Int J Orthop Trauma Nurs* 2018; **28**: 16‑21. https://doi.org/10.1016/j.ijotn.2017.10.003
745. Tiwari S, Chauhan A, Chatterjee P, Alam MT. Laparoscopic cholecystectomy under spinal anaesthesia: a prospective, randomised study. *J Minim Access Surg* 2013; **9**: 65‑71. https://doi.org/10.4103/0972-9941.110965
746. Tiwari A, Singh S, Singh M, Sanghwan M. Comparison of bupivacaine and 2‑chloroprocaine with and without fentanyl for subarachnoid block in inguinal hernia repair surgery: a randomised controlled study. *J Clin Diagn Res* 2021; **15**: 1‑5. https://doi.org/10.7860/JCDR/2021/46404.15353
747. Tochie JN, Bengono Bengono RS, Mbengono Metogo J, Ndikontar R, Ngouatna S, Ndom Ntock F, Minkande JZ. The efficacy and safety of an adapted opioid‑free anesthesia regimen versus conventional general anesthesia in gynecological surgery for low‑resource settings: a randomized pilot study. *BMC Anesthesiol* 2022; **22**: 325. https://doi.org/10.1186/s12871-022-01856-6
748. Toivonen J, Pitko VM, Rosenberg PH. Comparison between intra‑articular bupivacaine with epinephrine and epinephrine alone on short‑term and long‑term pain after knee arthroscopic surgery under general anesthesia in day‑surgery patients. *Acta Anaesthesiol Scand* 2002; **46**: 435‑440. https://doi.org/10.1034/j.1399-6576.2002.460418.x
749. Toleska M, Dimitrovski A. Is opioid‑free general anesthesia more superior for postoperative pain versus opioid general anesthesia in laparoscopic cholecystectomy? *Pril (Makedon Akad Nauk Umet Odd Med Nauki)* 2019; **40**: 81‑87. https://doi.org/10.2478/prilozi-2019-0018
750. Toleska M, Dimitrovski A, Toleska Dimitrovska N. Comparation among opioid‑based, low opioid and opioid free anesthesia in colorectal oncologic surgery. *Pril (Makedon Akad Nauk Umet Odd Med Nauki)* 2023; **44**: 117‑126. https://doi.org/10.2478/prilozi-2023-0013
751. Toprak H, Başaran B, Toprak ŞS, Et T, Kumru N, Korkusuz M, Bilge A, Yarımoğlu R. Efficacy of the erector spinae plane block for quality of recovery in bariatric surgery: a randomized controlled trial. *Obes Surg* 2023; **33**: 2640‑2651. https://doi.org/10.1007/s11695-023-06748-3
752. Tulgar S, Kapakli MS, Senturk O, Selvi O, Serifsoy TE, Ozer Z. Evaluation of ultrasound‑guided erector spinae plane block for postoperative analgesia in laparoscopic cholecystectomy: a prospective, randomized, controlled clinical trial. *J Clin Anesth* 2018; **49**: 101‑106. https://doi.org/10.1016/j.jclinane.2018.06.019
753. Tülübaş EK, Seyit H, Bostancı İ, et al. Laparoscopic transversus abdominal plane block is effective in multimodal analgesia for laparoscopic sleeve gastrectomy. *Bakırköy Tıp Dergisi* 2019; **15**: 198‑203. https://doi.org/10.4274/BTDMJB.galenos.2018.20180528093003
754. Tupper‑Carey DA, Fathil SM, Tan YK, Kan YM, Cheong CY, Siddiqui FJ, Assam PN. A randomised controlled trial investigating the analgesic efficacy of transversus abdominis plane block for adult laparoscopic appendicectomy. *Singapore Med J* 2017; **58**: 481‑487. https://doi.org/10.11622/smedj.2016068
755. Turkstani A, Ibraheim O, Khairy G, Alseif A, Khalil N. Spinal versus general anesthesia for laparoscopic cholecystectomy: a comparative study of cost effectiveness and side effects. *Anaesth Pain Intensive Care* 2009; **13**: 9‑14.
756. Tverskoy M, Cozacov C, Ayache M, Bradley EL Jr, Kissin I. Postoperative pain after inguinal herniorrhaphy with different types of anesthesia. *Anesth Analg* 1990; **70**: 29‑35. https://doi.org/10.1213/00000539-199001000-00006
757. Twersky RS, Jamerson B, Warner DS, Fleisher LA, Hogue S. Hemodynamics and emergence profile of remifentanil versus fentanyl prospectively compared in a large population of surgical patients. *J Clin Anesth* 2001; **13**: 407‑416. https://doi.org/10.1016/S0952-8180(01)00292-6
758. Tyagi A, Bansal A, Das S, Sethi A, Kakkar A. Effect of thoracic epidural block on infection‑induced inflammatory response: a randomized controlled trial. *J Crit Care*. 2017; **38**: 6‑12. https://doi.org/10.1016/j.jcrc.2016.10.006
759. Tziona D, Papaioannou M, Mela A, Potamianou S, Makris A. Local infiltration analgesia combined with a standardized multimodal approach including an adductor canal block in total knee arthroplasty: a prospective randomized, placebo-controlled, double-blinded clinical trial. *J Anesth*. 2018; **32**: 326‑332. https://doi.org/10.1007/s00540-018-2476-x
760. Tzovaras G, Fafoulakis F, Pratsas K, Georgopoulou S, Stamatiou G, Hatzitheofilou C. Spinal vs general anesthesia for laparoscopic cholecystectomy: interim analysis of a controlled randomized trial. *Arch Surg*. 2008; **143**: 497‑501. https://doi.org/10.1001/archsurg.143.5.497
761. Ugur B, Pirbudak L, Ozturk E, Balat O, Ugur M. Spinal versus general anesthesia in gynecologic laparoscopy: A prospective, randomized study. *Turk J Obstet Gynecol*. 2020; **17**: 186‑195. https://doi.org/10.4274/tjod.galenos.2020.28928
762. Vaida S, Ben D, Somri M, Croitoru M, Sabo E, Gaitini L. The influence of preemptive spinal anesthesia on postoperative pain. *J Clin Anesth*. 2000; **12**: 374‑377. https://doi.org/10.1016/s0952-8180(00)00174-4
763. Vallejo M, Romeo R, Davis D, Ramanathan S. Propofol–ketamine versus propofol–fentanyl for outpatient laparoscopy: comparison of postoperative nausea, emesis, analgesia, and recovery. *J Clin Anesth*. 2002; **14**: 426‑431. https://doi.org/10.1016/s0952-8180(02)00391-4
764. Valli H, Rosenberg P. Effects of three anaesthesia methods on haemodynamic responses connected with the use of thigh tourniquet in orthopaedic patients. *Acta Anaesthesiol Scand*. 1985; **29**: 142‑147. https://doi.org/10.1111/j.1399-6576.1985.tb02175.x
765. Vardon Bounes F, Pichon X, Ducos G, et al. Remifentanil for procedural sedation and analgesia in central venous catheter insertion: a randomized, controlled trial. *Clin J Pain*. 2019; **35**: 691‑695. https://doi.org/10.1097/AJP.0000000000000725
766. Varkel V, Volpin G, Ben‑David B, et al. Intraarticular fentanyl compared with morphine for pain relief following arthroscopic knee surgery. *Can J Anaesth*. 1999; **46**: 867‑871. https://doi.org/10.1007/BF03012977
767. Vasan N R, Stevenson S, Ward M. Preincisional bupivacaine in posttonsillectomy pain relief: a randomized prospective study. *Arch Otolaryngol Head Neck Surg*. 2002; **128**: 145‑149. https://doi.org/10.1001/archotol.128.2.145
768. Venkataraman R, Saravanan R, Mohana K V, Pushparani A. Evaluation of ultrasound‑guided quadratus lumborum block for post‑operative analgesia in unilateral laparoscopic renal surgeries – A randomised controlled trial. *Indian J Anaesth*. 2020; **64**: 1007‑1011. https://doi.org/10.4103/ija.IJA_335_20
769. Venkataraman R, Pushparani A, Karthik K, Nandhini P. Comparison of morphine, dexmedetomidine and dexamethasone as an adjuvant to ropivacaine in ultrasound‑guided supraclavicular brachial plexus block for postoperative analgesia—a randomized controlled trial. *J Anaesthesiol Clin Pharmacol*. 2021; **37**: 102-107. https://doi.org/10.4103/joacp.JOACP_70_19
770. Versyck B, Geffen G, Van Houwe P. Prospective double blind randomized placebo-controlled clinical trial of the pectoral nerves (Pecs) block type II. *J Clin Anesth*. 2017; **40**: 46-50. https://doi.org/10.1016/j.jclinane.2017.03.054
771. Vijayaraghavalu S, Sekar E. A comparative study on the postoperative analgesic effects of the intraperitoneal instillation of bupivacaine versus normal saline following laparoscopic cholecystectomy. *Cureus*. 2021; **13**: e14151. https://doi.org/10.7759/cureus.14151
772. Vijitpavan A, Kittikunakorn N, Komonhirun R. Comparison between intrathecal morphine and intravenous patient control analgesia for pain control after video-assisted thoracoscopic surgery: A pilot randomized controlled study. *PLoS One*. 2022; **17**: e0266324. https://doi.org/10.1371/journal.pone.0266324
773. Viviano E, Renius M, Rückert J, et al. Selective neurogenic blockade and perioperative immune reactivity in patients undergoing lung resection. *J Int Med Res*. 2012; **40**: 141‑156. https://doi.org/10.1177/147323001204000115
774. Vogt G, Heiden M, Lösche C, Lipfert P. A preoperative retrobulbar block in patients undergoing scleral buckling reduces pain, endogenous stress response, and improves vigilance. *Reg Anesth Pain Med*. 2003; **28**: 521‑527. https://doi.org/10.1016/j.rapm.2003.09.004
775. Volk T, Döpfmer UR, Schmutzler M, et al. Stress induced IL‑10 does not seem to be essential for early monocyte deactivation following cardiac surgery. *Cytokine*. 2003 Dec 21; **24**: 237‑243. https://doi.org/10.1016/s1043-4666(03)00090-5
776. Vural C, Yorukoglu D. Comparison of patient satisfaction and cost in spinal and general anesthesia for lumbar disc surgery. *Turk Neurosurg*. 2014; **24**: 380‑384. https://doi.org/10.5137/1019-5149.JTN.8575-13.0
777. Wahdan A, Seleem A. The effect of inguinal canal and intraincisional infiltration of tramadol versus bupivacaine 0.25% on postoperative pain relief in patients undergoing inguinal hernioplasty under general anesthesia. *Anaesth Pain Intensive Care*. 2017; **21**: 317‑322.
778. Wahdan A, Radwan T, Mohammed M, Mohamed A, Salama A. Effect of bilateral ultrasound-guided erector spinae blocks on postoperative pain and opioid use after lumbar spine surgery: a prospective randomized controlled trial. *J Anesth*. 2021; **35**:100‑106. https://doi.org/10.1080/11101849.2021.1893984
779. Walter C, Maxwell-Armstrong C, Pinkney T, et al. A randomised controlled trial of the efficacy of ultrasound-guided transversus abdominis plane (TAP) block in laparoscopic colorectal surgery. *Surg Endosc.* 2013; **27**: 2366‑2372. https://doi.org/10.1007/s00464-013-2791-0
780. Wang L, Cang J, Xue Z. Cost and effectiveness comparison of thoracic epidural anaesthesia combined with general anaesthesia in esophagectomy. *Biomed Res.* 2017; **28**: 2081‑2086.
781. Wang K, Zhang X, Zhang T, et al. The efficacy of ultrasound‑guided type II pectoral nerve blocks in perioperative pain management for immediate reconstruction after modified radical mastectomy: a prospective, randomized study. *Clin J Pain.* 2018; **34**: 231‑236. https://doi.org/10.1097/AJP.0000000000000529
782. Wang W, Song W, Yang C, et al. Ultrasound-guided pectoral nerve block I and serratus-intercostal plane block alleviate postoperative pain in patients undergoing modified radical mastectomy. *Pain Physician.* 2019; **22**: E315‑E323.
783. Wang B, Yan T, Kong X, Sun L, Zheng H, Zhang G. Ropivacaine infiltration analgesia of the drainage exit site enhanced analgesic effects after breast cancer surgery: a randomized controlled trial. *BMC Anesthesiol.* 2020; **20**: 257. https://doi.org/10.1186/s12871-020-01175-8
784. Wang Y, Kong L, Liu X. Effect of epidural block anesthesia combined with general anesthesia on postoperative cognitive ability of elderly patients undergoing thoracoscopic surgery. *Int J Clin Exp Pathol.* 2020; **13**: 2447‑2454. https://doi.org/10.1016/j.ijcep.2020.2447
785. Wang Q, Lin F, Huang B, Pan L. The effectiveness and safety of general and spinal anesthesia on systemic inflammatory response in patients with tumor‑type total knee arthroplasty. *Oncol Res Treat.* 2020; **43**: 428‑434. https://doi.org/10.1159/00050789
786. Wang H, Deng W, Zhu X, Fei C. Perioperative analgesia with ultrasound‑guided quadratus lumborum block for transurethral resection of prostate. *Medicine (Baltimore).* 2021; **100**: e28384. https://doi.org/10.1097/MD.0000000000028384
787. Wang X, Feng Y, Yang X, Li Z, Zhou D. Preoperative ultrasound‑guided trigeminal nerve block in orthognathic surgery: a prospective study about its efficacy of intraoperative anesthetic dosage and postoperative analgesia. *J Oral Maxillofac Surg.* 2021; **79**: 2042‑2050. https://doi.org/10.1016/j.joms.2021.04.011
788. Wang X, Jia X, Jiang Y, Li Z, Zhou Q. Opioid‑free anesthesia for postoperative recovery after video‑assisted thoracic surgery: a prospective, randomized controlled trial. *Front Surg.* 2023; **9**: 1035972. https://doi.org/10.3389/fsurg.2022.1035972
789. Wang H, Xiao Q, Luo Z, Pei F, Wang D, Zhou Z. A new cocktail formula with diprospan of local infiltration analgesia in primary total hip arthroplasty: a prospective, randomized, controlled, observer-blinded study. *Orthop Surg.* 2022; **14**: 1799‑1807. https://doi.org/10.1111/os.13288
790. Wang J, Shen Y, Guo W, et al. Propofol EC50 for inducing loss of consciousness in patients under combined epidural-general anesthesia or general anesthesia alone: a randomized double-blind study. *Front Med (Lausanne).* 2023; **10**: 1194077. https://doi.org/10.3389/fmed.2023.1194077
791. Wang L, Jiang L, Xin L, Jiang B, Chen Y, Feng Y. Effect of pecto-intercostal fascial block on extubation time in patients undergoing cardiac surgery: a randomized controlled trial. *Front Surg.* 2023; **10**: 1128691. https://doi.org/10.3389/fsurg.2023.1128691
792. Wang C, Fan S, Gu D, Deng J, Ma B, Xie L, Zhang H. Effect of ultrasound-guided transverse abdominal plane block on neutrophil-to-lymphocyte ratio, platelet-to-lymphocyte ratio, and systemic immune inflammation index in patients undergoing radical resection of endometrial carcinoma. *PLoS One.* 2024; **19**: e0315175. https://doi.org/10.1371/journal.pone.0315175
793. Wang L, Li F, Liu Y, Xiong X, Qiu Q, Wang G. The effect of preoperative topical magnesium sulfate spraying in the oropharyngeal region on postoperative sore throat following gynecological laparoscopic surgery: a randomized clinical trial. *BMC Anesthesiol.* 2025; **25**: 16. https://doi.org/10.1186/s12871-025-02893-7
794. Wang Y, Shen J, Xie R, et al. Evaluation of a precision approach to intraoperative ropivacaine nerve block to improve postoperative analgesia in 120 patients undergoing thyroid surgery. *Med Sci Monit.* 2022; **28**: e935563. https://doi.org/10.12659/MSM.935563
795. Wang Y, Zuo S, Ma Y, Shen J, Chu Q, Yang Z. Effect of ultrasound‑guided erector spinae plane block on recovery after laparoscopic sleeve gastrectomy in patients with obesity: a randomized controlled trial. *Clin Ther.* 2023; **45**: 894‑900. <https://doi.org/10.1016/j.clinthera.2023.07.010>
796. Wasfy SF, Kamhawy GA, Omar AH, Abd El Aziz HF. Bilateral continuous erector spinae block versus multimodal intravenous analgesia in coronary bypass surgery. A Randomized Trial. *Egyptian Journal of Anaesthesia*. 2021; **37**, 152–158. https://doi.org/10.1080/11101849.2021.1904548
797. Watanabe T, Moriya K, Tsubokawa N, Baba H. Effect of remifentanil on postoperative nausea and vomiting: a randomized pilot study. *J Anesth.* 2018; **32**: 781‑785. https://doi.org/10.1007/s00540-018-2550-4
798. Watters J, March R, Desai D, Monteith K, Hurtig J. Epidural anaesthesia and analgesia do not affect energy expenditure after major abdominal surgery. *Can J Anaesth.* 1993; **40**: 314‑319. https://doi.org/10.1007/BF03009628
799. Wei W, Zheng X, Gu Y, Fu W, Tang C, Yao Y. Effect of general anesthesia with thoracic paravertebral block on postoperative delirium in elderly patients undergoing thoracoscopic lobectomy: a randomized‑controlled trial. *BMC Anesthesiol.* 2022; **22**: 1. https://doi.org/10.1186/s12871-021-01532-1
800. Weng M, Wang D, Zhong J, Qian M, Zhang K, Jin Y. Comparison between esketamine and alfentanil for hysteroscopy: a prospective, double‑blind, randomized controlled trial. *Drug Des Devel Ther.* 2024; **18**: 3629‑3641. https://doi.org/10.2147/DDDT.S472651
801. Westergaard B, Jensen K, Lenz K, et al. A randomised controlled trial of ultrasound‑guided blockade of the saphenous nerve and the posterior branch of the obturator nerve for postoperative analgesia after day‑case knee arthroscopy. *Anaesthesia.* 2014; **69**: 1337‑1344. https://doi.org/10.1111/anae.12794
802. Wheatley SA, Millar JM, Jadad AR. Reduction of pain after laparoscopic sterilisation with local bupivacaine: a randomised, parallel, double-blind trial. *Br J Obstet Gynaecol.* 1994; **101**: 443-6. PMID: 8018619. https://doi.org/10.1111/j.1471-0528.1994.tb11920.x.
803. Wiesmann T, Steinfeldt T, Wagner G, Wulf H, Schmitt J, Zoremba M. Supplemental single shot femoral nerve block for total hip arthroplasty: impact on early postoperative care, pain management and lung function. *Minerva Anestesiol.* 2014; **80**:48-57. Epub 2013 Jul 23. PMID: 23877305.
804. Williams-Russo P, Sharrock N, Haas S, et al. Randomized trial of epidural versus general anesthesia: outcomes after primary total knee replacement. *Clin Orthop Relat Res.* 1996; **331**: 199-208. https://doi.org/10.1097/00003086-199610000-00028
805. Wilson S, George R, Matos J, Wilson D, Johnson W, Woolf S. Preoperative quadratus lumborum block reduces opioid requirements in the immediate postoperative period following hip arthroscopy: a randomized, blinded clinical trial. *Arthroscopy.* 2022; **38**: 808-815. https://doi.org/10.1016/j.arthro.2021.07.029
806. Wolf O, Clemens M, Purugganan R, et al. A prospective, randomized, controlled trial of paravertebral block versus general anesthesia alone for prosthetic breast reconstruction. *Plast Reconstr Surg.* 2016; **137**: 660e-666e. https://doi.org/10.1097/01.prs.0000481070.79186.0d
807. Wong S, Chan W, Fang C, et al. Infraclavicular nerve block reduces postoperative pain after distal radial fracture fixation: a randomized controlled trial. *BMC Anesthesiol.* 2020; **20**:130. https://doi.org/10.1186/s12871-020-01044-4
808. Wong H, Chen P, Tang G, et al. Deep parasternal intercostal plane block for intraoperative pain control in cardiac surgical patients for sternotomy: A prospective randomized controlled trial. *J Cardiothorac Vasc Anesth.* 2024; **38**: 683-690. https://doi.org/10.1053/j.jvca.2023.11.038
809. Wongyingsinn M, Kohmongkoludom P, Trakarnsanga A, Horthongkham N. Postoperative clinical outcomes and inflammatory markers after inguinal hernia repair using local, spinal, or general anesthesia: A randomized controlled trial. *PLoS One.* 2020; **15**: e0242925. https://doi.org/10.1371/journal.pone.0242925
810. Wu Y, Liu F, Tang H, et al. The analgesic efficacy of subcostal transversus abdominis plane block compared with thoracic epidural analgesia and intravenous opioid analgesia after radical gastrectomy. *Anesth Analg.* 2013; **117**: 507‑513. https://doi.org/10.1213/ANE.0b013e318297fcee
811. Wu C, Wu X, Yu D, Ma W, Shen C, Cao Y. A single‑dose of stellate ganglion block for the prevention of postoperative dysrhythmias in patients undergoing thoracoscopic surgery for cancer: a randomised controlled double‑blind trial. *Eur J Anaesthesiol.* 2020; **37**: 323‑331. https://doi.org/10.1097/EJA.0000000000001137
812. Wu Y, Yang W, Cai Z, Zhang Z. The effect of ultrasound‑guided low serratus anterior plane block on laparoscopic cholecystectomy postoperative analgesia: a randomized clinical trial. *Medicine (Baltimore).* 2021; **100**: e27708. https://doi.org/10.1097/MD.0000000000027708
813. Wu H, Wang S, Lv H, et al. Effect of thoracic epidural anesthesia on perioperative neutrophil extracellular trapping markers in patients undergoing anesthesia and surgery for colorectal cancer: a randomized, controlled trial. *Ann Surg Oncol.* 2023; **30**: 7561‑7568. https://doi.org/10.1245/s10434-023-14077-z
814. Wu H, Yang RZ, Chen Y, Chen T, Qiu LC, Wu XD. The effect of pericapsular nerve group (PENG) block on postoperative analgesia in elderly patients who underwent proximal femoral nail anti‑rotation surgery: a prospective, randomized‑controlled trial. *BMC Anesthesiol.* 2024; **24**: 421. https://doi.org/10.1186/s12871-024-02805-1
815. Xiao YK, She SZ, Xu LX, Zheng B. Serratus anterior plane block combined with general analgesia and patient‑controlled serratus anterior plane block in patients with breast cancer: a randomized control trial. *Adv Ther.* 2021; **38**:3444‑3454. https://doi.org/10.1007/s12325-021-01782-y
816. Xiaoqiang L, Xu­erong Z, Juan L, et al. Efficacy of pudendal nerve block for alleviation of catheter‑related bladder discomfort in male patients undergoing lower urinary tract surgeries: a randomized, controlled, double‑blind trial. *Medicine (Baltimore).* 2017; **96**: e8932. https://doi.org/10.1097/MD.0000000000008932
817. Xing JG, Abdallah FW, Brull R, Oldfield S, Dold A, Murnaghan ML, Whelan DB. Preoperative femoral nerve block for hip arthroscopy: a randomized, triple-masked controlled trial. *Am J Sports Med.* 2015; **43**: 2680‑2687. https://doi.org/10.1177/0363546515602468
818. Xing J, Wu R, Liang L, Fang H, Chen L, Luo C. Comparison of the analgesic effects of intravenous infusion of dexmedetomidine versus bilateral superficial cervical plexus block after thyroidectomy: a randomized controlled trial. *Clin J Pain.* 2021; **37**: 623‑628. https://doi.org/10.1097/AJP.0000000000000954.
819. Xu YJ, Chen WK, Zhu Y, Wang SL, Miao CH. Effect of thoracic epidural anaesthesia on serum vascular endothelial growth factor C and cytokines in patients undergoing anaesthesia and surgery for colon cancer. Br J Anaesth. 2014; **113** Suppl 1: i49‑i55. https://doi.org/10.1093/bja/aeu148
820. Xu Q, Shi NJ, Zhang H, Zhu YM. Effects of combined general–epidural anesthesia and total intravenous anesthesia on cellular immunity and prognosis in patients with non–small cell lung cancer: A comparative study. Mol Med Rep. 2017; **16**: 4445-4454. https://doi.org/10.3892/mmr.2017.7144
821. Xu YJ, Sun X, Jiang H, Yin YH, Weng ML, Sun ZR, Chen WK, Miao CH. Randomized clinical trial of continuous transversus abdominis plane block, epidural or patient-controlled analgesia for patients undergoing laparoscopic colorectal cancer surgery. Br J Surg. 2020; **107**: e133-e141. https://doi.org/10.1002/bjs.11403
822. Xu C-S, Qu X-D, Qu Z-J, Wang G, Wang H-J. Effect of subarachnoid anesthesia combined with propofol target-controlled infusion on blood loss and transfusion for posterior total hip arthroplasty in elderly patients. Chin Med J (Engl). 2020; **133**: 650-656. https://doi.org/10.1097/CM9.0000000000000688
823. Xu C, Wang F, Liu J, Zhan W, Gao C. Effect of regional versus general anaesthesia on postoperative opioid consumption, clinical outcomes and cognitive function in Chinese patients undergoing metastatic cancer surgery. Trop J Pharm Res. 2022; **21**: 879-886. https://doi.org/10.4314/tjpr.v21i4.27
824. Xu S. Effects of ultrasound-guided fascia iliaca compartment block combined with general anesthesia under tracheal intubation on the vital signs and quality of recovery of elderly patients receiving hip replacement. Int J Exp Biol. 2024; **62**: 8412. https://doi.org/10.56042/ijeb.v62i02.8412
825. Yamaguchi H, Watanabe S, Harukuni I, Hamaya Y. Effective doses of epidural morphine for relief of postcholecystectomy pain. *Anesth Analg.* 1991; **72**: 80-3. https://doi.org/10.1213/00000539-199101000-00014
826. Yan X, Liang C, Jiang J, Ji Y, Wu A, Wei C. Effects of balanced opioid-free anesthesia on postoperative nausea and vomiting in patients undergoing video-assisted thoracic surgery: a randomized trial. *BMC Anesthesiol.* 2025 Feb 8; **25**: 62. https://doi.org/10.1186/s12871-025-02938-x
827. Yang Y, Ou M, Zhou H, et al. Effect of Scalp Nerve Block with Ropivacaine on Postoperative Pain in Patients Undergoing Craniotomy: A Randomized, Double Blinded Study. *Sci Rep.* 2020; **10**(1): 2529. https://doi.org/10.1038/s41598-020-59370-z
828. Yang X, Wu Q, Wang H, Zhang Y, Chen L. Effects of Ultrasound-Guided Stellate Ganglion Block on Postoperative Quality of Recovery in Patients Undergoing Breast Cancer Surgery: A Randomized Controlled Clinical Trial. *J Healthc Eng.* 2022; **2022**: 7628183. https://doi.org/10.1155/2022/7628183
829. Yang J, Xu X, Lijun G, Yuxuan Y, Huiling L. Effect of Ropivacaine Epidural Block Combined with General Anesthesia on the Expression of Oxidative Stress and Immune Indexes in Elderly Patients with Ovarian Cancer Cell Cytoreductive Surgery. *Clin. Exp. Obstet. Gynecol.* 2024; **51**: 260. https://doi.org/10.31083/j.ceog5112260
830. Yao Y, Li J, Hu H, Xu T, Chen Y. Ultrasound‑guided serratus plane block enhances pain relief and quality of recovery after breast cancer surgery: A randomised controlled trial. *Eur J Anaesthesiol.* 2019 Jun; **36**: 436‑441. https://doi.org/10.1097/EJA.0000000000001004
831. Yao Y, Fu S, Dai S, Yun J, Zeng M,Li H, Zheng X. Impact of ultrasound‑guided erector spinae plane block on postoperative quality of recovery in video‑assisted thoracic surgery: A prospective, randomized, controlled trial. *J Clin Anesth.* 2020; **63**:109783. https://doi.org/10.1016/j.jclinane.2020.109783
832. Yao L, Dong W, Wu W, Zhao Q, Mao H. Ultrasound‑guided interscalene block versus intravenous analgesia and sedation for reduction of first anterior shoulder dislocation. *Am J Emerg Med* 2022; **56**: 232‑235. https://doi.org/10.1016/j.ajem.2022.03.047
833. Yao Y, Liu Z, Qingn W, Wang H. Application Value of Ropivacaine Combined with Sufentanil Subarachnoid Block in Patients Undergoing External Stripping and Internal Ligation of Mixed Hemorrhoids. *Int. J. Pharmacol.* 2024; **20**: 1404-1410. https://doi.org/10.3923/ijp.2024.1404.1410
834. Yaşar Ş, Yıldız A. Comparison of opioid and opioid-free anesthesia in bariatric surgery. *Ann Clin Anal Med.* 2023; **14**:825‑829. https://doi.org/10.4328/ACAM21801
835. Yazdi NA, Pestehei SK, Sanatkar M. The effect of superficial cervical plexus blockage with ultrasound guidance on pain after thyroidectomy. *J Res Med Sci* 2022; **27**: eArticle.
836. Ye X, Wang S, Wu A, et al. Comparison of the effects of general and local anesthesia in lumbar interlaminar endoscopic surgery. *Ann Palliat Med* 2020; **9**:1103‑1108. https://doi.org/10.21037/apm-20-623
837. Yeh C, Yu J, Wu C, Ho S, Chang T, Wong C. Thoracic epidural anesthesia for pain relief and postoperation recovery with modified radical mastectomy. *World J Surg* 1999; **23**:256‑260. https://doi.org/10.1007/pl00013180
838. Yeh C, Jao S, Huh B, et al. Preincisional dextromethorphan combined with thoracic epidural anesthesia and analgesia improves postoperative pain and bowel function in patients undergoing colonic surgery. *Anesth Analg* 2005; **100**:1384‑1389. https://doi.org/10.1213/01.ANE.0000148687.51613.B5
839. Simonin M, Delsuc C, Meuret P, Caruso L, Deleat‑Besson R, Lamblin A, Huriaux L, Abraham P, Bidon C, Giai J, Riche B, Rimmelé T. Hypobaric unilateral spinal anesthesia versus general anesthesia for hip fracture surgery in the elderly: a randomized controlled trial. *Anesth Analg* 2022; **135**:1262‑1270. https://doi.org/10.1213/ANE.0000000000006208
840. Simsek EM, Aksoy SM, Mantı N, Erel O, Neselioglu S, Fırat A. The effect of spinal and general anesthesia on thiol-disulfide balance during ischemia/reperfusion of the leg in patients undergoing knee replacement surgery. *JARSS* 2023; **31**: 492-498. https://doi.org/10.54875/jarss.2023.35492
841. Yilmaz F, Bas K, Zengel B. Comparative study of postoperative analgesia and opioid requirement using pectoral nerve blocks with general analgesia. *Indian J Surg* 2021; **83**: 440–445. https://doi.org/10.1007/s12262-021-02732-2
842. Yorozu T, Morisaki H, Kondoh M, Toyoda Y, Miyazawa N, Shigematsu T. Epidural anesthesia during upper abdominal surgery provides better postoperative analgesia. *J Anesth* 1996; **10**: 10–15. https://doi.org/10.1007/BF02482061
843. Yoshimoto H, Nagashima K, Sato S, Hyakumachi T, Yanagibashi Y, Masuda T. A prospective evaluation of anesthesia for posterior lumbar spine fusion: the effectiveness of preoperative epidural anesthesia with morphine. *Spine (Phila Pa 1976)* 2005; **30**: 863–869. https://doi.org/10.1097/01.brs.0000158879.26544.69
844. Yu Z, Zhang P, Wang H, Zhang L, Wei W, Fang W, Mu X. Effects of dexmedetomidine versus remifentanil on mothers and neonates during cesarean section under general anesthesia. *Biomed Pap Med Fac Univ Palacky Olomouc Czech Repub* 2020; **164**: 417–424. https://doi.org/10.5507/bp.2019.055
845. Yu JM, Tao QY, He Y, Liu D, Niu JY, Zhang Y. Opioid-free anesthesia for pain relief after laparoscopic cholecystectomy: a prospective randomized controlled trial. *J Pain Res* 2023; **16**: 3625–3632. https://doi.org/10.2147/JPR.S432601
846. Yu S, Gao G, Ma R, Lu L, Zhao Y, Yang Z. Bilateral erector spinae plane block by multiple injection for pain control in pseudomyxoma peritonei surgery: a single-blind randomized controlled trial. *BMC Anesthesiol* 2024; **24**: 370. https://doi.org/10.1186/s12871-024-02749-6
847. Yuan L, Tang W, Fu G, Wang J, Guo J, Chen W. Combining interscalene brachial plexus block with intravenous-inhalation combined anesthesia for upper extremity fractures surgery: a randomized controlled trial. *Int J Surg* 2014; **12**: 1484–1488. https://doi.org/10.1016/j.ijsu.2014.10.011
848. Yuan B, Liu D, Zhu Z, Hao Y, He K, Deng S. Effect of thoracic paravertebral nerve block on blood coagulation in patients after thoracoscopic lobectomy: a prospective randomized controlled clinical trial. *J Pain Res* 2022; **15**: 633–641. https://doi.org/10.2147/JPR.S355227
849. Zahedi H, Arbabi SH, Soltani AE, Nikoseresht M. Preemptive analgesia in elective cataract surgery (phacoemulcification). *J Res Med Sci* 2005; **10**: 63–68.
850. Zawar BP, Mehta Y, Juneja R, Arora D, Raizada A, Trehan N. Nonanalgesic benefits of combined thoracic epidural analgesia with general anesthesia in high risk elderly off pump coronary artery bypass patients. *Ann Card Anaesth* 2015; **18**: 385–391. https://doi.org/10.4103/0971-9784.159810
851. Zdravkovic M, Kamenik M. A prospective randomized controlled study of combined spinal-general anesthesia vs. general anesthesia for laparoscopic gynecological surgery: opioid sparing properties. *J Clin Anesth* 2020; **66**: 109808. https://doi.org/10.1016/j.jclinane.2020.109808
852. Zengin SU, Ergun MO, Gunal O. Effect of ultrasound-guided erector spinae plane block on postoperative pain and intraoperative opioid consumption in bariatric surgery. *Obes Surg* 2021; **31**: 5176–5182. https://doi.org/10.1007/s11695-021-05681-7
853. Zha J, Ji S, Wang C, Yang Z, Qiao S, An J. Thoracic paravertebral nerve block with ropivacaine and adjuvant dexmedetomidine produced longer analgesia in patients undergoing video-assisted thoracoscopic lobectomy: a randomized trial. *J Healthc Eng* 2021; **2021**: 1846886. https://doi.org/10.1155/2021/1846886
854. Zhan L, Er J, Zhai W, Liu J, Han J. Overall effect of sufentanil-propofol general anesthesia combined with paravertebral block on patients with lung cancer resection. *Int J Clin Exp Med* 2016; **9**: 20068–20073.
855. Zhan Y, Chen G, Huang J, Hou B, Liu W, Chen S. Effect of intercostal nerve block combined with general anesthesia on the stress response in patients undergoing minimally invasive mitral valve surgery. *Exp Ther Med* 2017; **14**: 3259–3264. https://doi.org/10.3892/etm.2017.4868
856. Zhang J, Yuan Y, Zhang Y, Wang Y. Clinical effects of single femoral nerve block in combination with general anesthesia on geriatric patients receiving total knee arthroplasty. *Pak J Med Sci* 2018; **34**: 43–48. https://doi.org/10.12669/pjms.341.14071
857. Zhang J, Liu T, Zhou H, Fei Y, Yu X. The safety and efficacy of ultrasound-guided bilateral dual transversus abdominis plane (BD-TAP) block in ERAS program of laparoscopic hepatectomy: a prospective, randomized, controlled, blinded, clinical study. *Drug Des Devel Ther* 2020; **14**: 2889–2898. https://doi.org/10.2147/DDDT.S255385
858. Zhang W, Cong X, Zhang L, et al. Effects of thoracic nerve block on perioperative lung injury, immune function, and recovery after thoracic surgery. *Clin Transl Med* 2020; **10**: e38. https://doi.org/10.1002/ctm2.38
859. Zhang TJ, Zhang JJ, Qu ZY, Zhang HY, Qiu Y, Hua Z. Bilateral erector spinae plane blocks for open posterior lumbar surgery. *J Pain Res* 2020; **13**: 709–717. https://doi.org/10.2147/JPR.S248171
860. Zhang M, Wang B, Mao W. Effect of different anesthesia methods on emergence agitation and related complications in postoperative patients with osteosarcoma. *J Healthc Eng* 2021; **2021**: 7120035. https://doi.org/10.1155/2021/7120035
861. Zhang JJ, Zhang TJ, Qu ZY, Qiu Y, Hua Z. Erector spinae plane block at lower thoracic level for analgesia in lumbar spine surgery: a randomized controlled trial. *World J Clin Cases* 2021; **9**: 5126–5134. https://doi.org/10.12998/wjcc.v9.i19.5126
862. Zhang J, Jia D, Li W, Li X, Ma Q, Chen X. General anesthesia with S-ketamine improves the early recovery and cognitive function in patients undergoing modified radical mastectomy: a prospective randomized controlled trial. *BMC Anesthesiol* 2023; **23**: 214. https://doi.org/10.1186/s12871-023-02161-6
863. Zhang Y, He K, Chen L, Ji K, Zhang Z, Wang W. Role of scalp nerve block in improving the quality of rehabilitation in patients after meningioma resection: a randomized controlled clinical trial. *Medicine (Baltimore)* 2024; **103**: e38324. https://doi.org/10.1097/MD.00000000000038324
864. Zhang R, Mai Y, Ye H, et al. A randomized controlled non-inferiority trial evaluating opioid-free versus opioid-sparing analgesia for orbital fracture reconstruction under general anesthesia. *J Pain Res* 2024; **17**: 3707–3717. https://doi.org/10.2147/JPR.S491994
865. Zhao W, Men X, Han X, Tang H, Mei B. The application of ultrasound-guided nerve block combined with general anesthesia in lower limb fracture surgery and its effect on patients’ quality of recovery from anesthesia. *Int J Clin Exp Med* 2020; **13**: 4105–4112.
866. Zhao Y, Zhang H, Song M. Clinical observation of ultrasound-guided nerve block anesthesia on postoperative pain control of fracture patients. *J Healthc Eng* 2022; **2022**: 9510669. https://doi.org/10.1155/2022/9510669
867. Zhao L, Qiu D. Ultrasound-guided femoral nerve block reduced the incidence of postoperative delirium after total knee arthroplasty: a double-blind, randomized study. *Medicine (Baltimore)* 2024; **103**: e40549. https://doi.org/10.1097/MD.00000000000040549
868. Zhao D, Wang H, Liu X, Gao Z, Sun C, Zhang Q. The efficacy of lumbar erector spinae plane block for postoperative analgesia management in patients undergoing lumbar unilateral bi-portal endoscopic surgery: a prospective randomized controlled trial. *BMC Anesthesiol* 2024; **24**: 214. https://doi.org/10.1186/s12871-024-02601-x
869. Zheng J, Pan D, Zheng B, Ruan X. Preoperative pericapsular nerve group (PENG) block for total hip arthroplasty: a randomized, placebo-controlled trial. *Reg Anesth Pain Med* 2022; **47**: 155–160. https://doi.org/10.1136/rapm-2021-103228
870. Zheng YF, Jiang YS, Liu HT, et al. Thoracic paravertebral nerve block combined laryngeal mask airway with preservation of spontaneous breathing can accelerate postoperative recovery. *Eur Rev Med Pharmacol Sci* 2023; **27**: 10875–10883. https://doi.org/10.26355/eurrev_202311_34455
871. Zhou W, Wang B, Cai X, Xu Z. Effects of epidural block anesthesia combined with general anesthesia on cognitive function and analgesic effect after thoracoscopic surgery. *Int J Clin Exp Med* 2020; **13**: 8966–8973.
872. Zhou Y, Chen B, Xiong Y, Yu X. The efficacy of ultrasound-guided superior laryngeal nerve block as an adjuvant to general anesthesia during suspension laryngoscopy vocal cord polypectomy. *Evid Based Complement Alternat Med* 2022; **2022**: 1594829. https://doi.org/10.1155/2022/1594829
873. Zhou Y, Yuan P, Xing Q, Jin W, Shi C. Efficacy of postoperative analgesia with intravenous paracetamol and mannitol injection, combined with thoracic paravertebral nerve block in post video-assisted thoracoscopic surgery pain: a prospective, randomized, double-blind controlled trial. *BMC Anesthesiol* 2024; **24**: 14. https://doi.org/10.1186/s12871-023-02386-5
874. Zhu J, Zhang XR, Yang H. Effects of combined epidural and general anesthesia on intraoperative hemodynamic responses, postoperative cellular immunity, and prognosis in patients with gallbladder cancer: a randomized controlled trial. *Medicine (Baltimore)* 2017; **96**: e6137. https://doi.org/10.1097/MD.0000000000006137
875. Zhu Q, Li L, Yang Z, et al. Ultrasound guided continuous quadratus lumborum block hastened recovery in patients undergoing open liver resection: a randomized controlled, open-label trial. *BMC Anesthesiol* 2019; **19**: 23. https://doi.org/10.1186/s12871-019-0692-z
876. Zhu L, Wang M, Wang X, Wang Y, Chen L, Li J. Changes of opioid consumption after lumbar fusion using ultrasound-guided lumbar erector spinae plane block: a randomized controlled trial. *Pain Physician* 2021; **24**: E161–E168.
877. Zhu Y, Li Z, Qin S, et al. Ultrasound-guided posterior quadratus lumborum block can reduce postoperative opioid consumption and promote rapid recovery in patients undergoing sutureless laparoscopic partial nephrectomy: a triple-blind, randomized, controlled study. *Front Oncol* 2022; **12**: 969452. https://doi.org/10.3389/fonc.2022.969452
878. Zhu K, Zheng F, Wang C, Ding L. Effect of ultrasound-guided fascia iliac compartment block on serum NLRP3 and inflammatory factors in patients with femoral intertrochanteric fracture. *Comput Math Methods Med* 2022; **2022**: 1944659. https://doi.org/10.1155/2022/1944659
879. Zhu C, Fang J, Yang J, et al. The role of ultrasound-guided multipoint fascial plane block in elderly patients undergoing combined thoracoscopic-laparoscopic esophagectomy: a prospective randomized study. *Pain Ther* 2023; **12**: 841–852. https://doi.org/10.1007/s40122-023-00514-0
880. Zhu M, Zhou R, Wang L, Ying Q. The analgesic effect of ultrasound-guided cervical erector spinae block in arthroscopic shoulder surgery: a randomized controlled clinical trial. *BMC Anesthesiol* 2024; **24**: 196. https://doi.org/10.1186/s12871-024-02586-7
881. Ziemann-Gimmel P, Goldfarb AA, Koppman J, Marema RT. Opioid-free total intravenous anaesthesia reduces postoperative nausea and vomiting in bariatric surgery beyond triple prophylaxis. *Br J Anaesth* 2014; **112**: 906–911. https://doi.org/10.1093/bja/aet551
882. Zoric L, Cuvillon P, Alonso S, et al. Single-shot intraoperative local anaesthetic infiltration does not reduce morphine consumption after total hip arthroplasty: a double-blinded placebo-controlled randomized study. *Br J Anaesth* 2014; **112**: 722–728. https://doi.org/10.1093/bja/aet439
883. Zou L, Wei Q, Pan S, Xiao F, Jiang Y, Zhong Y, Xie Y. Comparison of the effects of combined femoral and sciatic nerves block versus general anesthesia on hemodynamic stability and postoperative complication in patients with diabetic foot: a prospective, double-blind and randomized controlled trial. *Diabetes Metab Syndr Obes* 2024; **17**: 2243–2257. https://doi.org/10.2147/DMSO.S465814
884. Zullo F, Pellicano M, Zupi E, Guida M, Mastrantonio P, Nappi C. Minilaparoscopic ovarian drilling under local anesthesia in patients with polycystic ovary syndrome. *Fertil Steril* 2000; **74**: 376–379. https://doi.org/10.1016/S0015-0282(00)00611-7
885. Zupi E, Marconi D, Sbracia M, et al. Is local anesthesia an affordable alternative to general anesthesia for minilaparoscopy? *J Am Assoc Gynecol Laparosc* 2000; **7**: 111–114. https://doi.org/10.1016/S1074-3804(00)80019-5
