# Supplementary material for: Opioid‐free vs. opioid‐inclusive anaesthesia with or without regional anaesthesia for postoperative pain: a systematic review with network meta‐analysis of randomised controlled trials
Source: Anaesthesia. 2026 Jan 5;81(5):702–12. doi: 10.1111/anae.70121 (PMC13065899; doi:10.1111/anae.70121)
Supplement: Supplementary file 3 — Appendix S3. League tables for all reported outcomes comparing different anaesthetic strategies. [file ANAE-81-702-s001.docx]

# Appendix S3. League tables for all reported outcomes comparing different anaesthetic strategies. Each cell shows the estimated effect and 95% credible interval (CrI). Cells shaded in grey indicate statistically significant results (i.e., CrIs entirely above or below the null value).

## Table S1. Pain at 2 hours. League table for pain at 2 hours postoperatively. Each cell shows the mean difference and its 95% CrI. Grey shading indicates statistical significance (CrI entirely above or below 0).

| **Comparison** | **Opioid-free without RA** | **Opioid-free with RA** | **Opioid-inclusive without RA** | **Opioid-inclusive with RA** | **Remifentanil as the sole opioid without RA** | **Remifentanil as the sole opioid with RA** |
| --- | --- | --- | --- | --- | --- | --- |
| **Opioid-free without RA** | **Opioid-free without RA** | -1.734 (-2.409, -1.055) | -0.147 (-0.792, 0.494) | -1.595 (-2.251, -0.942) | 0.581 (-0.239, 1.412) | -0.933 (-1.847, -0.015) |
| **Opioid-free with RA** | 1.734 (1.055, 2.409) | **Opioid-free with RA** | 1.588 (1.072, 2.100) | 0.140 (-0.381, 0.658) | 2.312 (1.492, 3.146) | 0.800 (-0.090, 1.680) |
| **Opioid-inclusive without RA** | 0.147 (-0.494, 0.792) | -1.588 (-2.100, -1.072) | **Opioid-inclusive without RA** | -1.447 (-1.625, -1.269) | 0.727 (-0.041, 1.490) | -0.789 (-1.624, 0.048) |
| **Opioid-inclusive with RA** | 1.595 (0.942, 2.251) | -0.140 (-0.658, 0.381) | 1.447 (1.269, 1.625) | **Opioid-inclusive with RA** | 2.176 (1.404, 2.946) | 0.659 (-0.180, 1.497) |
| **Remifentanil as the sole opioid without RA** | -0.581 (-1.412, 0.239) | -2.312 (-3.146, -1.492) | -0.727 (-1.490, 0.041) | -2.176 (-2.946, -1.404) | **Remifentanil as the sole opioid without RA** | -1.516 (-2.075, -0.961) |
| **Remifentanil as the sole opioid with RA** | 0.933 (0.015, 1.847) | -0.800 (-1.680, 0.090) | 0.789 (-0.048, 1.624) | -0.659 (-1.497, 0.180) | 1.516 (0.961, 2.075) | **Remifentanil as the sole opioid with RA** |

## Table S2. Pain at 12 hours. League table for pain at 12 hours postoperatively. Each cell shows the mean difference and its 95% CrI. Grey shading indicates statistical significance (CrI entirely above or below 0).

| **Comparison** | **Opioid-free without RA** | **Opioid-free with RA** | **Opioid-inclusive without RA** | **Opioid-inclusive with RA** | **Remifentanil as the sole opioid without RA** | **Remifentanil as the sole opioid with RA** |
| --- | --- | --- | --- | --- | --- | --- |
| **Opioid-free without RA** | **Opioid-free without RA** | -0.852 (-1.412, -0.295) | 0.392 (-0.180, 0.957) | -0.559 (-1.134, 0.004) | 0.129 (-0.706, 0.950) | -1.034 (-1.865, -0.204) |
| **Opioid-free with RA** | 0.852 (0.295, 1.412) | **Opioid-free with RA** | 1.244 (0.884, 1.603) | 0.291 (-0.062, 0.647) | 0.978 (0.245, 1.723) | -0.183 (-0.918, 0.552) |
| **Opioid-inclusive without RA** | -0.392 (-0.957, 0.180) | -1.244 (-1.603, -0.884) | **Opioid-inclusive without RA** | -0.952 (-1.081, -0.822) | -0.266 (-0.922, 0.401) | -1.425 (-2.077, -0.771) |
| **Opioid-inclusive with RA** | 0.559 (-0.004, 1.134) | -0.291 (-0.647, 0.062) | 0.952 (0.822, 1.081) | **Opioid-inclusive with RA** | 0.688 (0.025, 1.355) | -0.474 (-1.127, 0.182) |
| **Remifentanil as the sole opioid without RA** | -0.129 (-0.950, 0.706) | -0.978 (-1.723, -0.245) | 0.266 (-0.401, 0.922) | -0.688 (-1.355, -0.025) | **Remifentanil as the sole opioid without RA** | -1.162 (-1.630, -0.694) |
| **Remifentanil as the sole opioid with RA** | 1.034 (0.204, 1.865) | 0.183 (-0.552, 0.918) | 1.425 (0.771, 2.077) | 0.474 (-0.182, 1.127) | 1.162 (0.694, 1.630) | **Remifentanil as the sole opioid with RA** |

## Table S3. Pain at 24 hours. League table for pain at 24 hours postoperatively. Each cell shows the mean difference and its 95% CrI. Grey shading indicates statistical significance (CrI entirely above or below 0).

| **Comparison** | **Opioid-free without RA** | **Opioid-free with RA** | **Opioid-inclusive without RA** | **Opioid-inclusive with RA** | **Remifentanil as the sole opioid without RA** | **Remifentanil as the sole opioid with RA** |
| --- | --- | --- | --- | --- | --- | --- |
| **Opioid-free without RA** | **Opioid-free without RA** | -0.522 (-0.864, -0.181) | 0.111 (-0.214, 0.436) | -0.643 (-0.972, -0.314) | 0.070 (-0.433, 0.576) | -0.641 (-1.157, -0.126) |
| **Opioid-free with RA** | 0.522 (0.181, 0.864) | **Opioid-free with RA** | 0.634 (0.398, 0.869) | -0.120 (-0.358, 0.115) | 0.592 (0.120, 1.071) | -0.121 (-0.603, 0.371) |
| **Opioid-inclusive without RA** | -0.111 (-0.436, 0.214) | -0.634 (-0.869, -0.398) | **Opioid-inclusive without RA** | -0.755 (-0.848, -0.662) | -0.041 (-0.475, 0.396) | -0.754 (-1.194, -0.304) |
| **Opioid-inclusive with RA** | 0.643 (0.314, 0.972) | 0.120 (-0.115, 0.358) | 0.755 (0.662, 0.848) | **Opioid-inclusive with RA** | 0.713 (0.285, 1.149) | 0.001 (-0.438, 0.448) |
| **Remifentanil as the sole opioid without RA** | -0.070 (-0.576, 0.433) | -0.592 (-1.071, -0.120) | 0.041 (-0.396, 0.475) | -0.713 (-1.149, -0.285) | **Remifentanil as the sole opioid without RA** | -0.712 (-1.003, -0.421) |
| **Remifentanil as the sole opioid with RA** | 0.641 (0.126, 1.157) | 0.121 (-0.371, 0.603) | 0.754 (0.304, 1.194) | -0.001 (-0.448, 0.438) | 0.712 (0.421, 1.003) | **Remifentanil as the sole opioid with RA** |

## Table S4. Pain at 48 hours. League table for pain at 48 hours postoperatively. Each cell shows the mean difference and its 95% CrI. Grey shading indicates statistical significance (CrI entirely above or below 0).

| **Comparison** | **Opioid-free without RA** | **Opioid-free with RA** | **Opioid-inclusive without RA** | **Opioid-inclusive with RA** | **Remifentanil as the sole opioid without RA** | **Remifentanil as the sole opioid with RA** |
| --- | --- | --- | --- | --- | --- | --- |
| **Opioid-free without RA** | **Opioid-free without RA** | -0.411 (-0.986, 0.157) | 0.188 (-0.317, 0.694) | -0.406 (-0.914, 0.106) | -0.069 (-0.930, 0.797) | -0.388 (-1.256, 0.481) |
| **Opioid-free with RA** | 0.411 (-0.157, 0.986) | **Opioid-free with RA** | 0.599 (0.182, 1.025) | 0.005 (-0.406, 0.426) | 0.340 (-0.492, 1.187) | 0.022 (-0.817, 0.870) |
| **Opioid-inclusive without RA** | -0.188 (-0.694, 0.317) | -0.599 (-1.025, -0.182) | **Opioid-inclusive without RA** | -0.594 (-0.740, -0.447) | -0.261 (-1.011, 0.499) | -0.579 (-1.332, 0.175) |
| **Opioid-inclusive with RA** | 0.406 (-0.106, 0.914) | -0.005 (-0.426, 0.406) | 0.594 (0.447, 0.740) | **Opioid-inclusive with RA** | 0.333 (-0.407, 1.083) | 0.017 (-0.725, 0.758) |
| **Remifentanil as the sole opioid without RA** | 0.069 (-0.797, 0.930) | -0.340 (-1.187, 0.492) | 0.261 (-0.499, 1.011) | -0.333 (-1.083, 0.407) | **Remifentanil as the sole opioid without RA** | -0.321 (-0.760, 0.124) |
| **Remifentanil as the sole opioid with RA** | 0.388 (-0.481, 1.256) | -0.022 (-0.870, 0.817) | 0.579 (-0.175, 1.332) | -0.017 (-0.758, 0.725) | 0.321 (-0.124, 0.760) | **Remifentanil as the sole opioid with RA** |

## Table S5. Opioid at 2 hours. League table for opioid consumption within 2 hours postoperatively. Each cell shows the mean difference and its 95% CrI. Grey shading indicates statistical significance (CrI entirely above or below 0).

| **Comparison** | **Opioid-free without RA** | **Opioid-free with RA** | **Opioid-inclusive without RA** | **Opioid-inclusive with RA** | **Remifentanil as the sole opioid without RA** | **Remifentanil as the sole opioid with RA** |
| --- | --- | --- | --- | --- | --- | --- |
| **Opioid-free without RA** | **Opioid-free without RA** | -4.566 (-8.981, -0.134) | 6.000 (1.729, 10.600) | 2.762 (-1.629, 7.439) | 14.468 (7.877, 21.866) | 7.835 (1.639, 14.996) |
| **Opioid-free with RA** | 4.566 (0.134, 8.981) | **Opioid-free with RA** | 10.566 (4.551, 17.000) | 7.324 (1.178, 13.837) | 19.012 (11.247, 27.723) | 12.400 (4.869, 20.887) |
| **Opioid-inclusive without RA** | -6.000 (-10.600, -1.729) | -10.566 (-17.000, -4.551) | **Opioid-inclusive without RA** | -3.243 (-4.482, -2.031) | 8.438 (2.449, 15.152) | 1.832 (-3.502, 7.918) |
| **Opioid-inclusive with RA** | -2.762 (-7.439, 1.629) | -7.324 (-13.837, -1.178) | 3.243 (2.031, 4.482) | **Opioid-inclusive with RA** | 11.691 (5.703, 18.378) | 5.070 (-0.166, 11.148) |
| **Remifentanil as the sole opioid without RA** | -14.468 (-21.866, -7.877) | -19.012 (-27.723, -11.247) | -8.438 (-15.152, -2.449) | -11.691 (-18.378, -5.703) | **Remifentanil as the sole opioid without RA** | -6.579 (-10.380, -2.874) |
| **Remifentanil as the sole opioid with RA** | -7.835 (-14.996, -1.639) | -12.400 (-20.887, -4.869) | -1.832 (-7.918, 3.502) | -5.070 (-11.148, 0.166) | 6.579 (2.874, 10.380) | **Remifentanil as the sole opioid with RA** |

## Table S6. Opioid at 12 hours. League table for opioid consumption within 12 hours postoperatively. Each cell shows the mean difference and its 95% CrI. Grey shading indicates statistical significance (CrI entirely above or below 0).

| **Comparison** | **Opioid-free without RA** | **Opioid-free with RA** | **Opioid-inclusive without RA** | **Opioid-inclusive with RA** | **Remifentanil as the sole opioid without RA** | **Remifentanil as the sole opioid with RA** |
| --- | --- | --- | --- | --- | --- | --- |
| **Opioid-free without RA** | **Opioid-free without RA** | -38.259 (-65.565, -11.774) | 20.775 (-3.848, 45.947) | 8.554 (-16.614, 34.055) | 18.929 (-10.511, 48.426) | 13.232 (-15.607, 42.822) |
| **Opioid-free with RA** | 38.259 (11.774, 65.565) | **Opioid-free with RA** | 59.116 (23.188, 96.275) | 46.959 (10.270, 84.392) | 57.253 (17.889, 97.593) | 51.582 (12.519, 92.248) |
| **Opioid-inclusive without RA** | -20.775 (-45.947, 3.848) | -59.116 (-96.275, -23.188) | **Opioid-inclusive without RA** | -12.186 (-18.207, -6.460) | -1.866 (-29.834, 25.781) | -7.456 (-31.435, 16.148) |
| **Opioid-inclusive with RA** | -8.554 (-34.055, 16.614) | -46.959 (-84.392, -10.270) | 12.186 (6.460, 18.207) | **Opioid-inclusive with RA** | 10.286 (-17.672, 38.287) | 4.738 (-19.197, 28.773) |
| **Remifentanil as the sole opioid without RA** | -18.929 (-48.426, 10.511) | -57.253 (-97.593, -17.889) | 1.866 (-25.781, 29.834) | -10.286 (-38.287, 17.672) | **Remifentanil as the sole opioid without RA** | -5.665 (-25.232, 13.898) |
| **Remifentanil as the sole opioid with RA** | -13.232 (-42.822, 15.607) | -51.582 (-92.248, -12.519) | 7.456 (-16.148, 31.435) | -4.738 (-28.773, 19.197) | 5.665 (-13.898, 25.232) | **Remifentanil as the sole opioid with RA** |

## Table S7. Opioid at 24 hours. League table for opioid consumption within 24 hours postoperatively. Each cell shows the mean difference and its 95% CrI. Grey shading indicates statistical significance (CrI entirely above or below 0).

| **Comparison** | **Opioid-free without RA** | **Opioid-free with RA** | **Opioid-inclusive without RA** | **Opioid-inclusive with RA** | **Remifentanil as the sole opioid without RA** | **Remifentanil as the sole opioid with RA** |
| --- | --- | --- | --- | --- | --- | --- |
| **Opioid-free without RA** | **Opioid-free without RA** | -9.107 (-289.962, 269.761) | -18.692 (-323.726, 288.946) | 9.130 (-298.143, 316.654) | 10.373 (-458.273, 480.677) | 2.040 (-470.685, 479.676) |
| **Opioid-free with RA** | 9.107 (-269.761, 289.962) | **Opioid-free with RA** | -9.072 (-236.217, 218.866) | 19.065 (-209.451, 246.286) | 20.037 (-422.125, 462.982) | 9.569 (-436.590, 453.254) |
| **Opioid-inclusive without RA** | 18.692 (-288.946, 323.726) | 9.072 (-218.866, 236.217) | **Opioid-inclusive without RA** | 27.246 (-49.750, 105.565) | 29.747 (-359.374, 419.685) | 19.194 (-370.075, 406.325) |
| **Opioid-inclusive with RA** | -9.130 (-316.654, 298.143) | -19.065 (-246.286, 209.451) | -27.246 (-105.565, 49.750) | **Opioid-inclusive with RA** | 1.488 (-384.274, 390.649) | -7.883 (-397.139, 380.806) |
| **Remifentanil as the sole opioid without RA** | -10.373 (-480.677, 458.273) | -20.037 (-462.982, 422.125) | -29.747 (-419.685, 359.374) | -1.488 (-390.649, 384.274) | **Remifentanil as the sole opioid without RA** | -10.271 (-253.192, 233.161) |
| **Remifentanil as the sole opioid with RA** | -2.040 (-479.676, 470.685) | -9.569 (-453.254, 436.590) | -19.194 (-406.325, 370.075) | 7.883 (-380.806, 397.139) | 10.271 (-233.161, 253.192) | **Remifentanil as the sole opioid with RA** |

## Table S8. Opioid at 48 hours. League table for opioid consumption within 48 hours postoperatively. Each cell shows the mean difference and its 95% CrI. Grey shading indicates statistical significance (CrI entirely above or below 0).

| **Comparison** | **Opioid-free without RA** | **Opioid-free with RA** | **Opioid-inclusive without RA** | **Opioid-inclusive with RA** | **Remifentanil as the sole opioid without RA** | **Remifentanil as the sole opioid with RA** |
| --- | --- | --- | --- | --- | --- | --- |
| **Opioid-free without RA** | **Opioid-free without RA** | -58.192 (-107.575, -8.886) | -8.910 (-29.781, 11.636) | -23.155 (-44.830, -2.008) | 39.237 (1.838, 77.393) | 28.315 (-10.441, 68.128) |
| **Opioid-free with RA** | 58.192 (8.886, 107.575) | **Opioid-free with RA** | 49.362 (4.066, 94.080) | 35.089 (-10.813, 80.351) | 97.630 (41.851, 154.295) | 86.587 (29.438, 143.842) |
| **Opioid-inclusive without RA** | 8.910 (-11.636, 29.781) | -49.362 (-94.080, -4.066) | **Opioid-inclusive without RA** | -14.239 (-20.462, -8.160) | 48.143 (14.617, 83.016) | 37.149 (2.019, 73.535) |
| **Opioid-inclusive with RA** | 23.155 (2.008, 44.830) | -35.089 (-80.351, 10.813) | 14.239 (8.160, 20.462) | **Opioid-inclusive with RA** | 62.359 (29.281, 97.032) | 51.392 (16.840, 87.466) |
| **Remifentanil as the sole opioid without RA** | -39.237 (-77.393, -1.838) | -97.630 (-154.295, -41.851) | -48.143 (-83.016, -14.617) | -62.359 (-97.032, -29.281) | **Remifentanil as the sole opioid without RA** | -10.951 (-28.130, 6.108) |
| **Remifentanil as the sole opioid with RA** | -28.315 (-68.128, 10.441) | -86.587 (-143.842, -29.438) | -37.149 (-73.535, -2.019) | -51.392 (-87.466, -16.840) | 10.951 (-6.108, 28.130) | **Remifentanil as the sole opioid with RA** |

## Table S9. PACU stay. League table for length of post-anaesthesia care unit (PACU) stay. Each cell shows the mean difference and its 95% CrI. Grey shading indicates statistical significance (CrI entirely above or below 0).

| **Comparison** | **Opioid-free without RA** | **Opioid-free with RA** | **Opioid-inclusive without RA** | **Opioid-inclusive with RA** | **Remifentanil as the sole opioid without RA** | **Remifentanil as the sole opioid with RA** |
| --- | --- | --- | --- | --- | --- | --- |
| **Opioid-free without RA** | **Opioid-free without RA** | -8.706 (-24.907, 7.680) | 3.760 (-8.335, 15.911) | -13.448 (-26.516, -0.495) | 1.101 (-15.555, 17.659) | -10.993 (-31.074, 9.388) |
| **Opioid-free with RA** | 8.706 (-7.680, 24.907) | **Opioid-free with RA** | 12.525 (0.522, 24.676) | -4.743 (-17.585, 8.077) | 9.890 (-7.143, 26.714) | -2.225 (-22.273, 17.618) |
| **Opioid-inclusive without RA** | -3.760 (-15.911, 8.335) | -12.525 (-24.676, -0.522) | **Opioid-inclusive without RA** | -17.225 (-23.255, -11.363) | -2.667 (-16.961, 11.598) | -14.856 (-32.695, 3.054) |
| **Opioid-inclusive with RA** | 13.448 (0.495, 26.516) | 4.743 (-8.077, 17.585) | 17.225 (11.363, 23.255) | **Opioid-inclusive with RA** | 14.564 (-0.009, 29.249) | 2.447 (-15.648, 20.542) |
| **Remifentanil as the sole opioid without RA** | -1.101 (-17.659, 15.555) | -9.890 (-26.714, 7.143) | 2.667 (-11.598, 16.961) | -14.564 (-29.249, 0.009) | **Remifentanil as the sole opioid without RA** | -12.163 (-26.352, 2.089) |
| **Remifentanil as the sole opioid with RA** | 10.993 (-9.388, 31.074) | 2.225 (-17.618, 22.273) | 14.856 (-3.054, 32.695) | -2.447 (-20.542, 15.648) | 12.163 (-2.089, 26.352) | **Remifentanil as the sole opioid with RA** |

## Table S10. Hospital LOS. League table for hospital length of stay (LOS). Each cell shows the mean difference and its 95% CrI. Grey shading indicates statistical significance (CrI entirely above or below 0).

| **Comparison** | **Opioid-free without RA** | **Opioid-free with RA** | **Opioid-inclusive without RA** | **Opioid-inclusive with RA** | **Remifentanil as the sole opioid without RA** | **Remifentanil as the sole opioid with RA** |
| --- | --- | --- | --- | --- | --- | --- |
| **Opioid-free without RA** | **Opioid-free without RA** | -6.349 (-45.924, 33.669) | 4.862 (-31.820, 41.600) | -10.767 (-48.519, 26.493) | -13.931 (-62.519, 34.164) | -27.901 (-82.636, 26.993) |
| **Opioid-free with RA** | 6.349 (-33.669, 45.924) | **Opioid-free with RA** | 11.182 (-14.187, 36.596) | -4.520 (-30.710, 21.602) | -7.667 (-44.727, 28.990) | -21.620 (-66.192, 23.371) |
| **Opioid-inclusive without RA** | -4.862 (-41.600, 31.820) | -11.182 (-36.596, 14.187) | **Opioid-inclusive without RA** | -15.674 (-26.476, -5.041) | -18.889 (-56.326, 18.103) | -32.658 (-77.167, 11.654) |
| **Opioid-inclusive with RA** | 10.767 (-26.493, 48.519) | 4.520 (-21.602, 30.710) | 15.674 (5.041, 26.476) | **Opioid-inclusive with RA** | -3.311 (-40.975, 34.192) | -16.978 (-61.736, 27.822) |
| **Remifentanil as the sole opioid without RA** | 13.931 (-34.164, 62.519) | 7.667 (-28.990, 44.727) | 18.889 (-18.103, 56.326) | 3.311 (-34.192, 40.975) | **Remifentanil as the sole opioid without RA** | -13.872 (-46.211, 18.444) |
| **Remifentanil as the sole opioid with RA** | 27.901 (-26.993, 82.636) | 21.620 (-23.371, 66.192) | 32.658 (-11.654, 77.167) | 16.978 (-27.822, 61.736) | 13.872 (-18.444, 46.211) | **Remifentanil as the sole opioid with RA** |

## Table S11. PONV. League table for postoperative nausea and vomiting (PONV). Each cell shows the odds ratio and its 95% CrI. Grey shading indicates statistical significance (CrI entirely above or below 1).

| **Comparison** | **Opioid-free without RA** | **Opioid-free with RA** | **Opioid-inclusive without RA** | **Opioid-inclusive with RA** | **Remifentanil as the sole opioid without RA** | **Remifentanil as the sole opioid with RA** |
| --- | --- | --- | --- | --- | --- | --- |
| **Opioid-free without RA** | **Opioid-free without RA** | 0.628 (0.425, 0.922) | 2.616 (1.841, 3.721) | 1.162 (0.810, 1.671) | 2.392 (1.487, 3.858) | 1.214 (0.706, 2.086) |
| **Opioid-free with RA** | 1.593 (1.085, 2.353) | **Opioid-free with RA** | 4.169 (3.202, 5.454) | 1.852 (1.416, 2.433) | 3.812 (2.404, 6.051) | 1.934 (1.152, 3.221) |
| **Opioid-inclusive without RA** | 0.382 (0.269, 0.543) | 0.240 (0.183, 0.312) | **Opioid-inclusive without RA** | 0.444 (0.391, 0.505) | 0.914 (0.604, 1.388) | 0.464 (0.288, 0.743) |
| **Opioid-inclusive with RA** | 0.860 (0.598, 1.235) | 0.540 (0.411, 0.706) | 2.250 (1.981, 2.561) | **Opioid-inclusive with RA** | 2.058 (1.353, 3.158) | 1.043 (0.648, 1.685) |
| **Remifentanil as the sole opioid without RA** | 0.418 (0.259, 0.672) | 0.262 (0.165, 0.416) | 1.094 (0.720, 1.655) | 0.486 (0.317, 0.739) | **Remifentanil as the sole opioid without RA** | 0.507 (0.346, 0.743) |
| **Remifentanil as the sole opioid with RA** | 0.824 (0.479, 1.416) | 0.517 (0.310, 0.868) | 2.155 (1.347, 3.468) | 0.958 (0.593, 1.544) | 1.974 (1.346, 2.893) | **Remifentanil as the sole opioid with RA** |

## Table S12. Dizziness. League table for postoperative dizziness. Each cell shows the odds ratio and its 95% CrI. Grey shading indicates statistical significance (CrI entirely above or below 1).

| **Comparison** | **Opioid-free without RA** | **Opioid-free with RA** | **Opioid-inclusive without RA** | **Opioid-inclusive with RA** | **Remifentanil as the sole opioid without RA** | **Remifentanil as the sole opioid with RA** |
| --- | --- | --- | --- | --- | --- | --- |
| **Opioid-free without RA** | **Opioid-free without RA** | 0.286 (0.057, 1.485) | 0.989 (0.322, 3.573) | 0.385 (0.113, 1.446) | 14.644 (2.908, 94.542) | 5.654 (0.770, 45.948) |
| **Opioid-free with RA** | 3.492 (0.673, 17.519) | **Opioid-free with RA** | 3.468 (1.219, 11.189) | 1.341 (0.476, 4.098) | 52.031 (4.969, 592.201) | 20.120 (1.409, 262.995) |
| **Opioid-inclusive without RA** | 1.011 (0.280, 3.103) | 0.288 (0.089, 0.820) | **Opioid-inclusive without RA** | 0.388 (0.241, 0.592) | 15.029 (1.806, 127.603) | 5.793 (0.496, 58.451) |
| **Opioid-inclusive with RA** | 2.599 (0.692, 8.832) | 0.746 (0.244, 2.099) | 2.576 (1.690, 4.142) | **Opioid-inclusive with RA** | 38.851 (4.677, 345.242) | 14.939 (1.282, 157.776) |
| **Remifentanil as the sole opioid without RA** | 0.068 (0.011, 0.344) | 0.019 (0.002, 0.201) | 0.067 (0.008, 0.554) | 0.026 (0.003, 0.214) | **Remifentanil as the sole opioid without RA** | 0.386 (0.128, 0.972) |
| **Remifentanil as the sole opioid with RA** | 0.177 (0.022, 1.298) | 0.050 (0.004, 0.710) | 0.173 (0.017, 2.015) | 0.067 (0.006, 0.780) | 2.589 (1.028, 7.791) | **Remifentanil as the sole opioid with RA** |

## Table S13. Pruritus. League table for postoperative pruritus. Each cell shows the odds ratio and its 95% CrI. Grey shading indicates statistical significance (CrI entirely above or below 1).

| **Comparison** | **Opioid-free without RA** | **Opioid-free with RA** | **Opioid-inclusive without RA** | **Opioid-inclusive with RA** | **Remifentanil as the sole opioid without RA** | **Remifentanil as the sole opioid with RA** |
| --- | --- | --- | --- | --- | --- | --- |
| **Opioid-free without RA** | **Opioid-free without RA** | 1.487 (0.130, 19.670) | 10.389 (1.001, 157.493) | 11.133 (1.078, 168.071) | 6.251 (0.209, 296.293) | 9.581 (0.286, 468.461) |
| **Opioid-free with RA** | 0.673 (0.051, 7.687) | **Opioid-free with RA** | 7.079 (2.044, 29.554) | 7.580 (2.437, 28.052) | 4.287 (0.220, 109.807) | 6.467 (0.293, 173.602) |
| **Opioid-inclusive without RA** | 0.096 (0.006, 0.999) | 0.141 (0.034, 0.489) | **Opioid-inclusive without RA** | 1.066 (0.497, 2.283) | 0.606 (0.029, 13.198) | 0.905 (0.042, 21.079) |
| **Opioid-inclusive with RA** | 0.090 (0.006, 0.927) | 0.132 (0.036, 0.410) | 0.938 (0.438, 2.012) | **Opioid-inclusive with RA** | 0.573 (0.030, 11.330) | 0.854 (0.042, 18.097) |
| **Remifentanil as the sole opioid without RA** | 0.160 (0.003, 4.786) | 0.233 (0.009, 4.535) | 1.649 (0.076, 34.408) | 1.745 (0.088, 33.821) | **Remifentanil as the sole opioid without RA** | 1.493 (0.253, 9.413) |
| **Remifentanil as the sole opioid with RA** | 0.104 (0.002, 3.494) | 0.155 (0.006, 3.415) | 1.104 (0.047, 23.950) | 1.171 (0.055, 23.795) | 0.670 (0.106, 3.955) | **Remifentanil as the sole opioid with RA** |

## Table S14. Urinary retention. League table for postoperative urinary retention. Each cell shows the odds ratio and its 95% CrI. Grey shading indicates statistical significance (CrI entirely above or below 1).

| **Comparison** | **Opioid-free without RA** | **Opioid-free with RA** | **Opioid-inclusive without RA** | **Opioid-inclusive with RA** | **Remifentanil as the sole opioid without RA** | **Remifentanil as the sole opioid with RA** |
| --- | --- | --- | --- | --- | --- | --- |
| **Opioid-free without RA** | **Opioid-free without RA** | 42633444782564928 (9995.624, 4.329e+37) | 134093111172919088 (27953.801, 1.416e+38) | 143806524395097776 (28531.115, 1.460e+38) | 1905032.233 (0.920, 2.707e+22) | 734586.009 (0.195, 1.125e+22) |
| **Opioid-free with RA** | 0 (0, 0) | **Opioid-free with RA** | 3.037 (0.214, 58.195) | 3.171 (0.168, 88.569) | 0 (0, 0.131) | 0 (0, 0.079) |
| **Opioid-inclusive without RA** | 0 (0, 0) | 0.329 (0.017, 4.670) | **Opioid-inclusive without RA** | 1.042 (0.269, 4.500) | 0 (0, 0.055) | 0 (0, 0.033) |
| **Opioid-inclusive with RA** | 0 (0, 0) | 0.315 (0.011, 5.965) | 0.960 (0.222, 3.720) | **Opioid-inclusive with RA** | 0 (0, 0.057) | 0 (0, 0.033) |
| **Remifentanil as the sole opioid without RA** | 0 (0, 1.087) | 331920415.312 (7.644, 2.375e+27) | 1102400343.199 (18.054, 7.643e+27) | 1146389017.047 (17.502, 8.272e+27) | **Remifentanil as the sole opioid without RA** | 0.361 (0.013, 11.431) |
| **Remifentanil as the sole opioid with RA** | 0 (0, 5.134) | 951146934.007 (12.694, 7.911e+27) | 3125871770.226 (30.568, 2.458e+28) | 3318304068.679 (29.878, 2.753e+28) | 2.769 (0.087, 75.357) | **Remifentanil as the sole opioid with RA** |
