# Supplementary material for: Opioid‐free vs. opioid‐inclusive anaesthesia with or without regional anaesthesia for postoperative pain: a systematic review with network meta‐analysis of randomised controlled trials
Source: Anaesthesia. 2026 Jan 5;81(5):702–12. doi: 10.1111/anae.70121 (PMC13065899; doi:10.1111/anae.70121)
Supplement: Supplementary file 5 — Appendix S5. Summary of network meta‐analysis findings and certainty of evidence assessments for all 14 postoperative outcomes analysed. [file ANAE-81-702-s005.docx]

**Appendix S5.** Summary of network meta-analysis findings and certainty of evidence assessments for all 14 postoperative outcomes analysed. This includes pain intensity at 2, 12, 24, and 48 hours; opioid consumption at corresponding time points; length of stay in the post-anaesthesia care unit (PACU) and in hospital; and the incidence of postoperative nausea and vomiting (PONV), dizziness, pruritus, and urinary retention. Certainty of evidence for each comparison was assessed using the GRADE framework in conjunction with the CINeMA approach. RA, regional anaesthesia.

**Table S1.** Summary of network meta-analysis findings for pain at 2 hours postoperatively. Techniques are listed in descending order of SUCRA values. Pain was measured on a numerical rating scale from 0 to 10**,** with higher scores indicating more intense pain.

| Intervention | Number of studies n = 486 | Number of participants n = 15845 | Mean difference (95% credible interval) | Certainty of evidence | Median ranking (95% credible interval) |
| --- | --- | --- | --- | --- | --- |
| Opioid-free with RA | n = 26 | n = 834 | -0.140 (-0.658, 0.381) | ✚✚◯◯ Low Due to concerns about imprecision and heterogeneity | 1 (1–3) |
| Opioid-inclusive with RA | n = 192 | n = 6626 | Reference | Reference | 2 (1–3) |
| Remifentanil as the sole opioid with RA | n = 22 | n = 699 | 0.659 (-0.180, 1.497) | ✚✚◯◯ Low Due to concerns about imprecision and heterogeneity | 3 (2–4) |
| Opioid-inclusive without RA | n = 202 | n = 6324 | 1.447 (1.269, 1.625) | ✚✚✚◯ Moderate Due to concerns about heterogeneity | 4 (3–5) |
| Opioid-free without RA | n = 18 | n = 554 | 1.595 (0.942, 2.251) | ✚✚✚◯ Moderate Due to concerns about heterogeneity | 5 (4–6) |
| Remifentanil as the sole opioid without RA | n = 26 | n = 808 | 2.176 (1.404, 2.946) | ✚✚◯◯ Low Due to concerns about imprecision and heterogeneity | 6 (5–6) |

**Table S2.** Summary of network meta-analysis findings for pain at 12 hours postoperatively. Techniques are listed in descending order of SUCRA values. Pain was measured on a numerical rating scale from 0 to 10**,** with higher scores indicating more intense pain.

| Intervention | Number of studies n = 524 | Number of participants n = 16242 | Mean difference (95% credible interval) | Certainty of evidence | Median ranking (95% credible interval) |
| --- | --- | --- | --- | --- | --- |
| Remifentanil as the sole opioid with RA | n = 35 | n = 1204 | -0.474 (-1.127, 0.182) | ✚✚◯◯ Low Due to concerns about imprecision, heterogeneity, and publication bias | 1 (1–3) |
| Opioid-free with RA | n = 33 | n = 1092 | -0.291 (-0.647, 0.062) | ✚✚◯◯ Low Due to concerns about imprecision, heterogeneity, and publication bias | 2 (1–3) |
| Opioid-inclusive with RA | n = 230 | n = 8021 | Reference | Reference | 3 (2–4) |
| Opioid-free without RA | n = 29 | n = 930 | 0.559 (-0.004, 1.134) | ✚✚◯◯ Low Due to concerns about imprecision, heterogeneity, and publication bias | 4 (3–6) |
| Remifentanil as the sole opioid without RA | n = 38 | n = 1208 | 0.688 (0.025, 1.355) | ✚✚◯◯ Low Due to concerns about imprecision, heterogeneity, and publication bias | 5 (4–6) |
| Opioid-inclusive without RA | n = 234 | n = 8190 | 0.952 (0.822, 1.081) | ✚✚◯◯ Low Due to concerns about heterogeneity and publication bias | 6 (5–6) |

**Table S3.** Summary of network meta-analysis findings for pain at 24 hours postoperatively. Techniques are listed in descending order of SUCRA values. Pain was measured on a numerical rating scale from 0 to 10**,** with higher scores indicating more intense pain.

| Intervention | Number of studies n = 465 | Number of participants n = 35700 | Mean difference (95% credible interval) | Certainty of evidence | Median ranking (95% credible interval) |
| --- | --- | --- | --- | --- | --- |
| Opioid-inclusive with RA | n = 388 | n = 14340 | Reference | Reference | 2 (1–3) |
| Remifentanil as the sole opioid with RA | n = 41 | n = 1407 | 0.001 (-0.438, 0.448) | ✚✚◯◯ Low Due to concerns about imprecision and heterogeneity | 2 (1–3) |
| Opioid-free with RA | n = 64 | n = 2528 | 0.120 (-0.115, 0.358) | ✚✚◯◯ Low Due to concerns about imprecision and heterogeneity | 3 (1–3) |
| Opioid-free without RA | n = 34 | n = 1503 | 0.643 (0.314, 0.972) | ✚✚✚◯  Moderate Due to concerns about heterogeneity | 4 (4–6) |
| Remifentanil as the sole opioid without RA | n = 43 | n = 1386 | 0.713 (0.285, 1.149) | ✚✚✚◯  Moderate Due to concerns about heterogeneity | 5 (4–6) |
| Opioid-inclusive without RA | n = 375 | n = 14536 | 0.755 (0.662, 0.848) | ✚✚✚◯  Moderate Due to concerns about heterogeneity | 6 (5–6) |

**Table S4.** Summary of network meta-analysis findings for pain at 48 hours postoperatively. Techniques are listed in descending order of SUCRA values. Pain was measured on a numerical rating scale from 0 to 10**,** with higher scores indicating more intense pain.

| Intervention | Number of studies n = 170 | Number of participants n = 12954 | Mean difference (95% credible interval) | Certainty of evidence | Median ranking (95% credible interval) |
| --- | --- | --- | --- | --- | --- |
| Opioid-inclusive with RA | n = 140 | n = 5590 | Reference | Reference | 2 (1–4) |
| Opioid-free with RA | n = 18 | n = 589 | -0.005 (-0.426, 0.406) | ✚✚◯◯ Low Due to concerns about imprecision and heterogeneity | 2 (1–5) |
| Remifentanil as the sole opioid with RA | n = 16 | n = 518 | 0.017 (-0.725, 0.758) | ✚✚◯◯ Low Due to concerns about imprecision and heterogeneity | 2 (1–5) |
| Remifentanil as the sole opioid without RA | n = 16 | n = 512 | 0.333 (-0.407, 1.083) | ✚✚✚◯  Moderate Due to concerns about heterogeneity | 4 (2–6) |
| Opioid-free without RA | n = 13 | n = 514 | 0.406 (-0.106, 0.914) | ✚✚✚◯  Moderate Due to concerns about heterogeneity | 5 (2–6) |
| Opioid-inclusive without RA | n = 138 | n = 5231 | 0.594 (0.447, 0.740) | ✚✚✚◯  Moderate Due to concerns about heterogeneity | 6 (6–6) |

**Table S5.** Summary of network meta-analysis findings for opioid consumption at 2 hours postoperatively. Techniques are listed in descending order of SUCRA values. Opioid doses were converted to intravenous morphine-equivalent doses (mg) to enable standardised comparisons across studies.

| Intervention | Number of studies n = 37 | Number of participants n = 2237 | Mean difference (95% credible interval) | Certainty of evidence | Median ranking (95% credible interval) |
| --- | --- | --- | --- | --- | --- |
| Opioid-free with RA | n = 2 | n = 63 | -7.324 (-13.837, -1.178) | ✚✚✚◯  Moderate Due to concerns about heterogeneity and publication bias | 1 (1–2) |
| Opioid-free without RA | n = 5 | n = 198 | -2.762 (-7.439, 1.629) | ✚✚◯◯  Low Due to concerns about imprecision, heterogeneity, and publication bias | 2 (2–3) |
| Opioid-inclusive with RA | n = 29 | n = 910 | Reference | Reference | 3 (2–4) |
| Opioid-inclusive without RA | n = 30 | n = 913 | 3.243 (2.031, 4.482) | ✚✚✚◯  Moderate Due to concerns about heterogeneity and publication bias | 4 (4–5) |
| Remifentanil as the sole opioid with RA | n = 4 | n = 78 | 5.070 (-0.166, 11.148) | ✚✚◯◯  Low Due to concerns about imprecision, heterogeneity, and publication bias | 5 (5–5) |
| Remifentanil as the sole opioid without RA | n = 4 | n = 75 | 11.691 (5.703, 18.378) | ✚✚✚◯  Moderate Due to concerns about heterogeneity and publication bias | 6 (6–6) |

**Table S6.** Summary of network meta-analysis findings for opioid consumption at 12 hours postoperatively. Techniques are listed in descending order of SUCRA values. Opioid doses were converted to intravenous morphine-equivalent doses (mg) to enable standardised comparisons across studies.

| Intervention | Number of studies n = 50 | Number of participants n = 3439 | Mean difference (95% credible interval) | Certainty of evidence | Median ranking (95% credible interval) |
| --- | --- | --- | --- | --- | --- |
| Opioid-free with RA | n = 2 | n = 68 | -46.959 (-84.392, -10.270) | ✚✚◯◯  Low  Due to concerns about heterogeneity and imprecision | 1 (1–1) |
| Opioid-free without RA | n = 5 | n = 193 | -8.554 (-34.055, 16.614) | ✚✚◯◯  Low Due to concerns about heterogeneity and imprecision | 2 (2–5) |
| Opioid-inclusive with RA | n = 41 | n = 1485 | Reference | ✚✚◯◯  Low Due to concerns about heterogeneity and imprecision | 3 (2–5) |
| Remifentanil as the sole opioid with RA | n = 5 | n = 156 | 4.738 (-19.197, 28.773) | ✚✚◯◯  Low Due to concerns about heterogeneity and imprecision | 4 (2–6) |
| Remifentanil as the sole opioid without RA | n = 4 | n = 99 | 10.286 (-17.672, 38.287) | ✚✚◯◯  Low Due to concerns about heterogeneity and imprecision | 5 (2–6) |
| Opioid-inclusive without RA | n = 43 | n = 1438 | 12.186 (6.460, 18.207) | Reference | 6 (6–6) |

**Table S7.** Summary of network meta-analysis findings for opioid consumption at 24 hours postoperatively. Techniques are listed in descending order of SUCRA values. Opioid doses were converted to intravenous morphine-equivalent doses (mg) to enable standardised comparisons across studies.

| Intervention | Number of studies n = 278 | Number of participants n = 18338 | Mean difference (95% credible interval) | Certainty of evidence | Median ranking (95% credible interval) |
| --- | --- | --- | --- | --- | --- |
| Opioid-inclusive without RA | n = 228 | n = 7393 | -27.246 (-105.565, 49.750) | Reference | 3 (1–6) |
| Opioid-free with RA | n = 34 | n = 1300 | -19.065 (-246.286, 209.451) | ✚✚◯◯  Low Due to concerns about heterogeneity and imprecision | 3 (1–6) |
| Remifentanil as the sole opioid with RA | n = 25 | n = 685 | -7.883 (-397.139, 380.806) | ✚✚◯◯  Low Due to concerns about heterogeneity and imprecision | 4 (1–6) |
| Opioid-free without RA | n = 18 | n = 500 | -9.130 (-316.654, 298.143) | ✚✚◯◯  Low Due to concerns about heterogeneity and imprecision | 4 (1–6) |
| Remifentanil as the sole opioid without RA | n = 25 | n = 650 | 1.488 (-384.274, 390.649) | ✚✚◯◯  Low Due to concerns about heterogeneity and imprecision | 4 (1–6) |
| Opioid-inclusive with RA | n = 229 | n = 7810 | Reference | ✚✚◯◯  Low Due to concerns about heterogeneity and imprecision | 4 (1–6) |

**Table S8.** Summary of network meta-analysis findings for opioid consumption at 48 hours postoperatively. Techniques are listed in descending order of SUCRA values. Opioid doses were converted to intravenous morphine-equivalent doses (mg) to enable standardised comparisons across studies.

| Intervention | Number of studies n = 75 | Number of participants n = 5247 | Mean difference (95% credible interval) | Certainty of evidence* | Median ranking (95% credible interval) |
| --- | --- | --- | --- | --- | --- |
| Opioid-free with RA | n = 1 | n = 51 | -35.089 (-80.351, 10.813) | ✚✚◯◯  Low Due to concerns about heterogeneity, imprecision, and publication bias | 1 (1–2) |
| Opioid-inclusive with RA | n = 61 | n = 2167 | Reference | Reference | 2 (1–2) |
| Opioid-inclusive without RA | n = 65 | n = 2225 | 14.239 (8.160, 20.462) | ✚✚◯◯  Low Due to concerns about heterogeneity, imprecision, and publication bias | 3 (3–4) |
| Opioid-free without RA | n = 6 | n = 343 | 23.155 (2.008, 44.830) | ✚✚◯◯  Low Due to concerns about heterogeneity, imprecision, and publication bias | 4 (3–5) |
| Remifentanil as the sole opioid with RA | n = 8 | n = 227 | 51.392 (16.840, 87.466) | ✚✚◯◯  Low Due to concerns about heterogeneity, imprecision, and publication bias | 5 (4–6) |
| Remifentanil as the sole opioid without RA | n = 9 | n = 234 | 62.359 (29.281, 97.032) | ✚✚◯◯  Low Due to concerns about heterogeneity, imprecision, and publication bias | 6 (5–6) |

**Table S9.** Summary of network meta-analysis findings for post-anaesthesia care unit stay (minutes). Techniques are listed in descending order of SUCRA values.

| Intervention | Number of studies n = 137 | Number of participants n = 11704 | Mean difference (95% credible interval) | Certainty of evidence* | Median ranking (95% credible interval) |
| --- | --- | --- | --- | --- | --- |
| Opioid-inclusive with RA | n = 88 | n = 3342 | Reference | Reference | 2 (1–3) |
| Remifentanil as the sole opioid with RA | n = 15 | n = 678 | 2.447 (-15.648, 20.542) | ✚✚◯◯  Low Due to concerns about heterogeneity, imprecision, and publication bias | 2 (1–5) |
| Opioid-free with RA | n = 19 | n = 1123 | 4.743 (-8.077, 17.585) | ✚✚◯◯  Low Due to concerns about heterogeneity, imprecision, and publication bias | 3 (1–5) |
| Opioid-free without RA | n = 21 | n = 1052 | 13.448 (0.495, 26.516) | ✚✚◯◯  Low Due to concerns about heterogeneity, imprecision, and publication bias | 4 (2–6) |
| Remifentanil as the sole opioid without RA | n = 23 | n = 895 | 14.564 (-0.009, 29.249) | ✚✚◯◯  Low Due to concerns about heterogeneity, imprecision, and publication bias | 5 (3–6) |
| Opioid-inclusive without RA | n = 109 | n = 4614 | 17.225 (11.363, 23.255) | ✚✚◯◯  Low Due to concerns about heterogeneity, imprecision, and publication bias | 5 (4–6) |

**Table S10.** Summary of network meta-analysis findings for hospital length of stay (hours). Techniques are listed in descending order of SUCRA values.

| Intervention | Number of studies n = 201 | Number of participants n = 19748 | Mean difference (95% credible interval) | Certainty of evidence* | Median ranking (95% credible interval) |
| --- | --- | --- | --- | --- | --- |
| Remifentanil as the sole opioid with RA | n = 17 | n = 650 | -16.978 (-61.736, 27.822) | ✚✚◯◯  Low Due to concerns about heterogeneity, imprecision, and publication bias | 1 (1–6) |
| Opioid-inclusive with RA | n = 151 | n = 7459 | Reference | Reference | 3 (1–5) |
| Remifentanil as the sole opioid without RA | n = 26 | n = 1027 | -3.311 (-40.975, 34.192) | ✚✚◯◯  Low Due to concerns about heterogeneity, imprecision, and publication bias | 3 (1–6) |
| Opioid-free with RA | n = 31 | n = 1588 | 4.520 (-21.602, 30.710) | ✚✚◯◯  Low Due to concerns about heterogeneity, imprecision, and publication bias | 4 (1–6) |
| Opioid-free without RA | n = 13 | n = 898 | 10.767 (-26.493, 48.519) | ✚✚◯◯  Low Due to concerns about heterogeneity, imprecision, and publication bias | 5 (1–6) |
| Opioid-inclusive without RA | n = 165 | n = 8126 | 15.674 (5.041, 26.476) | ✚✚◯◯  Low Due to concerns about heterogeneity, imprecision, and publication bias | 5 (3–6) |

**Table S11.** Summary of network meta-analysis findings for the incidence of postoperative nausea and vomiting. Techniques are listed in descending order of SUCRA values.

| Intervention | Number of studies n = 503 | Number of participants n = 44326 | Odds ratio (95% credible interval) | Certainty of evidence* | Median ranking (95% credible interval) |
| --- | --- | --- | --- | --- | --- |
| Opioid-free with RA | n = 92 | n = 3992 | 0.540 (0.411, 0.706) | ✚✚◯◯  Low Due to concerns about heterogeneity and publication bias | 1 (1–1) |
| Opioid-free without RA | n = 48 | n = 2182 | 0.860 (0.598, 1.235) | ✚✚◯◯  Low Due to concerns about heterogeneity, imprecision, and publication bias | 2 (2–4) |
| Opioid-inclusive with RA | n = 375 | n = 15526 | Reference | Reference | 3 (2–4) |
| Remifentanil as the sole opioid with RA | n = 41 | n = 1622 | 1.043 (0.648, 1.685) | ✚✚◯◯  Low Due to concerns about heterogeneity, imprecision, and publication bias | 4 (2–4) |
| Remifentanil as the sole opioid without RA | n = 58 | n = 3535 | 2.058 (1.353, 3.158) | ✚✚◯◯  Low Due to concerns about heterogeneity and publication bias | 5 (5–6) |
| Opioid-inclusive without RA | n = 399 | n = 17469 | 2.250 (1.981, 2.561) | ✚✚◯◯  Low Due to concerns about heterogeneity and publication bias | 6 (6–6) |

**Table S12.** Summary of network meta-analysis findings for the incidence of postoperative dizziness. Techniques are listed in descending order of SUCRA values.

| Intervention | Number of studies n = 40 | Number of participants n = 3141 | Odds ratio (95% credible interval) | Certainty of evidence* | Median ranking (95% credible interval) |
| --- | --- | --- | --- | --- | --- |
| Opioid-free with RA | n = 6 | n = 181 | 0.746 (0.244, 2.099) | ✚✚◯◯  Low Due to concerns about relevant imprecision | 1 (1–3) |
| Opioid-inclusive with RA | n = 27 | n = 1028 | Reference | Reference | 2 (1–3) |
| Opioid-free without RA | n = 4 | n = 181 | 2.599 (0.692, 8.832) | ✚✚◯◯  Low Due to concerns about relevant imprecision | 4 (1–4) |
| Opioid-inclusive without RA | n = 27 | n = 957 | 2.576 (1.690, 4.142) | ✚✚✚◯  Moderate Due to concerns about imprecision | 3 (3–5) |
| Remifentanil as the sole opioid with RA | n = 7 | n = 379 | 14.939 (1.282, 157.776) | ✚✚✚◯  Moderate Due to concerns about imprecision | 5 (3–5) |
| Remifentanil as the sole opioid without RA | n = 9 | n = 415 | 38.851 (4.677, 345.242) | ✚✚✚◯  Moderate Due to concerns about imprecision | 6 (5–6) |

**Table S13.** Summary of network meta-analysis findings for the incidence of postoperative pruritus. Techniques are listed in descending order of SUCRA values.

| Intervention | Number of studies n = 66 | Number of participants n = 4948 | Odds ratio (95% credible interval) | Certainty of evidence* | Median ranking (95% credible interval) |
| --- | --- | --- | --- | --- | --- |
| Opioid-free without RA | n = 6 | n = 228 | 0.090 (0.006, 0.927) | ✚✚✚◯  Moderate Due to concerns about imprecision | 1 (1–4) |
| Opioid-free with RA | n = 21 | n = 784 | 0.132 (0.036, 0.410) | ✚✚✚◯  Moderate Due to concerns about imprecision | 2 (1–4) |
| Remifentanil as the sole opioid without RA | n = 10 | n = 359 | 0.573 (0.030, 11.330) | ✚✚◯◯  Low Due to concerns about relevant imprecision | 4 (1–6) |
| Remifentanil as the sole opioid with RA | n = 8 | n = 298 | 0.854 (0.042, 18.097) | ✚✚◯◯  Low Due to concerns about relevant imprecision | 4 (1–6) |
| Opioid-inclusive without RA | n = 39 | n = 1402 | 0.938 (0.438, 2.012) | ✚✚◯◯  Low Due to concerns about relevant imprecision | 5 (3–6) |
| Opioid-inclusive with RA | n = 49 | n = 1877 | Reference | Reference | 5 (3–6) |

**Table S14.** Summary of network meta-analysis findings for the incidence of postoperative urinary retention. Techniques are listed in descending order of SUCRA values.

| Intervention | Number of studies n = 25 | Number of participants n = 2084 | Odds ratio (95% credible interval) | Certainty of evidence* | Median ranking (95% credible interval) |
| --- | --- | --- | --- | --- | --- |
| Opioid-free without RA | n = 2 | n = 83 | 0 (0, 0) | ✚✚✚◯  Moderate  Due to concerns about heterogeneity | 1 (1–2) |
| Remifentanil as the sole opioid with RA | n = 2 | n = 156 | 0 (0, 0.033) | ✚✚✚◯  Moderate  Due to concerns about heterogeneity | 2 (1–3) |
| Remifentanil as the sole opioid without RA | n = 4 | n = 172 | 0 (0, 0.057) | ✚✚✚◯  Moderate  Due to concerns about heterogeneity | 3 (2–3) |
| Opioid-free with RA | n = 5 | n = 207 | 0.315 (0.011, 5.965) | ✚✚✚◯  Moderate  Due to concerns about heterogeneity | 4 (4–6) |
| Opioid-inclusive without RA | n = 20 | n = 786 | 0.960 (0.222, 3.720) | ✚✚✚◯  Moderate  Due to concerns about heterogeneity | 5 (4–6) |
| Opioid-inclusive with RA | n = 17 | n = 680 | Reference | ✚✚✚◯  Moderate  Due to concerns about heterogeneity | 5 (4–6) |
